# Renal Clearable Luminogenic Reporter for Ultrasensitive Influenza Virus Imaging and Efficient Antiviral Therapies Monitoring in Living Mice

**DOI:** 10.1002/advs.76669

**Published:** 2026-07-17

**Authors:** Bankang Ruan, Chudan Liang, Meilin Hu, Weiping Xu, Jingjing Che, Jun Qian, Bingcai Jiang, Linna Liu, Xiancai Ma, Jiaguo Huang

**Affiliations:** ^1^ State Key Laboratory of Anti‐Infective Drug Discovery and Development School of Pharmaceutical Sciences Sun Yat‐sen University Guangzhou China; ^2^ Department of Ophthalmology Guizhou Provincial People's Hospital Guizhou China; ^3^ Guangzhou Key Laboratory of Clinical Pathogen Research for Infectious Diseases Institute of Infectious Disease Guangzhou Eighth People's Hospital Guangzhou Medical University Guangzhou China; ^4^ South China Center For Biosafety Sun Yat‐sen University Guangzhou China; ^5^ Institute of Human Virology, Zhongshan School of Medicine Sun Yat‐sen University Guangzhou China; ^6^ Guangzhou National Laboratory Guangzhou International Bio‐Island Guangzhou China; ^7^ State Key Laboratory of Structural Chemistry Fujian Institute of Research on the Structure of Matter Chinese Academy of Sciences Fuzhou China; ^8^ School of Public Health (Shenzhen) Shenzhen Campus of Sun Yat‐Sen University Shenzhen China; ^9^ Shenzhen Key Laboratory of Pathogenic Microbes and Biosafety Shenzhen China

**Keywords:** fluorescence sensing, fluorescence, in vivo, infection diagnosis, optical imaging, protease, urinalysis, viral replication

## Abstract

Influenza, which causes respiratory tract infections and related complications, poses a major threat to global public health. However, the rapid and accurate detection of influenza viruses in controlling the flu pandemic remains challenging, as current diagnostic methods are static and unable to distinguish between viable and nonviable virus or directly monitor viral replication dynamics. Herein, we report viral luminogenic reporters (VLRs) with chemiluminescence/fluorescence dual‐response for non‐invasive imaging and urinalysis of the H1N1 virus protease. VLR comprises a bicyclic dioxetane chemiluminophore signaling scaffold caged by a N‐acetylneuraminic acid, which further hooks a renal clearable moiety (2‐hydroxypropyl)‐β‐cyclodextrin. VLR achieves a limit of detection of 2.62 CCID_50_/mL in H1N1 virus detection, which was 10.4‐fold and 529.8‐fold lower than that of ICA‐qPCR and PMA‐qPCR assays. After intratracheal administration into H1N1 virus‐infected mice, VLR can efficiently accumulate in the lungs and specifically react with neuraminidase to restore its near‐infrared chemiluminescent/fluorescent signals for real‐time imaging. Leveraging the renal clearance (∼94% ID), VLR allows for remote detection of H1N1 virus infections and monitoring of antiviral therapeutic efficacy through in vitro urinalysis. Therefore, this study highlights a significant advance in addressing the critical gap in dynamic monitoring of virus replication activity and transforming virus‐specific probes into urinary reporters.

## Graphical abstract

A viral luminogenic reporter with chemiluminescence/fluorescence dual‐response and efficient renal clearance was developed for non‐invasive imaging and urinalysis of H1N1 virus protease, allowing for remote detection of H1N1 virus infections and monitoring of antiviral therapeutic efficacy in living mice.

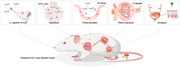

## 1 Introduction

Influenza viruses, which cause both upper and lower respiratory tract infections, have long presented a significant threat to global public health [1]. Seasonal influenza epidemics are characterized by their rapid and effortless transmission, affecting 10%–20% of the global population and resulting in over one billion infections worldwide each year [2]. Influenza‐associated complications, such as secondary bacterial pneumonia, dehydration, asthma, sinusitis, and congestive heart failure, can significantly elevate mortality rates, particularly among high‐risk groups [3]. Thus, precise diagnostic approaches and effective intervention strategies for disease control are of pivotal importance in preventing potential influenza pandemics [4].

Despite the urgent clinical need, dynamic monitoring of influenza viruses using conventional diagnostic techniques such as viral culture and polymerase chain reaction (PCR) is hindered by significant limitations [5, 6]. Classical viral culture is well‐known for being time‐consuming and labor‐intensive. In contrast, PCR enables the detection of viral ribonucleic acids with high sensitivity and specificity [7, 8]. However, it entails cumbersome procedures, sophisticated laboratory infrastructure, and highly trained technicians, with results potentially compromised by trace contaminants. Serological monoclonal antibody‐based assays, including antigen rapid tests (ARTs) and enzyme‐linked immunosorbent assay (ELISA), are valuable for detecting past infections [9, 10]; however, they are still prone to a high risk of false negative results. Furthermore, the aforementioned diagnostic methods are static, failing to distinguish between viable and nonviable viruses or to characterize viral replication activity. Consequently, in vivo monitoring of the dynamics and localization of viral replication is highly desirable but remains largely uninvestigated.

Molecular optical imaging provides noninvasive, real‐time strategies for monitoring the abundance, localization, and replication activity of influenza viruses in living systems, emerging as a promising alternative approach [11, 12, 13, 14, 15, 16, 17, 18, 19, 20, 21, 22, 23, 24, 25, 26, 27, 28, 29, 30, 31, 32, 33, 34, 35]. Although optical probes have been extensively developed for imaging various disease‐related biomarkers associated with cancer, organ injury, inflammation, and other pathologies, few agents have been reported for tracking the fate of influenza viruses [36, 37, 38, 39]. A limited number of probes have been developed to visualize viral infections; however, these probes are typically limited to monitoring immune activation following viral infection or detecting virus‐associated inflammatory events, such as fluctuations in reactive oxygen/nitrogen species (ROS/RNS) [40]. Virus‐specific probes targeting viral biomarkers (e.g., envelope proteins or nucleic acids) have also been described, yet most of these are restricted to in vitro cellular imaging [41, 42, 43, 44, 45, 46, 47]. More importantly, optical urinalysis holds greater promise for clinical translation compared with in vivo optical imaging, as it circumvents the issue of limited tissue penetration inherent to fluorescence imaging. Nevertheless, its application for monitoring viral replication dynamics remains unexplored, representing a critical research and clinical gap in this field.

**FIGURE 1:**
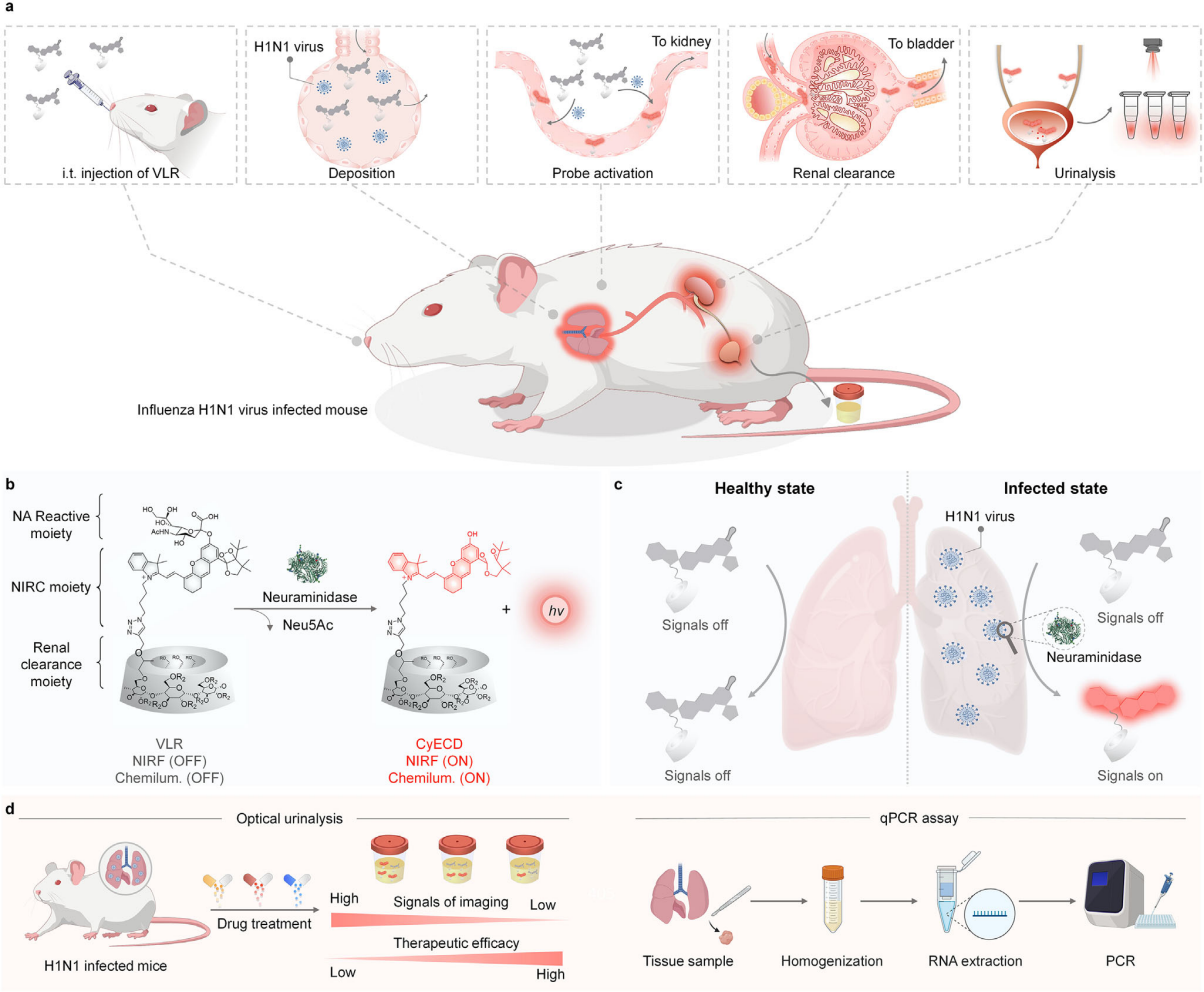
Design and mechanisms of VLR for real‐time imaging and urinalysis of influenza H1N1 virus and the efficacy of antiviral therapies in living mice. (a) Schematic illustration of noninvasive imaging and urinalysis of influenza H1N1 virus infection using VLR. After intratracheal (i.t.) administration of VLR into living mice, it accumulates into the lungs and specifically liberates NIRC signal to report NA levels, followed by effectively releasing the NIRF reporter (CyECD) to the kidneys, finally excreting out for fluorescently urinalysis. (b) Chemical structures of VLR and its fragments in response to NA. (c) In the healthy state, VLR was non‐chemiluminescence and non‐fluorescence. In the infected state, VLR in response to upregulated neuraminidase, liberating chemiluminescence signal and releasing fluorescently renal clearable CyECD for imaging and urinalysis. (d) Schematic illustration of assessment of antiviral therapeutic efficacy using VLR‐based urinalysis and qPCR assay.

Herein, we report the development of **V**iral **L**uminogenic **R**eporter (**VLR**) with chemiluminescence‐fluorescence dual‐response and efficient renal clearance for non‐invasive imaging and urinalysis of influenza H1N1 virus and evaluating the efficacy of antiviral therapies in living mice (Figure 1a). Neuraminidase (NA), one of the main proteins on the surface of the influenza H1N1 virus, plays crucial roles in processes of virus particle adhesion, entry, and release during viral replication in host cells. Moreover, NA has also been established as a promising target for both in vivo diagnostic and antiviral therapeutic target. Specifically, NA enzymatically cleaves terminal sialic acid residues from glycoproteins and glycolipids of host cells, as well as from newly formed virions, thereby facilitating viral release and spread of progeny virions. Therefore, we selected NA as the biomarker for signal activation of VLR. VLR comprises a bicyclic dioxetane chemiluminophore signaling scaffold locked by a NA substrate, N‐acetylneuraminic acid (Neu5Ac), which further hooks a renal clearable moiety (2‐hydroxypropyl)‐β‐cyclodextrin (HPβCD) (Figure 1b).

In the intrinsic state, VLR is non‐chemiluminescent and non‐fluorescent due to the inhibited intramolecular charge transfer (ICT) process (Figure 1b). After intratracheal (i.t.) administration into the lungs of live influenza‐infected mice (Figure 1a), VLR can efficiently accumulate in the lungs, specifically reacting with NA to restore its near‐infrared (NIR) chemiluminescent/fluorescent signals for real‐time imaging (Figure 1c). With its high urinary recovery efficiency (∼94%, injected dose (ID)), VLR can be rapidly excreted through the kidneys for sensitive urinary testing. In this study, we first construct VLR and deploy it in imaging‐based screening to identify antiviral drugs. We further validate its reliability for real‐time intravital imaging and urinalysis of influenza H1N1 virus either in an artificial NA‐positive mouse model or an H1N1 virus‐infected mouse model. At last, we demonstrate the feasibility of VLR‐based urinalysis for assessment of antiviral therapeutic effects (Figure 1d).

## 2 Results

### 2.1 Synthesis and Characterization

**FIGURE 2:**
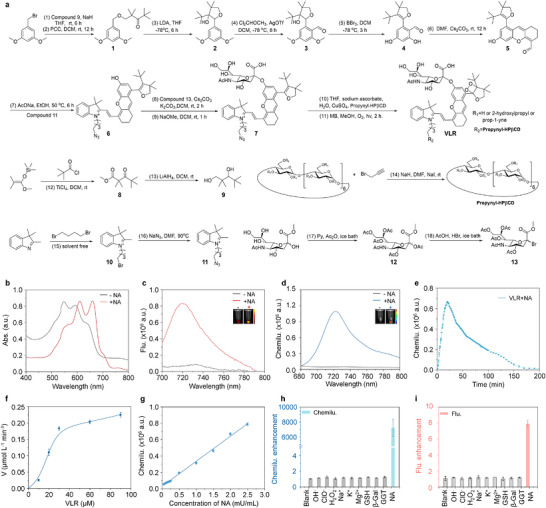
Synthesis and in vitro characterization. (a) Synthetic routes of VLR. Reagent and conditions: (1) Compound **9**, sodium hydride, THF, rt, 6 h. (2) Pyridinium chlorochromate, DCM, rt, 12 h. (3) Lithium diisopropylamide, THF, −78°C, 6 h. (4) Silver trifluoromethanesulfonate, dichloromethyl methyl ether, DCM, −78°C, 8 h. (5) Boron tribromide, DCM, −78°C, 3 h. (6) DMF, Cs_2_CO_3_, rt, 12 h. (7) Compound **11**, AcONa, EtOH, 50°C, 6 h. (8) Compound **13**, Cs_2_CO_3_, K_2_CO_3_, DCM, rt, 2 h. (9) NaOMe, DCM, rt, 1 h. (10) Propynyl‐HPβCD, sodium ascorbate, CuSO_4_, THF, H_2_O, rt, 2 h. (11) Methylene blue, MeOH, O_2_, hv, 2 h. (12) Titanium tetrachloride, DCM, rt. (13) Lithium Aluminum Hydride, THF, 0°C. (14) Sodium hydride, DMF, Sodium iodide, rt. (15) Solvent‐free, 50°C. (16) Sodium azide, DMF, 90°C. (17) Pyridine, acetic anhydride, ice bath. (18) Acetic acid, hydrobromic acid, ice bath. (b–d) Absorption, fluorescence, and chemiluminescence spectra of VLR (20 µm) in the absence or presence of NA (40 mU) in PBS (10 mm, pH 7.4). Inset: The corresponding fluorescence and chemiluminescence images using the IVIS spectrum imaging system. (e) Chemiluminescence kinetic profiles of VLR in the presence of NA in PBS (10 mm, pH 7.4). (f) Michaelis‐Menten saturation curve for NA (10 mU) toward VLR (10, 20, 30, 60, and 90 µm) in PBS (10 mm, pH 7.4). (*n* = 3, mean ± s.d.). (g) Chemiluminescence intensities of VLR as a function of the concentration of NA (0–3 mU/mL). (*n* = 3, mean ± s.d.). (h, i) Chemiluminescence and fluorescence changes of VLR (10 µm) after incubation with ROS (40 µm), metal ions (40 µm), glutathione (GSH), β‐galactosidase (β‐Gal), gamma‐glutamyl transferase (GGT), and NA in PBS (10 mm, pH 7.4). (*n* = 3, mean ± s.d.).

VLR comprised of three key components (Figure 1b): a hemicyanine‐based bicyclic dioxetane as an NIR chemiluminescence (NIRC) moiety, an alkyne‐functionalized HPβCD as a renal clearable moiety, and a neuraminidase‐responsive moiety (α‐D‐N‐acetylneuraminic acid, Neu5Ac). The synthetic routes of VLR were shown in Figure S1a. Briefly, the key fluorophore **6** was synthesized from commercial 3,5‐dimethoxybenzyl bromide according to our previous studies [31]. Then compound **6** was substituted by compound **13,** and then hydrolysis for deprotection to yield compound **7**. Subsequently, the resulting compound **7** underwent a click reaction with alkyne‐functionalized HPβCD and oxidation via [2 + 2] cycloaddition with singlet oxygen (^1^O_2_) using methylene blue as the photosensitizer to afford probe VLR. The synthetic routes of other intermediates were also shown in Figure 2a and Figure S1b–e. The chemical structures of the intermediates and the final probe were confirmed by nuclear magnetic resonance (NMR) and mass spectrometry (MS).

The photophysical properties of VLR were measured. In the absence of NA, VLR exhibited a peak absorption at 550 nm, along with minimal chemiluminescence and fluorescence (Figure 2b–d). This phenomenon was attributed to the terminal N‐acetylneuraminic acid masking the phenolic hydroxyl group, thereby inhibiting the formation of phenolate anions and blocking both the electron transfer pathway and intramolecular charge transfer (ICT). Under these conditions, the high‐energy peroxide bridge remains stable, and no luminescence signal is detected. In the presence of NA, the maximum absorption underwent a redshift from approximately 550 to 680 nm (Figure 2b). Meanwhile, a dramatic enhancement was observed in the signals at 720 nm, showing a ∼7100‐fold increase in chemiluminescence and a ∼8‐fold increase in fluorescence (Figure 2c,d). Such dramatic emission enhancement was attributed to NA specifically hydrolyzes the glycosidic bond of the probe to liberate phenolate anions. This cleavage triggers intramolecular electron transfer, leading to the decomposition of the peroxide bridge and the generation of excited‐state intermediates, which emit photons upon relaxing back to the ground state. Concurrently, the restoration of the electron‐donating ability of the aromatic hydroxyl group elicits a robust turn‐on fluorescence response. The chemiluminescence half‐life of VLR was about 16 min (min) (Figure 2e). The catalytic efficiency (K_cat_/K_m_) of NA toward VLR was calculated to be 0.82 µm ^−1^S^−1^ (Figure 2f). A strong linear correlation was observed between the chemiluminescent signal and NA concentration, with an estimated limit of detection (LOD) of approximately 12 mU/L (Figure 2g). Furthermore, VLR showed no obvious enhancement in chemiluminescence and fluorescence in the presence of other analytes, including reactive oxygen species (ROS), metal ions, or enzymes, demonstrating its high specificity (Figure 2h,i). In addition, the thermal stability of the bicyclic dioxetane (CL‐1) and adamantyl dioxetane (CL‐2) was evaluated through measurement of their residual chemiluminescence when stored at room temperature. As shown in Figure S2a–b, CL‐1 demonstrated superior thermal stability than that of CL‐2. Zeta potential measurement revealed that VLR was negatively charged (Figure S3a). No obvious change in fluorescence for VLR was observed in phosphate buffer solution (PBS) or fetal bovine serum (FBS) for 5 days (Figure S3b) and in different pH buffer solutions (Figure S3c), confirming its high stability.

### 2.2 Visualization of NA Expression in Influenza H1N1 Virus‐Infected Cells

**FIGURE 3:**
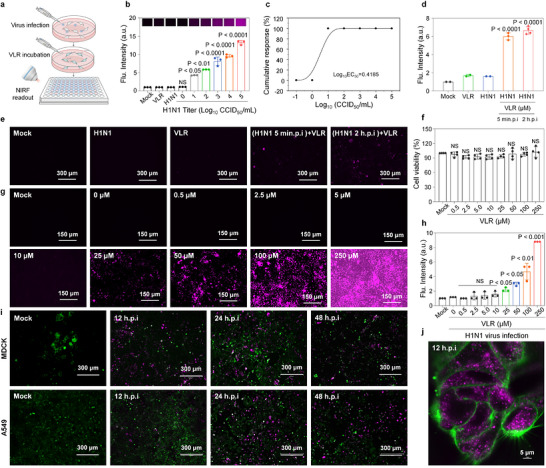
Imaging of NA expression in H1N1 influenza virus‐infected cells. (a) Schematic illustration of the experimental flow chart for VLR (50 µm) to imaging of NA in living infected cells. (b) Cell viability of MDCK cells after treatment with VLR at different concentrations from 0.5 to 250 µm. (c) Corresponding fluorescence intensity of different H1N1 virus titers incubated with VLR (50 µm). (d) Cumulative response of H1N1 virus incubated with VLR (50 µm) by probit analysis. (e) Fluorescence images of proliferation after treatment with VLR (50 µm) in living cells. (Scale bar: 300 µm) (f) Corresponding fluorescence intensity of MDCK cells in the panel of (e). (g, h) Fluorescence images and intensity of H1N1‐infected MDCK cells after treatment with VLR at different concentrations from 0.5 to 250 µm (Scale bar: 150 µm). (i) Fluorescence images of H1N1‐infected MDCK and A549 cells after treatment with VLR (50 µm). (Scale bar: 300 µm). (j) Confocal fluorescence microscopy images of MDCK cells incubated with VLR (50 µm) (Scale bar: 5 µm). The magenta fluorescence represented NA‐positive expression virus, and the green fluorescence indicated a lipophilic cell membrane.

We next evaluated the performance of VLR for imaging neuraminidase (NA) expression in virus‐infected cells (Figure 3a). To assess the detection sensitivity of VLR for the detection of H1N1 influenza virus, we incubated VLR with various titers of infectious viruses (Figure 3b,c). The limit of detection for H1N1 influenza virus was determined to be as low as 2.62 CCID_50_/mL, which was 10.38‐fold and 529.77‐fold lower than that of integrated cell absorption quantitative polymerase chain reaction (ICA‐qPCR) and combined propidium monoazide qPCR (PMA‐qPCR) assays (Figure S4a,b), demonstrating the excellent sensitivity of VLR for the direct detection of viable infectious viruses. Following incubation of infected cells with VLR, we found that the activation of VLR occurred rapidly in 5 min in infected MDCK cells, as evidenced by the strong NIRF signal detected after a 5‐min incubation, which also indicated the rapid invasion of host cells by infectious influenza viruses (Figure 3d,e). VLR by itself demonstrated excellent cytocompatibility even at a concentration as high as 250 µm (Figure 3f). The VLR‐recognized viral NA protein showed magenta fluorescence in cells, with favorable imaging effects observed at concentrations of probe ranging from 25 to 100 µm (Figure 3g–j). In addition to the H1N1 virus, VLR was capable of dynamic imaging of the replication of other NA‐positive expression viruses in both MDCK and A549 cells, including H3N2 and IBV viruses (Figure 3i and Figure S5a,b), as both were expressed with NA proteins, demonstrating the applicability of VLR in imaging of NA‐positive expression viruses.

### 2.3 Distinguishing Between Viable and Nonviable H1N1 Virus and Screening of Antiviral Drugs

**FIGURE 4:**
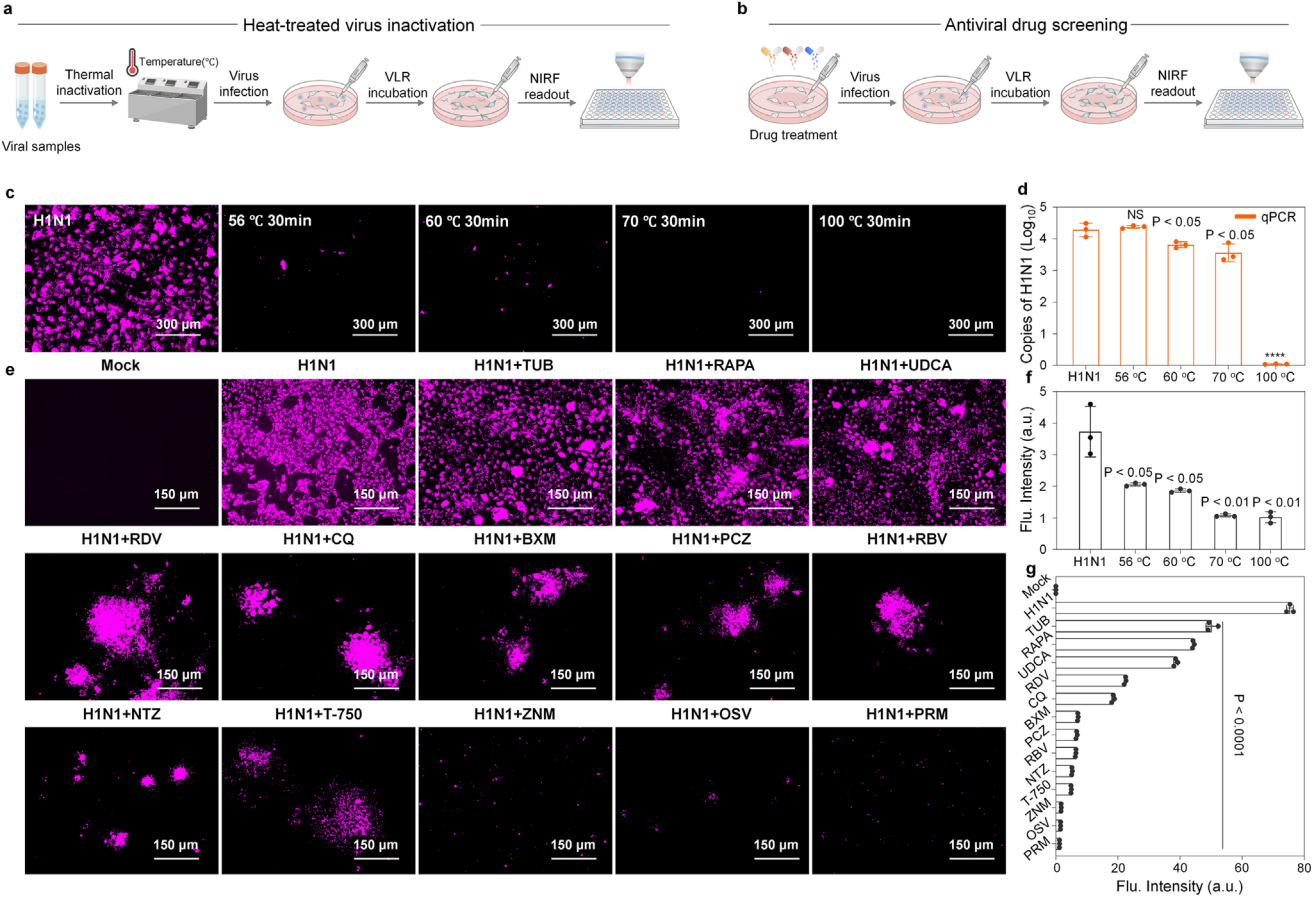
In vitro imaging of VLR in living cells with drug treatment. (a) Schematic illustration of experiment implementation for VLR (50 µm) to image thermal inactivated virus in living cells. (b) Schematic illustration of experiment implementation for VLR (50 µm) to image virus in living cells with drug treatment. (c) Fluorescence images of H1N1 at different inactivation temperature infected MDCK cells after treatment with VLR (50 µm). (Scale bar: 300 µm). (d) Copy numbers of H1N1 virus (Log_10_) with different inactivation temperatures. (e) Fluorescence images of H1N1 virus‐infected MDCK cells after treatment with VLR (50 µm) at different drug treatment. (Scale bar: 150 µm). 10 µm tubeimoside (TUB), 1 µm rapamycin (RAPA), 100 µm ursodeoxycholic acid (UDCA), 100 µm remdesivir (RDV), 50 µm chloroquine (CQ), 1 µm baloxavir (BXM), 10 µm prochlorperazine (PCZ), 100 µm ribavirin (RBV), 30 µm nitazoxanide (NTZ), 100 µm favipiravir (T‐750), 10 µm zanamivir (ZNM), 50 µm oseltamivir (OSV), and 20 µm peramivir (PRM) were used. (f) Fluorescence intensity of H1N1 virus at different inactivation temperature infected MDCK cells after treatment with VLR (50 µm). (g) Fluorescence intensity in different drug treatment groups.

To assess the capability of VLR in distinguishing between viable and nonviable H1N1 virus, we inactivated the virus thermal treatment at different temperatures prior to cell infection and incubation (Figure 4a). VLR effectively differentiated viable from nonviable H1N1 virus across all tested conditions, showing reductions in near‐infrared fluorescence (NIRF) signals by 1.85‐fold, 2.0‐fold, 3.54‐fold, and 3.67‐fold for the 56°C, 60°C, 70°C, and 100°C treatment groups, respectively, compared with the positive control group (Figure 4c,f). However, there was only a slight decrease in the copy numbers of the H1N1 virus following thermal treatment at 70°C (Figure 4c,d,f), implying that quantitative polymerase chain reaction (qPCR) failed to discriminate viable and nonviable infectious H1N1 virus. Such observations were consistent with the results of the ICA‐qPCR method (Figure S6). Thus, VLR was superior to the qPCR assay in distinguishing between viable and nonviable H1N1 virus.

Real‐time fluorescence imaging provides a powerful tool for fast screening of antiviral drugs. Having established the efficacy of VLR in imaging of H1N1 virus, its capacity to screen antiviral drugs was evaluated through image‐based phenotypic assays (Figure 4b). H1N1 virus‐infected MDCK cells were pre‐treated with PBS/DMSO or various antiviral medications including tubeimoside (TUB), rapamycin (RAPA), ursodeoxycholic acid (UDCA), remdesivir (RDV), chloroquine (CQ), baloxavir (BXM), prochlorperazine (PCZ), ribavirn (RBV), nitazoxanide (NTZ), favipiravir (T‐750), zanamivir (ZNM), oseltamivir (OSV) and peramivir (PRM) (Figure S7), followed by incubation with VLR. NIRF signals of VLR in PBS‐treated or DMSO‐treated MDCK cells served as controls (Figure 4e). All medications‐treated cells exhibited a significant decrease in NIRF signal relative to the control group, while NIRF signals were attenuated to 2% in ZNM‐, OSV‐, and PRM‐treated cells due to their potent NA inhibitor effect (Figure 4g). Such observations were consistent with the results of the viral copy numbers (Figure S8), and decreased NIRF signals of VLR were observed with increasing concentrations of ZNM, OSV, and PRM; these three medications (Figure S9), suggesting that ZNM, OSV, and PRM‐induced superior therapeutic efficacy against the H1N1 virus as compared to other medications. Moreover, PCZ has exhibited potential antiviral activity against the influenza virus, which has not been previously reported, as evidenced by the results of NIRF observation and viral copy numbers (Figure 4e and Figure S8). Thus, the data validated that VLR holds great potential for high‐throughput preclinical antiviral drug screening.

### 2.4 In vivo Clearance Pathways and Biodistribution

**FIGURE 5:**
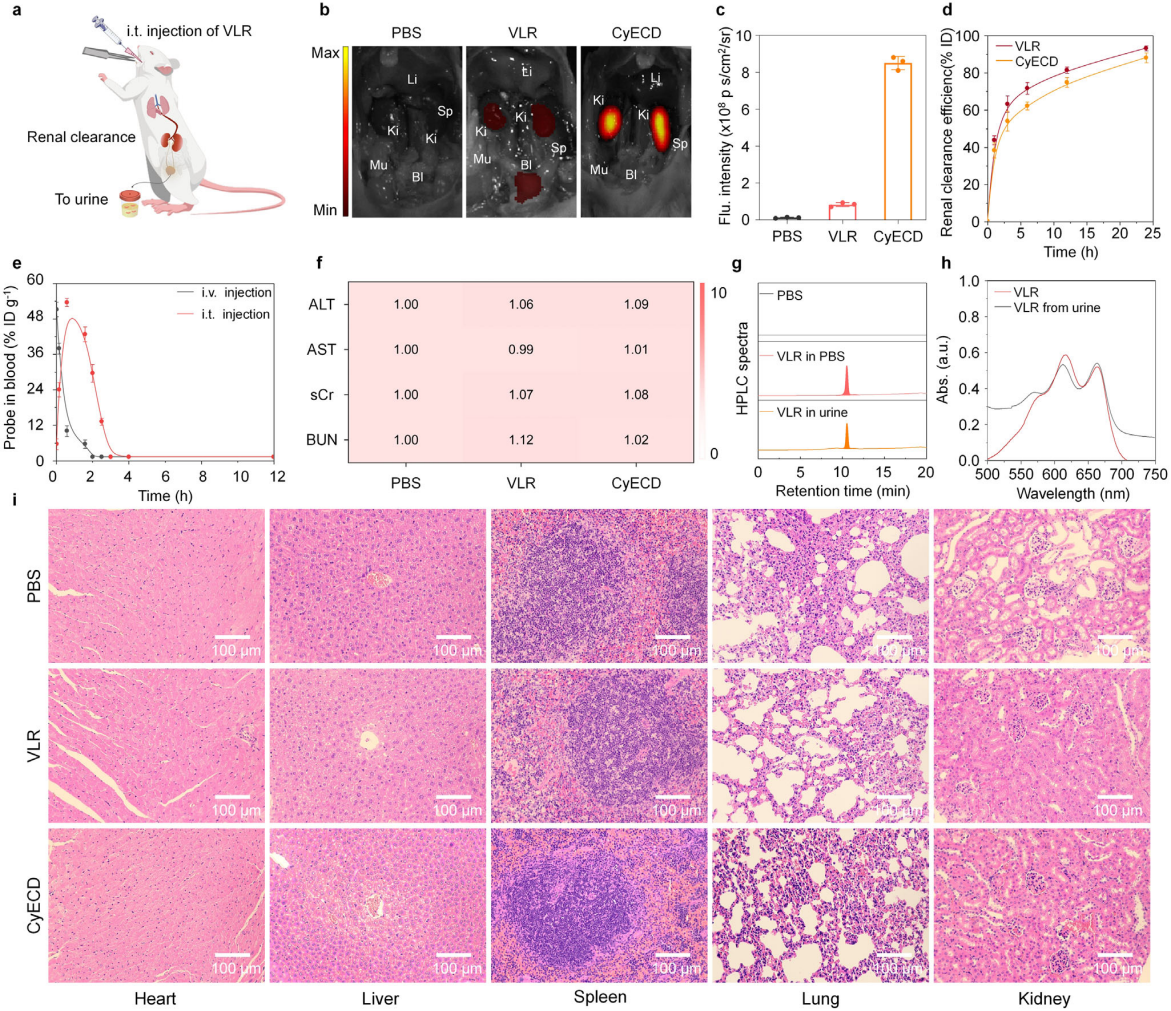
Biodistribution and biocompatibility (a) Schematic illustration of the renal clearance pathway of VLR in living mice. (b) Representative NIRF images of the abdominal cavity at 24 h post‐injection of PBS, VLR and CyECD (10 µmol kg^−1^ body weight). He: heart; Li: liver; Sp: spleen; Lu: lung; Ki: kidney; Bl: bladder. (c) Ex vivo NIRF quantification of the kidney at 24 h post‐injection of PBS, VLR and CyECD. The NIRF images were acquired at 720 nm upon excitation at 680 nm with the IVIS spectrum imaging system. (d) Renal clearance efficiency as a function of time post‐injection of VLR or CyECD into living mice. (*n* = 3, mean ± s.d.). (e) Blood concentration (% ID g^−1^) decay of VLR after i.v. or i.t. injection into living mice (*n* = 3, mean ± s.d.). (f) Measurements of liver and kidney functions in mice after 24 h injection of PBS, VLR and CyECD. (*n* = 3, mean± s.d.). (g, h) HPLC analysis and UV/Vis absorption of the urine samples excreted from living mice after i.t. injection of VLR. (i) HE staining of major organs including heart, liver, spleen, lung, and kidney from mice after 24 h injection of PBS, VLR, and CyECD (10 µmol kg^−1^ body weight). (Scale bar: 100 µm).

To investigate the biodistribution and pharmacokinetics of VLR and its activated probe CyECD in healthy living mice, fluorescence imaging of organs was imaged after i.t. injection of VLR and CyECD (Figure 5a). Strong fluorescence signal of CyECD was mainly observed in kidneys, with minimal NIRF signals were recorded in other organs (Figure 5b). Similar trends were observed for VLR (Figure 5b,c), but the NIRF signals in kidneys were much lower due to its intrinsic caged state with non‐fluorescent. Pharmacokinetics of VLR after intravenous (i.v.) or i.t. injection was investigated using high‐performance liquid chromatography (HPLC). The blood concentration curves showed that after i.v. injection, the concentration of VLR rapidly declined to 0% of the injected doses (ID) g^−1^ at 2 h post‐injection, while i.t. injection resulted in the increase of VLR within 1 h post‐injection and then decreased to 0% ID g^−1^ at 4 h post‐injection (Figure 5e). Therefore, i.t. injection of VLR had a much longer elimination half‐life (t_1/2β_) (∼1.8 h) compared to i.v. injection of VLR (∼28 min), confirming that i.t. injection of VLR provides a significantly prolonged imaging window. In addition, renal clearance efficiency of VLR and CyECD was calculated to be ∼94% ± 2% ID and ∼88% ± 2.6% ID at 24 h post‐injection in living mice, respectively (Figure 5d). Consistent with the above imaging results that VLR was mainly excreted through the kidneys, this excretion could be attributed to its high hydrophilicity (distribution coefficient Log *D* = −12.38) and small molecular weight (∼3 kDa), which is much lower than the glomerular filtration molecular weight cutoff (∼50 kDa) [48]. Moreover, the absorption and HPLC spectra of VLR recovered from urine were measured. VLR exhibited nearly identical optical spectra and HPLC spectra compared to its as‐synthesized form (Figure 5g,h), confirming minimal in vivo metabolism of VLR in healthy living mice. No increment in serological levels of alanine transaminase (ALT), aspartate transaminase (AST), serum creatinine (sCr), and blood urea nitrogen (BUN) was observed between VLR, CyECD, and PBS‐injected mice (Figure 5f and Figures S10 and S11). Furthermore, Histological staining revealed that these probes did not induce any histological changes, thereby confirming their high biosafety (Figure 5i). Ex vivo fluorescence imaging and biodistribution studies further confirmed these findings (Figure S12).

### 2.5 NIRF Imaging and Urinalysis of NA in Living Mice

**FIGURE 6:**
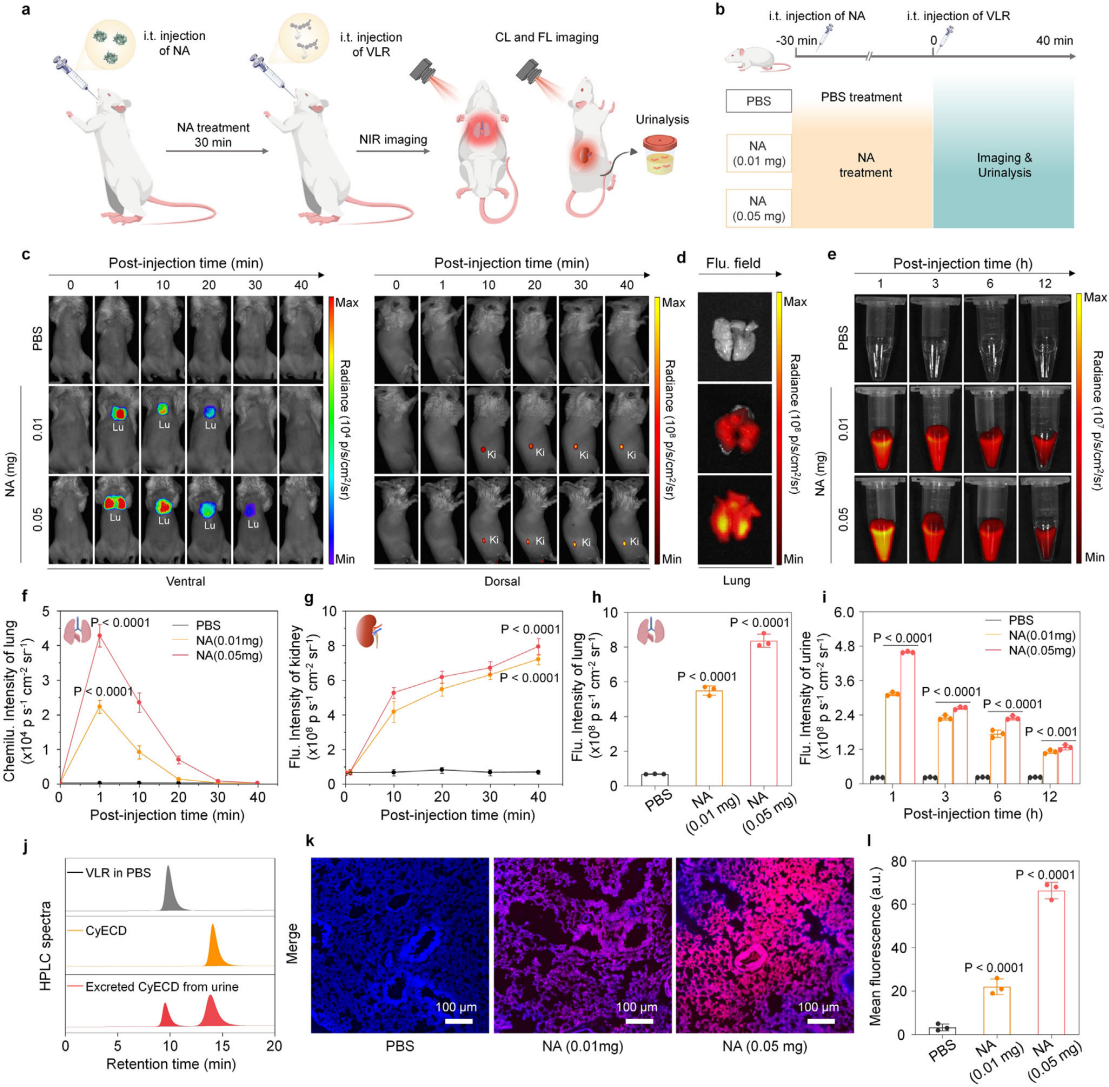
NIRF imaging and urinalysis of neuraminidase (NA) in living mice. (a) Scheme illustration of real‐time imaging and urinalysis of NA in an artificial NA‐positive mice model. (b) Schematic illustration of the establishment of the mouse model and imaging at different time points after NA treatment. (c) Representative chemiluminescence and fluorescence images of living mice after VLR injection. Chemiluminescence images acquired under bioluminescence mode of the IVIS spectrum imaging system with the acquisition time of 60 s, fluorescence images acquired at 720 nm upon excitation at 680 nm. Noted that the mice were injected with VLR and placed in the IVIS system for continual imaging at different post‐injection time points (1, 10, 20, 30, and 40 min) in panel c. Lung (Lu), Kidney (Ki). (d) Representative fluorescence images of resected lungs from different groups. (e) Representative fluorescence images of urine from living mice with different NA treatment groups. (f, g) Dynamic chemiluminescence and fluorescence intensities of lung and kidney as a function of time after injection of VLR. (h, i) Corresponding fluorescence intensity of lung and urine in the panel of (d) and (e). (j) HPLC analysis of the urine samples after i.t. injection of VLR and excretion from living mice. Trace of the pure compound and VLR incubated with NA are also indicated for comparison. (k) Confocal fluorescence microscopy images of lung slices from mice after treatment with PBS, NA (0.01 mg), or NA (0.05 mg). The blue signals come from DAPI staining. (scale bar = 100 µm). (l) Mean fluorescence intensity of activated VLR in the panel of (k) Data are the mean ± SD. *n* = 3 independent experiments. Two‐tailed Student's t‐test. PBS group vs. experimental groups (^*^ *p* < 0.05, ^**^ *p* < 0.01, ^***^ *p* < 0.001, ^****^ *p* < 0.0001).

The capability of VLR for real‐time imaging and urinalysis of NA was evaluated in an artificial NA‐positive mice model established via i.t. injection of commercially available recombinant NA (Figure 6a). Mice were treated with different doses of NA (0.01 mg or 0.05 mg per mouse) respectively to simulate H1N1 infection, followed by i.t. injection of VLR. The control mice were treated with PBS (Figure 6b). Whole‐body longitudinal chemiluminescence and fluorescence imaging were conducted concurrently at different time points post‐injection (Figure 6c). Within 1 min of VLR injection, robust chemiluminescence signals were detected in the lungs of mice that had received NA. The signal intensities were 118‐fold (0.01 mg NA) and 226‐fold (0.05 mg NA) higher than those in the control group (Figure 6f), confirming that VLR is activated through Neu5Ac cleavage in the presence of NA. These chemiluminescence signals gradually decreased to the background at 30 min post‐injection. Notably, increased NIRF signals were observed in the kidneys of mice injected with NA, indicating that VLR was activated by NA in the lungs, leading to the release of CyECD into the kidneys. In contrast, negligible NIRF signals were recorded in the control mice. At 40 min post‐injection of VLR, NIRF signals of kidneys from mice injected with NA were 10.7‐fold (0.01 mg NA) and 11.8‐fold (0.05 mg NA) higher than those of the control group, respectively (Figure 6g). To confirm the in situ activation of VLR in the lung, ex vivo NIRF imaging and immunostaining of lung tissue from mice were performed. Ex vivo fluorescence imaging and biodistribution studies corroborated those in vivo findings (Figure 6d and Figure S13a,b), showing NIRF signals of lungs were 8.2‐fold (0.01 mg NA) and 12.7‐ fold (0.05 mg NA) higher than those of the control group (Figure 6h). Similarly, increased NIRF signals were observed in the section of lung from mice receiving NA compared to the control group, as evidenced by 6.9‐fold and 19.8‐fold enhancement in red signals for 0.01 mg and 0.05 mg NA‐treated mice relative to the control group, respectively (Figure 6k,l). Moreover, the ability to remotely detect pulmonary NA via in vitro optical urinalysis was evaluated. 14.3‐fold and 21.4‐fold fluorescence enhancements in urine were observed for mice that received NA (0.01 and 0.05 mg) relative to those of the control group at 1 h post‐injection of VLR (Figure 6i). The activation of VLR and release of CyECD into urine were further confirmed by HPLC assay, showing that the retention times of excreted CyECD from urine were almost identical to those of the as‐synthesized one. (Figure 6e,j).

### 2.6 NIRF Imaging and Urinalysis of H1N1 Virus Infection Dynamics in Living Mice

Inspired by its excellent performance in imaging of NA in vivo, we further evaluated the ability of VLR for detection of H1N1 virus infection in living mice. The different viral doses (10^5^, 10^6^, and 10^7^ TCID_50_ H1N1 virus) for pulmonary injection were first assessed. Body weight and survival rate were monitored in comparison with the control mice receiving PBS. Compared with the control mice, all the virus‐infected mice showed significant decrements in body weight and bright chemiluminescence signals in the lungs after injection of VLR (Figures S14 and S15a). In addition, the mice administered with 10^5^ TCID_50_ of H1N1 viruses had a survival rate of ∼90%for 10 days, while the mice administered with higher virus doses (10^6^ and 10^7^ TCID_50_ of H1N1 viruses) died within 10 days (Figure S15b). We herein selected 10^5^ TCID_50_ of H1N1 viruses as the optimal dose for induction of infection and further imaging application because the host is more tolerant to this dose.

**FIGURE 7:**
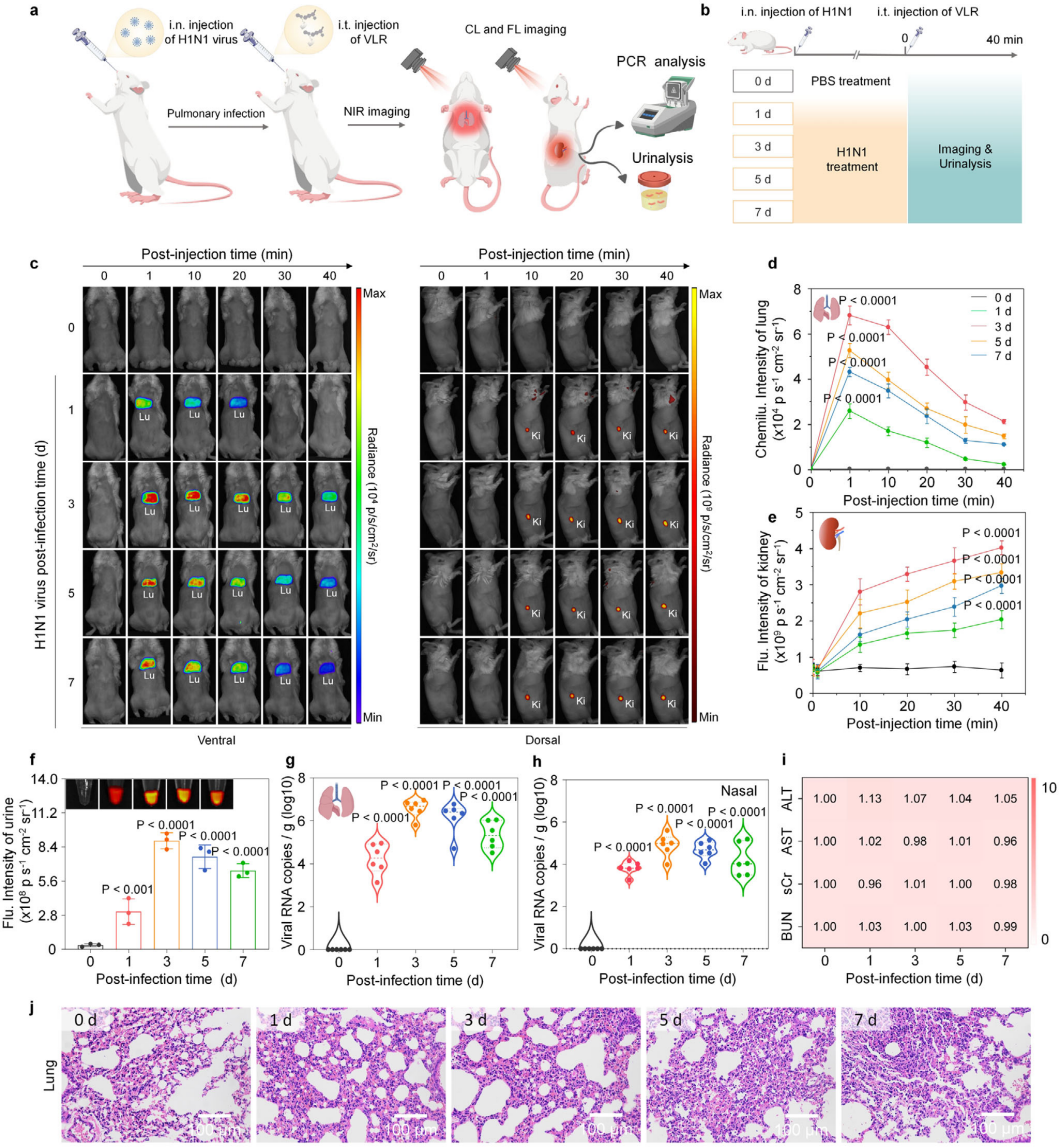
NIRF imaging and urinalysis of H1N1 virus infection dynamics in living mice. (a) Scheme illustration of in vivo imaging and urinalysis of H1N1 virus infection in living mice. (b) Schematic illustration of the establishment of the mouse model and imaging at different timepoints after H1N1 virus infection. (c) Representative chemiluminescence and fluorescence images of living mice after VLR injection at different post‐infection times. Chemiluminescence images acquired under bioluminescence mode of the IVIS spectrum imaging system with the acquisition time of 60 s, fluorescence images acquired at 720 nm upon excitation at 680 nm. Noted that the mice were injected with VLR and placed in the IVIS system for continual imaging at different post‐injection time points (1, 10, 20, 30, and 40 min) in panel c. Lung (Lu), Kidney (Ki). (d, e) Dynamic chemiluminescence and fluorescence intensities of lungs and kidneys as a function of time after injection of VLR. (f) Fluorescence intensities of urine from living mice after injection of VLR at different post‐infection times. Inset: the corresponding fluorescence images of the urine sample acquired at 720 nm upon excitation at 680 nm with an IVIS spectrum imaging system. (g, h) The copy number of H1N1 virus in the lungs and nasal at different post‐infection times. (i) Measurements of liver and kidney functions in mice at different post‐infection times. (*n* = 3, mean± s.d.). (j) HE staining of lungs from mice at different post‐infection times. (Scale bar: 100 µm).

VLR was i.t. injected into living mice at different infection time points (1, 3, 5, or 7 days) for longitudinal imaging and urinalysis of H1N1 virus (Figure 7a,b). Mice from the control group were treated with PBS. At 1‐day post H1N1 virus infection, the lung of living mice was observed via NIRC imaging (Figure 7c) with the maximum signal at 1 min post injection of VLR, which was 136.6‐fold higher relative to the control group (Figure 7d). Meanwhile, the kidneys were clearly delineated with NIRF imaging. These findings revealed that VLR was activated by the pulmonary H1N1 virus and excreted into the kidneys. Similar signal evolutions were observed for mice at 3‐, 5‐, and 7‐day post H1N1 virus infection (Figure 7c–e). However, the maximum signals of lungs (kidneys) at 3‐, 5‐, and 7‐days post infection were 360.9 (6.3), 278.3 (5.2), and 228.1 (4.6) times higher than those of control mice, respectively (Figure 7d,e), implying the successful H1N1 virus replication in the lungs after infection. Ex vivo NIRF imaging and biodistribution studies showed similar results (Figure S22). To assess the translational potential of VLR, the capability to remotely detect pulmonary H1N1 virus infection via optical urinalysis was evaluated in vitro. The kidney function was first assessed in all groups. No increase in sCr and BUN was observed between groups (Figure 7i and Figures S16 and S17), indicating that H1N1 virus infection did not affect kidney function. This ensured the identical renal clearance efficiency of CyECD during the detection period. Furthermore, Hematoxylin and Eosin (HE) staining of lungs revealed noticeable enlargement of alveolar spaces and thickening of alveolar walls after H1N1 virus infection (Figure 7j and Figure S20), while no significant lesion was shown in other major organs (Figure 7i and Figure S22). To correlate urinary signal with the levels of H1N1 viruses, NIRF signal was measured for excreted CyECD in urine at different infection time points (1, 3, 5, or 7 days) after injection of VLR into mice. 9.1, 26.9, 21.5, and 19.4‐fold fluorescence enhancements were observed for H1N1 virus‐infected mice at 1, 3, 5, or 7 days relative to control mice (Figure 7f), validating that VLR‐based urinalysis can monitor H1N1 virus infection dynamics.

To validate whether the signal of VLR correlated with the copy number of H1N1 virus, the levels of H1N1 virus in the lungs and nasal cavities from mice at different post‐infection times were analyzed by PCR. Consistent with VLR‐based imaging and urinalysis, 4.2, 6.5, 6.1, and 5.3‐fold increases in H1N1 virus copy number in the lungs were observed in mice at 1, 3, 5, or 7 days post‐infection relative to control mice (Figure 7g). A similar trend was observed in the nasal cavities (Figure 7h). Correlation studies revealed that a positive correlation between H1N1 virus copy number in the lungs and VLR‐based in vivo NIRC imaging (Pearson's r  =  0.9431, *p* < 0.0001), VLR‐based in vivo NIRF imaging (Pearson's r  =  0.9782, *p* < 0.0001), and VLR‐based urinalysis (Pearson's r  =  0.9348, *p* < 0.0001) (Figure S19a–c). To investigate whether VLR was specifically activated by the pulmonary H1N1 virus, urine samples were analyzed via PCR. No enhancement in H1N1 virus copy number relative to control mice was observed, confirming that negligible H1N1 virus was present in urine and the urinary NIRF signals were original from the activation of VLR in the lungs (Figure 7g,h and Figure S18). Moreover, lung section imaging was performed at different post‐infection time points after i.t. injection of VLR. The trend of NIRF signal from activated VLR was consistent with the green signal from NA antibody staining (Figure S21a,b), confirming the specificity of VLR for NA. Thus, VLR not only enables real‐time in vivo imaging of pulmonary H1N1 virus infection in living mice but also permits remote in vitro urinalysis.

### 2.7 NIRF Imaging and Urinalysis of the Efficacy of Antiviral Therapies in Living Mice

**FIGURE 8:**
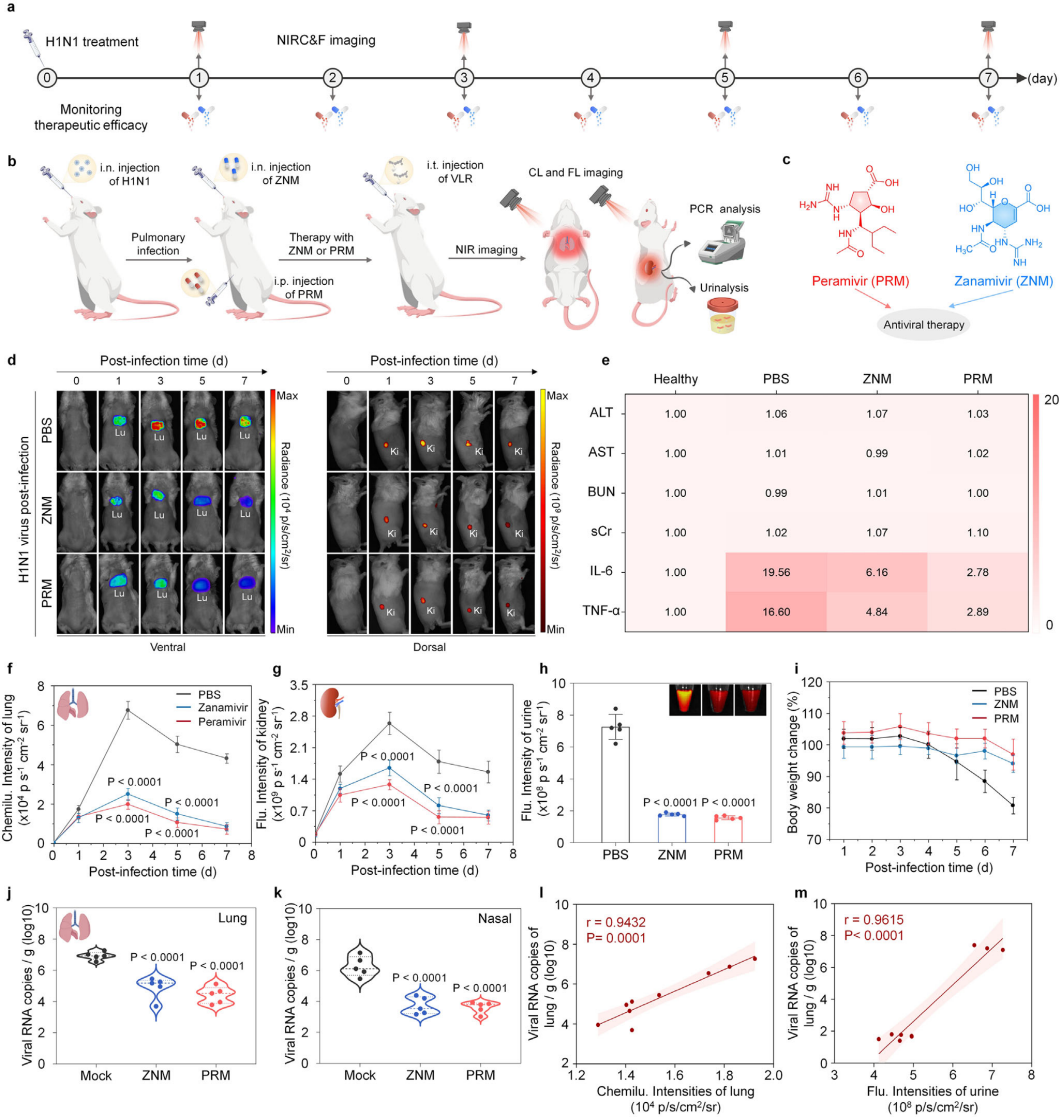
NIRF imaging and urinalysis of the efficacy of antiviral therapies in living mice. (a) Schematic illustration of the timeline of antiviral therapy and imaging/urinalysis to monitor treatment efficacy. (b) Scheme illustration of the experimental flow chart of imaging and urinalysis of antiviral therapeutic efficacy in living mice. (c) Chemical structures of antiviral medications, including peramivir (PRM) and zanamivir (ZNM). (d) Representative chemiluminescence and fluorescence images of living mice after VLR injection with different drug treatments. Chemiluminescence images acquired under bioluminescence mode of the IVIS spectrum imaging system with the acquisition time of 60 s, fluorescence images acquired at 720 nm upon excitation at 680 nm. Lung (Lu), Kidney (Ki). (e) Measurements of liver and kidney functions, and cytokines in mice with different drug treatments (*n* = 3, mean ± s.d.). (f, g) Dynamic chemiluminescence and fluorescence intensities of lungs and kidneys as a function of time after injection of VLR. (h) Fluorescence intensities of urine from infected mice with different drug treatments after injection of VLR. Inset: the corresponding fluorescence images of the urine sample acquired at 720 nm upon excitation at 680 nm with an IVIS spectrum imaging system. (i) Changes in body weight of living mice throughout the experimental period with different drug treatments. (j, k) The copy numbers of the virus in the lungs and nasal with different drug treatments. (l) Correlation between the copy number of virus in the lungs and the in vivo NIRC signals via a simple linear regression model. The 95% confidence intervals were obtained by two‐tailed Student's *t*‐test analysis. (m) Correlation between the copy number of virus in the lungs and the urinary NIRF signals via a simple linear regression model. The 95% confidence intervals were obtained by two‐tailed Student's *t*‐test analysis.

We then evaluated the capability of VLR to assess the effectiveness of antiviral therapies (Figure 8a). To alleviate H1N1 virus infection, H1N1 virus‐infected mice received daily administration of antiviral medications, including peramivir (PRM), zanamivir (ZNM), or PBS as the control group [49, 50]. Following various therapies, VLR was then injected into mice at 1, 3, 5, and 7 days post‐infection (Figure 8b,c). At 1 min and 40 min post‐injection of VLR, longitudinal in vivo NIRC and NIRF imaging were conducted to monitor signals from lungs and kidneys, respectively (Figure 8d). Moreover, urine samples were collected for optical urinalysis. Noted that the repetitive injection of VLR every two days did not cause the residual VLR in the body because of its high and rapid renal clearance efficiency (∼94%) within 24 h. In control mice receiving PBS, a gradual increase of NIRC and NIRF signals in the lungs and kidneys was respectively observed after H1N1 virus infection and reached the maximum at 3 days post‐infection, indicating viral replication after infection. Those signals gradually decreased at later timepoints, in parallel with the progression of infection and the subsequent enhancement of innate and then adaptive immune responses [51]. Similar trends were observed in mice after peramivir or zanamivir treatment. However, signals in lungs (or kidneys) from peramivir‐ or zanamivir‐treated mice were 3.4‐fold (or 2.1‐fold) and 2.7‐fold (or 1.6‐fold) lower than those of the control group, respectively (Figure 8f,g), which indicated a reduction in viral abundance in the lungs due to antiviral therapies. Parallel signal trends were observed in urinalysis, indicating corresponding 4.6‐ or 4.4‐fold reductions in urinary signals for peramivir‐ or zanamivir‐treated mice compared to the control group (Figure 8h). Ex vivo fluorescence imaging and biodistribution studies further corroborated these findings (Figure S29).

The prognosis of viral infection by antiviral therapies was also assessed by measuring the copy number of H1N1 virus, the levels of proinflammatory cytokines (TNF‐α and IL‐6), histological staining, and body weight. A reduction in the copy number of the H1N1 virus was observed in mice receiving various therapies as compared to the PBS‐treated group, as evidenced by 1.4 (or 1.7) and 1.6 (or 1.4)‐fold reductions in viral RNA copies of lungs (and nasal) for peramivir‐ or zanamivi‐treated mice, respectively, compared to the PBS‐treated group (Figure 8j,k). Moreover, strong positive correlations were observed between H1N1 virus copy numbers in the lungs and NIRC imaging signals (Pearson's r  =  9431, p = 0.0001, Figure 8l) or urinary fluorescence signals (Pearson's r  =  9615, *p* < 0.0001, Figure 8m). These antiviral therapies also alleviated the mice's weight loss (Figure 8i). Moreover, the TNF‐α and IL‐6 levels in mice receiving peramivir (5.7‐fold and 7.0‐fold) or zanamivir (3.2‐fold and 7.0‐fold) were significantly lower than those of the PBS‐treated groups (Figure 8e and Figure S26a,b), demonstrating that antiviral therapies could reduce the onset of inflammation. Histological studies showed that pulmonary lesions were significantly diminished in mice groups receiving peramivir or zanamivir (Figure S27). The peramivir‐ or zanamivi‐treated mice exhibited significantly lower green and red signals from the immunostaining of NA antibody and activated VLR, respectively, indicating the reduction of H1N1 virus in the lungs (Figure S28a,b). Therefore, the VLR‐based imaging and urinalysis enabled the prognostic assessment of antiviral therapies in living mice.

## 3 Conclusions

A recognized barrier in the monitoring of viral replication dynamics using optical imaging is the lack of probes that not only possess activatable signals specific to virus‐related biomarkers but also enable in vitro point‐of‐care urinalysis. While existing optical probes can detect certain post‐infection molecular events of post‐virus infection (e.g., immune activation, inflammation, ROS or RNS) [40], they have rarely been used for dynamic imaging of viral replication and localization in living animals. This challenge is tackled by our viral luminogenic reporter (VLR) that simultaneously possesses activatable signals specific to the H1N1 virus coat NA protein and high renal clearance efficiency for urinary testing.

To achieve rapid renal elimination, VLR was rationally designed as a small molecular weight (∼3 kDa) and excellent hydrophilicity (log D = −12.38). As it detects viral protease, VLR permits real‐time imaging of influenza viral replication in living cells without the need for washing steps, providing an efficient imaging platform to screen antiviral drugs. Moreover, the thermal inactivation experiments have demonstrated that VLR is superior to the qPCR assay in distinguishing between viable and nonviable H1N1 virus. VLR also achieves a limit of detection of 2.62 CCID_50_/mL in H1N1 virus detection, which was 10.4‐fold and 529.8‐fold lower than that of ICA‐qPCR and PMA‐qPCR assays. Upon i.t administration of VLR into the H1N1 virus‐infected living mice, the probe accumulated in the lungs, where it was cleaved by NA to trigger the activation of NIRC and NIRF signals. Such active sensing mechanisms enabled in vivo real‐time imaging of viral replication activity with remarkable sensitivity comparable to qPCR (Pearson's r = 0.9431, *p* < 0.0001, Figure S19a) while allowing non‐invasive and dynamic tracking. In contrast, current in vitro diagnostic methods (e.g., virus culture and qPCR) are static. Noted that the i.t. administration route enables the probe to avoid the first‐pass metabolism with good bioavailability in the lungs. More importantly, VLR‐based urinalysis enables longitudinal monitoring of the efficacy of antiviral therapies (e.g., peramivir or zanamivir) [49, 50].

In summary, we present an NA‐specific luminogenic probe for both real‐time imaging and in vitro urinalysis of the fate of the H1N1 virus in living mice. These advantages cannot be achieved by current diagnostic assays, yet they have been long and strongly desired in preclinical research and clinical practice. Thus, this study not only demonstrates strong translational potential and highlights a significant advance in addressing the critical gap in dynamic monitoring of in vivo virus replication activity but also establishes a general design principle for transforming virus‐specific probes into urinary reporters, which can be readily adapted for tracking other causative viruses.

## Author Contributions

J. H., X. M., L. L., B. J., and B. R. conceived and designed the study. B. R., J. C., and W. X. performed the probe synthesis and computer calculation. B. R., C. L., and M. H. performed in vitro experiments, in vivo experiments, and histology experiments. B. R., M. H., C. L., W. X., X. M., L. L., J. Q., B. J., and J. H. analyzed the data. B. R., C. L., and J. H. drafted the manuscript. All authors contributed to the writing of this article.

## Conflicts of Interest

The authors declare no conflicts of interest.

## Supporting information




**Supporting File**: advs76669‐sup‐0001‐SuppMat.pdf.

## Data Availability

The data that supports the findings of this study are available in the supplementary material of this article.
