# Supplementary material for: Renal Clearable Luminogenic Reporter for Ultrasensitive Influenza Virus Imaging and Efficient Antiviral Therapies Monitoring in Living Mice
Source: Adv Sci (Weinh). 2026 Jul 17:e76669. Online ahead of print. doi: 10.1002/advs.76669 (PMC13379219; doi:10.1002/advs.76669)
Supplement: Supplementary file 1 — Supporting File: advs76669‐sup‐0001‐SuppMat.pdf. [file ADVS-9999-e76669-s001.pdf]

Supplementary information

Renal Clearable Luminogenic Reporter for Ultrasensitive Influenza Virus Imaging and Efficient Antiviral Therapies Monitoring in Living Mice

Bankang Ruan[1,2,#], Chudan Liang[3,4,#], Meilin Hu[5,6,#], Weiping Xu[7,#], Jingjing Che[1], Jun Qian[4,8,9], Bingcai Jiang[2]*, Linna Liu[3]*, Xiancai Ma[5,6]*, Jiaguo Huang[1]*

[1]State Key Laboratory of Anti-Infective Drug Discovery and Development, School of Pharmaceutical Sciences, Sun Yat-sen University, Guangzhou, China

[2]Department of Ophthalmology, Guizhou Provincial People's Hospital, Guizhou, China

[3]Guangzhou Key Laboratory of Clinical Pathogen Research for Infectious Diseases, Institute of Infectious Disease, Guangzhou Eighth People's Hospital, Guangzhou Medical University, Guangzhou, China

[4]South China Center for Biosafety, Sun Yat-sen University, Guangzhou, China

[5]Institute of Human Virology Zhongshan School of Medicine, Sun Yat-sen University, Guangzhou, China

[6]Guangzhou National Laboratory, Guangzhou International Bio-Island, Guangzhou, China

[7]State Key Laboratory of Structural Chemistry, Fujian Institute of Research on the Structure of Matter, Chinese Academy of Sciences, Fuzhou, China

[8]School of Public Health (Shenzhen), Shenzhen Campus of Sun Yat-sen University, Shenzhen, China

[9]Shenzhen Key Laboratory of Pathogenic Microbes and Biosafety, Shenzhen, China

[#]These authors contributed equally: Bankang Ruan, Chudan Liang, Meilin Hu, Weiping Xu.

*E-mail: jiangbingcai@gz5055.com; liulinna7@126.com; ma_xiancai@gzlab.ac.cn; huangjg36@mail.sysu.edu.cn

Methods

General Information

All chemicals were purchased from Shanghai Aladdin Bio-Chem Technology Co. Ltd., Shanghai Bide Pharmatech Ltd, Energy Chemical Co. Ltd., Shanghai Macklin Biochemical Co. Ltd. Guangzhou Biolight Biotechnology Co., Ltd. unless otherwise stated. ALT and AST activity assay kit, creatinine and urea assay kit were purchased from Sigma-Aldrich. PCR reagents were purchased from GENCEFE Biotech (Wuxi, China), Cell Culture Reagents were purchased from AoRuiCell Biotechnology (Shanghai) Co., Ltd. Silica gel (200-300 mesh) was used for column chromatography. TLC was conducted on silica gel HSGF254 glass plates. UV-Vis absorption and fluorescence spectra were measure on a Shimadzu UV2600 spectrometer and HORIBA Fluoromax-4 fluorescence spectrophotometer, respectively. Chemiluminescence was recorded on spectramax i3x (Molecular Devices, USA). HPLC was performed on a Shimadzu LC-16P system equipped with an Ascentis C18 HPLC Column (25 cm × 21.2 mm, 5 μm) using gradient elution. Proton-nuclear magnetic resonance (^{1}H NMR) was conducted with a Bruker 400 MHz NMR instrument. Chemical shifts are reported in ppm relative to residual protic solvent resonances. Mestre Nova LITE v12.0.1.20560 software (Mestre lab Research S.L.) was used to analyze the NMR spectra. All the NMR spectra and Mass spectra are provided in Supplementary Figs. 30–56. Electrospray ionization-mass spectrometry (ESI-MS) spectra were obtained on a Waters SQD2 with Waters H-Class UPLC equipped with a standard ESI source. an IVIS imaging system from RWD Life science (MOIS HT Small Animal In Vivo Optical Imaging System, China). Fluorescence images *in vitro* were acquired using the EVOS M5000 inverted fluorescence microscope (Invitrogen, Thermo Fisher Scientific, USA) and the Nikon AX NSPARC confocal microscope (Nikon Instruments Inc, Japan). All the virus nucleic acid testing was conducted in the QuantStudio™ 5 Real-Time PCR System (Applied Biosystems, Thermo Fisher Scientific, USA). Mouse anesthesia was conducted at Microinjection system for anesthesia localization in small animals (ZS-MV-HR, Beijing Zhongshi Dichuang Technology Development Co., Ltd.). Tissues were cut into sections using a cryostat (Leica, Germany) and examined using a Nikon ECLIPSE 80i microscope (Nikon Instruments Inc, USA). Confocal microscopy images of tissue sections were acquired on a

FV3000 confocal laser scanning microscope (Olympus Corporation, Japan). (PerkinElmer, Inc, USA). EdU Imaging Kits (K2240, APExBIO, Houston, USA). The in vivo imaging was performed by a luminescent imaging system ((Shanghai United Digital Biotech. Co. Ltd., NIR-II-ST) and (PerkinElmer, Inc, USA)). Blood and urine samples were collected using heparinized capillary tubes and metabolic cages respectively. MALDI-TOF analysis was performed on a Bruker ultraflex TOF/TOF instrument.

Synthesis of compound 1：Compound **1** was prepared according to literature procedure.[31] To a solution of 3,5-dimethoxybenzyl bromide (916 mg, 4 mmol) and compound **9** (800 mg, 5 mmol) in dry THF (20 mL) was added sodium hydride (240 mg, 10 mmol) at 0 °C, then the reaction mixture was stirred at room temperature overnight under an Argon (Ar) atmosphere. The reaction mixture was quenched with H_2O (50 mL) and extracted with ethyl acetate (EtOAc) for three times. The combined organic layer was concentrated under reduced pressure. The purification using column chromatography on silica gel was performed to afford the intermediate as colorless oil (1.18 g, yield of 95%). A solution of the intermediate (620 mg, 2 mmol) and PCC (473 mg, 2.2 mmol) in Dichloromethane (DCM) (20 mL) was stirred at room temperature overnight under an Ar atmosphere. The reaction mixture was quenched with H_2O (50 mL) and extracted with DCM for three times. The combined organic layer was concentrated under reduced pressure. The purification using column chromatography on silica gel was performed to afford the compound **1** as yellowish oil (604 mg, yield of 98%). ^{1}H NMR (400 MHz, $CDCl_3$) δ 6.45 (d, *J* = 2.4 Hz, 2H), 6.36 (t, *J* = 2.0 Hz, 1H), 4.43 (s, 2H), 3.78 (s, 6H), 3.49 (s, 2H), 1.28 (s, 6H), 1.24 (s, 9H). ^{13}C NMR (101 MHz, $CDCl_3$) δ 170.58, 160.77, 141.22, 105.07, 99.44, 82.51, 77.81, 73.03, 55.25, 41.17, 37.00, 28.61, 23.47, 20.94. ESI-MS (m/z): $[M+Na]^+$ Calcd for $C_{18}H_{28}O_4$:331.19; Found 331.42.

Synthesis of compound 2：Compound **2** was prepared according to literature procedure.[31] To a solution of compound **1** (308 mg, 1 mmol) in dry THF (10 mL) was added lithium diisopropylammonium (2 M in THF, 1.5 mL) at -78 °C for 2 h under an Ar atmosphere. The reaction mixture was quenched with cold H_2O (15 mL) and extracted with EtOAc for three times. The combined organic layer was concentrated under reduced pressure. The purification using column chromatography on silica gel was performed to afford the compound 2 as yellowish oil (227 mg, yield of 74%). ^{1}H NMR (400 MHz,

$CDCl_3$) δ 6.71 (d, *J* = 2.4 Hz, 2H), 6.37 (t, *J* = 2.4 Hz, 1H), 4.96 (s, 1H), 3.90 (d, *J* = 8.8 Hz, 1H), 3.78 (s, 6H), 3.69 (d, *J* = 8.8 Hz, 1H), 1.92 (s, 1H), 1.39 (s, 3H), 1.19 (s, 3H), 0.92 (s, 9H). ^{13}C NMR (101 MHz, $CDCl_3$) δ 160.30, 149.86, 137.97, 125.29, 107.95, 100.50, 83.12, 55.34, 47.15, 32.42, 27.43. ESI-MS (m/z): $[M+Na]^+$ Calcd for $C_{18}H_{28}O_4$:331.19; Found 331.50.

Synthesis of compound 3： Compound **3** was prepared according to literature procedure.[31] To a solution of compound **2** (308 mg, 1 mmol) in dry DCM (10 mL) was added silver trifluoromethanesulfonate (258 mg, 1 mmol) and stirred for 30 minutes, followed by the addition of 1,1-dichlorodimethyl ether and stirred for 2 h under an Ar atmosphere. The reaction mixture was quenched with H_2O (50 mL) and extracted with DCM for three times. The combined organic layer was concentrated under reduced pressure. The purification using column chromatography on silica gel was performed to afford the compound **3** as white solid (210 mg, yield of 68%). ^1H NMR (400 MHz, $CDCl_3$) δ 10.27 (s, 1H), 7.15 (d, *J* = 2.4 Hz, 1H), 6.41 (d, *J* = 2.4 Hz, 1H), 5.39 (s, 1H), 5.09 (s, 1H), 3.90 (d, *J* = 4.8 Hz, 6H), 3.68 (dd, *J* = 20.4, 8.8 Hz, 2H), 1.37 (s, 3H), 1.26 (s, 3H), 0.86 (s, 9H). ^{13}C NMR (101 MHz, $CDCl_3$) δ 189.98, 165.37, 165.24, 146.31, 117.96, 106.93, 97.04, 88.96, 88.59, 79.69, 55.98, 55.63, 49.06, 40.75, 26.44, 23.83. ESI-MS (m/z): $[M+Na]^+$ Calcd for $C_{19}H_{28}O_5$:359.19; Found 359.50.

Synthesis of compound 4： Compound **4** was prepared according to literature procedure.[31] A solution of compound **3** (336 mg, 1 mmol), BBr_3 (500 mg, 2 mmol) and dry DCM (5 mL) was stirred at 0 ℃ for 2 h under an Ar atmosphere. The reaction mixture was quenched with H_2O (50 mL) and extracted with EtOAc for three times. The combined organic layer was concentrated under reduced pressure. The purification using column chromatography on silica gel was performed to afford the compound **4** as yellow solid (226 mg, yield of 78%). ^1H NMR (400 MHz, $CDCl_3$) δ 11.97 (s, 1H), 9.83 (s, 1H), 6.35 (d, *J* = 4.4 Hz, 2H), 3.91 (s, 2H), 1.35 (s, 6H), 1.07 (s, 9H). ^{13}C NMR (101 MHz, $CDCl_3$) δ 159.28, 156.72, 147.76, 138.89, 126.05, 108.17, 99.79, 83.41, 56.34, 55.62, 47.17, 32.56, 31.70, 27.03. ESI-MS (m/z): $[M+H]^+$ Calcd for $C_{17}H_{22}O_4$:291.15; Found 291.26.

Synthesis of compound 5： A solution of compound **4** (290 mg, 1 mmol), Cs_2CO_3 (325 mg, 1 mmol), 2-bromocyclohex-1-ene-1-carbaldehyde (188 mg, 1mmol) and DMF (3 mL) was stirred at room

temperature for 12 h under an Ar atmosphere. The reaction mixture was quenched with H_2O (50 mL) and extracted with EtOAc for three times. The combined organic layer was concentrated under reduced pressure. The purification using column chromatography on silica gel was performed to afford the compound **5** as yellow solid (137 mg, yield of 36%). ^{1}H NMR (400 MHz, $CDCl_3$) δ 10.35 (s, 1H), 6.72 (s, 1H), 6.63 (d, *J* = 2.0 Hz, 1H), 6.59 (s, 1H), 3.91 (s, 1H), 2.62 – 2.56 (m, 2H), 2.47 (t, *J* = 5.6 Hz, 2H), 1.74 (dd, *J* = 6.7, 3.2 Hz, 4H), 1.57 (s, 6H), 1.06 (s, 9H). ^{13}C NMR (101 MHz, $CDCl_3$) δ 200.80, 166.52, 160.75, 141.15, 105.14, 99.45, 83.89, 78.22, 73.01, 55.27, 50.07, 41.15, 37.05, 30.48, 25.66, 23.56, 23.30. ESI-MS (m/z): $[M+H]^+$ Calcd for $C_{24}H_{28}O_4$:380.19; Found 380.35.

Synthesis of compound 6：A solution of compound **5** (380 mg, 1 mmol), EtOH (10 ml), AcONa (82 mg, 1 mmol) and compound **11** (1 mmol) was stirred at 60 ℃ for 2 h under an Ar atmosphere. The reaction mixture was quenched with H_2O (50 mL) and extracted with DCM for three times. The combined organic layer was concentrated under reduced pressure. The purification using column chromatography on silica gel was performed to afford compound **6**. ^{1}H NMR (500 MHz, DMSO) δ 8.60 (d, *J* = 15 Hz, 1H), 7.78 (s, 1H), 7.69 (s, 1H), 7.60 – 7.53 (m, 2H), 7.49 (dd, *J* = 19.5, 8.0 Hz, 1H), 6.99 (s, 1H), 6.91 (t, *J* = 8.5 Hz, 1H), 6.53 (d, *J* = 15 Hz, 1H), 2.76 (s, 2H), 2.71 (s, 2H), 1.87 (s, 2H), 1.79 (s, 6H), 1.29 (d, *J* = 17.5 Hz, 2H), 0.90 (d, *J* = 12 Hz, 8H), 0.88 (s, 17H). ESI-MS (m/z): $[M+H]^+$ Calcd for $C_{39}H_{47}N_4O_3^+$:619.36; Found 620.37.

Synthesis of compound 7：A solution of compound **6** (62 mg, 0.1mmol), compound **13** (41 mg, 0.1 mmol), K_2CO_3 (27 mg, 0.2mmol), Cs_2CO_3(33 mg, 0.1 mmol) and DCM (3mL) was stirred at room temperature for 2 h under an Ar atmosphere. The reaction mixture was quenched with H_2O (50 mL) and extracted with DCM for three times. The combined organic layer was concentrated under reduced pressure to afford the intermediate. It could be directly put into the next step of reaction without purification. A solution of the intermediate (0.1 mmol), NaOMe (27 mg, 0.5mmol) and MeOH (3 mL) was stirred at room temperature for 1 h under an Ar atmosphere. The reaction mixture was quenched with NH_4Cl (50 mL) and extracted with DCM for three times. Compound **7** was afforded by reduced pressure concentration. ^{1}H NMR (500 MHz, DMSO) δ 8.62 (d, *J* = 14.5 Hz, 1H), 8.33 (d, *J* = 8.5 Hz, 1H), 7.81 (d, *J* = 7.5 Hz, 1H), 7.71 (d, *J* = 7.5 Hz, 1H), 7.60 (s, 1H), 7.47 (s, 1H), 7.02 (s, 1H), 6.97 (d,

J = 7.5 Hz, 1H), 6.61 – 6.50 (m, 1H), 4.00 – 3.91 (m, 1H), 3.83 (d, J = 10.5 Hz, 1H), 3.66 (d, J = 10.5 Hz, 2H), 3.55 (d, J = 8.5 Hz, 3H), 3.35 (d, J = 10.5 Hz, 2H), 3.25 (d, J = 9.5 Hz, 2H), 2.95 (s, 1H), 2.81 – 2.70 (m, 6H), 2.15 – 2.04 (m, 2H), 1.97 (s, 6H), 1.80 (s, 12H), 0.84 (d, J = 39.5 Hz, 17H). ^{13}C NMR (126 MHz, DMSO) δ 169.17, 169.08, 161.06, 154.74, 142.90, 142.17, 134.68, 129.72, 129.20, 127.05, 123.04, 114.90, 114.17, 113.23, 104.19, 102.45, 50.37, 32.85, 28.76, 27.74, 24.08, 20.57, 14.37, 0.58. ESI-MS (m/z): [M+H]$^+$ Calcd for $C_{50}H_{64}N_5O_{11}^+$:910.45; Found 911.54.

Synthesis of VLR： A solution of compound **7** (0.01 mmol) and Propynyl HPβCD (45 mg, 0.04 mmol solubled in 500 μL water) was dissolved in THF (5 mL). Then copper sulfate pentahydrate (2 mg, 0.008 mmol) and sodium ascorbate (2.5 mg, 0.01 mmol) were added to the mixture solution with stirring at 0 °C for 5 h under argon atmosphere. After completion, THF was removed by low pressure concentration, and then extracted with dichloromethane (3 x 30 mL) and pure water (3 x 30 mL). Compound **VLR** was afforded by reduced pressure concentration. ^{1}H NMR (500 MHz, DMSO) δ 8.62 (d, J = 14.5 Hz, 1H), 8.31 (d, J = 8.0 Hz, 1H), 7.80 (d, J = 7.5 Hz, 1H), 7.70 (d, J = 8 Hz, 1H), 7.58 (d, J = 15.5 Hz, 2H), 7.47 (t, J = 7.5 Hz, 1H), 6.99 (d, J = 23.5 Hz, 1H), 6.60 – 6.51 (m, 1H), 3.82 (d, J = 10.5 Hz, 7H), 3.66 (d, J = 9.5 Hz, 20H), 3.35 (d, J = 10 Hz, 6H), 3.28 – 3.20 (m, 3H), 2.94 (s, 1H), 2.72 (s, 6H), 1.97 (s, 7H), 1.80 (s, 20H), 1.08 (s, 6H), 0.88 (s, 18H). ^{13}C NMR (126 MHz, DMSO) δ 180.15, 172.48, 168.45, 168.41, 168.38, 157.82, 156.58, 156.40, 152.07, 148.90, 142.86, 141.50, 134.84, 133.98, 131.96, 129.14, 128.90, 128.63, 127.04, 126.19, 123.42, 120.38, 120.20, 115.60, 114.12, 112.57, 108.51, 81.84, 73.41, 70.25, 69.29, 63.96, 63.67, 55.48, 51.69, 49.00, 45.95, 44.23, 42.44, 40.30, 39.80, 39.47, 37.80, 30.13, 28.72, 28.32, 27.97, 27.18, 26.64, 26.39, 25.82, 25.49, 23.97, 21.46. MALDI-TOF MS found: ~3500.

Synthesis of compound 8： Compound **8** was prepared according to literature procedure.[31] A solution of compound 1-Methoxy-1-(trimethylsilyloxy)-2-methyl-1-propene (870 mg, 5 mmol), trimethylacetyl chloride (660 mg, 5.5 mmol), $TiCl_4$ (1.13g, 6 mmol) and dry DCM (20 mL) was stirred at 0 °C under an Ar atmosphere. The reaction mixture was quenched with H_2O (50 mL) and extracted with DCM for three times. The combined organic layer was concentrated under reduced pressure. The purification using column chromatography on silica gel was performed to afford the compound **8** (178 mg, yield of

96%). ^{1}H NMR (400 MHz, $CDCl_3$) δ 3.72 (s, 3H), 1.39 (s, 6H), 1.19 (s, 9H). ^{13}C NMR (101 MHz, $CDCl_3$) δ 52.51, 50.15, 43.50, 26.44, 25.07, 21.60, 19.95. ESI-MS (m/z): $[M+H]^+$ Calcd for $C_{10}H_{18}O_3$:186.13; Found 225.25.

Synthesis of compound 9： Compound **9** was prepared according to literature procedure.[31] A solution of compound **8** (744 mg, 4 mmol) and $LiAlH_4$ (444 mg, 12 mmol) in DCM (20 mL) was stirred at room temperature overnight under an Ar atmosphere. The reaction mixture was quenched with H_2O (50 mL) and extracted with DCM for three times. The combined organic layer was concentrated under reduced pressure to afford compound **9** as colorless oil (627 mg, yield of 98%). It could be directly put into the next step of reaction without purification. ^{1}H NMR (400 MHz, $CDCl_3$) δ 3.52 (d, *J* = 10.8Hz, 1H), 3.43 (d, *J* = 10.4 Hz, 1H), 3.30 (s, 1H), 2.99 (s, 2H), 1.04 (s, 9H), 1.04 (s, 3H), 1.03 (s, 3H). ^{13}C NMR (101 MHz, $CDCl_3$) δ 85.41, 74.70, 40.44, 37.28, 28.48, 26.04, 25.51, 20.68. ESI-MS (m/z): $[M+H]^+$ Calcd for $C_9H_{20}O_2$:160.15; Found 183.15.

Synthesis of Propynyl-HPβCD: Propynyl-HPβCD was prepared according to literature procedure.[32] A mixture of hydroxypropyl-β-cyclodextrin (1.54 g, 1 mmol), 3-bromo propylene (0.72 g, 6 mmol), sodium iodide (0.3 g, 2 mmol) and sodium hydride (0.24 g, 10 mmol) in dry *N, N*-dimethylformamide (20 mL) was stirred at room temperature for 20 h. Upon completionthe mixture was concentrated under reduced pressure, the crude product was separated by column chromatography, and dialyzed at room temperature for 24 hours, then freeze-dried to afford **Propynyl-HPβCD** as yellowish solid (1.3 g, yield 87%). ^{1}H NMR (400 MHz, D_2O) *δ* 5.16 (m, 7H), 4.01–3.65 (m, 73H), 2.72 (s, 1H), 1.16 (m, 16H).

Synthesis of compound 11： A solution of 1,4-dibromohexane (244 mg, 1 mmol) and 2,3,3-Trimethylindolenine (160 mg, 1 mmol) was stirred at room temperature for 6 h under an Ar atmosphere. The mixture was added to the diethyl ether (50 mL), the solid was filtered and washed with diethyl ether for three times to afford compound **10**. A solution of compound **10**, sodium azide (260 mg, 4 mmol) and anhydrous acetonitrile (10 mL) was stirred at 90 °C for 6 h under an Ar atmosphere. The reaction mixture was quenched with H_2O (50 mL) and extracted with DCM for three times. The combined organic layer was concentrated under reduced pressure to afford compound **11** (627 mg,

yield of 98%). It could be directly put into the next step of reaction without purification. ^{1}H NMR (400 MHz, DMSO) δ 8.12 – 7.94 (m, 1H), 7.91 – 7.77 (m, 1H), 7.69 – 7.56 (m, 2H), 4.55 (t, J = 6.8 Hz, 2H), 3.63 (t, J = 6.0 Hz, 2H), 2.89 (s, 3H), 2.20 – 1.79 (m, 4H), 1.56 (s, 6H). ESI-MS (m/z): $[M+H]^+$ Calcd for $C_{15}H_{21}N_4^+$:257.17; Found 258.25.

Synthesis of compound 13： A solution of N-acetylneuraminic acid methyl ester (323 mg, 1mmol), pyridine (1 mL) and acetic anhydride (3 mL) was stirred at 0 °C for 6 h under an Ar atmosphere. The mixture was diluted with EtOAc and washed with HCl. The organic layer was separated, dried over Na_2SO_4, filtered and the solvent was evaporated under reduced pressure to afford compound **12.** A solution of compound **12** (533 mg, 1mmol), acetic acid (10 mL) and HBr (2 mmol) was stirred at 0 °C for 6 h under an Ar atmosphere. The reaction mixture was quenched with H_2O (50 mL) and extracted with EtOAc for three times. The combined organic layer was concentrated under reduced pressure. The compound **4** was reacted without further purification. ^{1}H NMR (400 MHz, $CDCl_3$) δ 5.62 (dd, J = 7.6, 2.8 Hz, 1H), 5.53 – 5.47 (m, 1H), 5.36 (d, J = 4.4 Hz, 6H), 4.64 – 4.57 (m, 3H), 4.45 – 4.38 (m, 4H), 4.23 – 4.08 (m, 5H), 3.82 – 3.78 (m, 3H), 2.14 (s, 6H), 2.08 (s, 6H), 2.06 (s, 3H), 2.03 (d, J = 2.4 Hz, 3H). ESI-MS (m/z): $[M+H]^+$ Calcd for $C_{20}H_{28}BrNO_{12}$:553.07; Found 554.65.

Preparation of stock solution: VLR (10 μM) was dissolved in PBS (10 mM, pH 7.4) to obtain a stock solution after filtration by using syringe filter (0.22 μm). H_2O_2 and ClO^- stock solution was prepared by directly diluting H_2O_2 and NaClO. •OH was generated by Fenton reaction between H_2O_2 and $Fe(ClO_4)_2$. Stock solution of NaCl, KCl, $MgSO_4$, glutathione, β-galcatosidase, gamma-glutamyl transferase and *N*-acetyl-β-D-glucosaminidase were prepared with distilled water.

Absorption, chemiluminescence and fluorescence spectra: The **VLR** solution (20 μM) was incubated with NA (40 mU) in PBS (10 mM, pH 7.4). Absorption and fluorescence spectra of the solution were measured on UV-VIS and fluorescence spectrophotometer. NIR fluorescence images were acquired using the IVIS spectrum imaging system with the excitation at 675 ± 10 nm, the emission at 720 ± 10 nm and the acquisition time of 0.1 s.

Selectivity studies: The **VLR** solution (10 μM) was incubated ROS (40 μM), metal ions (40 μM) and

enzymes including glutathione (1.0 U), β-galcatosidase (1.0 U), gamma-glutamyl transferase (1.0 U) and *N*-acetyl-β-D-glucosaminidase (40 mU) in HEPES buffer (50 mM, pH 7.4) or PBS (10 mM, pH 7.4) at 37 °C for 120 min. Fluorescence and chemiluminescence enhancements of **VLR** were measured after incubation.

The limit of detection (LOD): Chemiluminescence intensities of **VLR** (10 μM) were determined after addition of different concentrations of NA. The LOD was calculated based on the equation: LOD = 3σ/k, where σ is the standard deviation of emission intensity of blank, and k is the slope of the plot of emission intensities against the concentration of NA.

Chemiluminescence kinetic profiles: NA (40 μM) was added into the **VLR** solution (10 μM) in PBS (10 mM, pH = 7.4). Chemiluminescence intensities were continuously acquired and plotted as a function of time.

Enzymatic kinetics assays: VLR (10, 20, 30, 60 and 90 μM) were incubated with NA (10 mU) at 37 °C. The cleavage compound was quantified using HPLC after incubation. The initial reaction velocity (μM min^{-1}) was calculated, plotted against the concentration of **VLR** and fitted to a Michaelis-Menten curve. The kinetic parameters were calculated by the following equation: V=Vmax[S] / (Km + [S]), Where V is the initial velocity, and [S] is the substrate concentration.

Thermal stability: CL-1 or **CL-2** (10 μM) was dissolved in FBS solution and placed in 13 tubes with the same concentration and stored at room temperature in dark. Chemiluminescence intensities were recorded for each sample after the addition of KO_2 (40 μM) at different storage time points.

pH Stability: VLR solution (20 μM) was incubated in different buffer solution with a pH range from 4.0 to 8.5 at 37 °C for 3 h. Fluorescence intensity of **VLR** was measured on IVIS spectrum imaging system after incubation.

Cell culture: The Madin-Darby canine kidney (NBL-2) (MDCK) and human non-small cell lung cancer (A549) cell lines was cultured in high-glucose Dulbecco’s Modified Eagle Medium (DMEM) (BasalMedia, L120KJ) supplemented with 10% fetal bovine serum (FBS) (ExCell Bio, FSP500), 100 U/mL penicillin and 100 μg/mL streptomycin (GIBCO, 15140122) at 37 °C with 5% CO_2.

Virus: The influenza A virus (IAV) H1N1 strain (A/Puerto Rico/8/1934) was preserved in the laboratory, while the H3N2 strain and influenza B virus (IBV) (Victoria lineage) were isolated from clinical samples and preserved in the laboratory. All experiments related to virus cell infection were performed in the biosafety level-2 (BSL-2) laboratory of the Guangzhou Eighth People's Hospital, Guangzhou Medical University.

Cell viability assay: The assessment of the cytocompatibility of **VLR** was conducted by Cell Counting Kit-8 (CCK-8) (Beyotime, C0037) method according to the manufacturer's instructions. The MDCK cells were incubated with increasing concentrations of **VLR** (0.5 to 250 μM) for 24 h. The CCK-8 assay was then evaluated using Multimode Microplate Reader (Agilent, BioTek, SH1M-SN, USA), measuring absorbance at 500 nm, and the resultant data were interpreted as the proportion of viable cells.

Cellular imaging after H1N1 virus infection: The virus and the infected cells seeding in 12-well plates (Cellvis, P12-1.5P) were subjected to incubate with **VLR** for approximately 5~10 min to achieve fluorescence imaging. The multiplicity of infection (MOI) of viruses used in this study was 0.01. The cell imaging system (Invitrogen, Thermo Fisher Scientific, EVOS M5000, USA) and the confocal microscope (Nikon Instruments Inc, AX NSPARC, Japan) were used, with measurements being conducted at the optimal wavelength. The Dio (3,3'-dioctadecyloxacarbocyanine perchlorate) (Beyotime, C1038) was used in the study to trace the cell membrane in living cells. All the intensity of fluorescence was calculated using Image J software.

Virus RNA extraction and RT-qPCR: The filtered virus and infected cells were harvested to extract the viral RNA. The extraction process was conducted using TRIzol (Invitrogen, 15596026CN) by the Ultrapure RNA Kit (CWBIO, CW0581M), and was inverted to complementary DNA (cDNA) immediately using the Evo M-MLV Reverse Transcription Kit (Accurate Biology, AG11705). The quantitative real-time polymerase chain reaction (qPCR) was performed using the MagicSYBR Mixture (CWBIO, CW3008H) in the QuantStudio™ 5 Real-Time PCR System (Applied Biosystems, Thermo Fisher Scientific, USA). The forward primer of 5′-CTTCTAACCGAGGTCGAAACGTA-3′, and the reverse primer of 5′- GGTGACAGGATTGGTCTTGTCTTTA-3′ were used in the study. The resultant data was obtained by the QuantStudio Design & Analysis software, and the viral copy

numbers were measured according to the previously established protocol based on the standard equation that was constructed using viral DNA standards.

Virus inactivation and distinguish from viable and nonviable virus: The thermal treatment was used to inactivate the virus at temperatures of 56 °C, 60 °C, 70 °C and 100 °C for 30 min in the thermostat water bath. The inactivated virus was then used to infect the MDCK cells for 1 h, subsequent to the replacement of the maintenance culture medium. The infected cells were incubated with the **VLR** following a 24-hour cultivation. The cytopathic effect (CPE) was observed after a 72-hour cultivation under the microscope to assess the toxicity of the virus.

Quantification of intact infectious virus: The integrated cell absorption quantitative polymerase chain reaction (ICA-qPCR), and propidium monoazide quantitative polymerase chain reaction (PMA-qPCR) assays were used to quantify the infectious titer of virus, as a comparison with **VLR** for intact infectious virus detection. The limit of detection (LOD) was analyzed using Probit regression analysis, a method that can be used for sensitivity analysis, by Medclac software. The ICA-qPCR and PMA-qPCR methods were conducted according to the protocol that had been established in the preceding research.

Screening of antiviral drugs in cells: A range of antiviral medications were used in the study, such as N-acetylneuraminic acid (NA) and the RNA-dependent RNA polymerase (RdRp), amongst others. The following antiviral agents were included in the study: tubeimoside (TUB), rapamycin (RAPA), ursodeoxycholic acid (UDCA), remdesivir (RDV), chloroquine (CQ), baloxavir (BXM), prochlorperazine (PCZ), ribavirin (RBV), nitazoxanide (NTZ), favipiravir (T-750), zanamivir (ZNM), oseltamivir (OSV), and peramivir (PRM). All the medications procured from Sigma-Aldrich and TargetMol Chemicals Inc. were meticulously diluted according to the guidelines using dimethyl sulfoxide (DMSO) and PBS. The MDCK cells were subjected to treatment with the aforementioned agents for 1 h prior to virus infection, following the replacement of the maintenance culture medium containing the agents. Then the drug treated- and infected- cells were incubated with the **VLR** after a 12-hour cultivation.

Quantification of H1N1 Viral Burden in Lung Tissues by RT-qPCR: Approximately 30 mg of lung tissue was homogenized, and total RNA was extracted using the Universal RNA Purification Kit (EZB-RN4) according to the manufacturer's instructions. H1N1 viral RNA levels were quantified by RT-qPCR targeting the matrix (M) gene using the following primers and probe: HM-F (5'-GACCRATCCTGTCACCTCTGAC-3'), HM-R (5'-GGGCATTYTGGACAAAKCGTCTACG-3'), and HM-P (5'-FAM-TGCAGTCCTCGCTCACTGGGCACG-BHQ1-3'). Viral RNA copy numbers were calculated from standard curves generated by serial dilutions of H1N1 viral RNA standards.

Histological examination: All tissues were fixed with 4% paraformaldehyde (PFA), dehydrated in ethanol solution, embedded in paraffin, and cut into 10 μm thick sections for H&E staining. Paraffin was removed by washing with xylene, and sections were incubated with hematoxylin for 4 min and eosin for 2 min, followed by washing with distilled water. Stained sections were examined using the FL Automated Imaging System + EVOS® on-stage incubator. Mouse lung was snap frozen in OCT (Tissue-Tek) and stored at -20 °C until sectioning. Frozen lung sections were first baked in an oven at 37°C for 10–20 min to remove moisture, followed by fixation in a fixative solution for 30 min; afterward, the sections were washed three times with phosphate-buffered saline (PBS, pH 7.4) on a decolorizing shaker, with each wash lasting 5 min. For antigen retrieval, the process was conducted under conditions detailed in the table above, during which excessive evaporation of the buffer must be prevented to avoid section desiccation; once retrieval was completed, the sections were allowed to cool naturally and then washed three times with PBS (pH 7.4) on a decolorizing shaker (5 min per wash). After gentle drying, the tissue sections were circled with a histochemical pen to define the reaction area, and blocking was performed by adding bovine serum albumin (BSA) solution—while 3% BSA was applied for influenza A H1N1 virus neuraminidase antibody (bs-16642r, Bioss, 1:200)—for 30 min at room temperature. Next, diluted primary antibody was added dropwise to the sections, which were then placed flat in a humidified chamber and incubated overnight at 4°C. Following primary antibody incubation, the slides were washed three times with PBS (pH 7.4) on a decolorizing shaker (5 min per wash), after which the corresponding secondary antibody (Alexa Fluor 488-labeled goat anti-rabbit IgG (GB25303, Servicebio, 1:400)) was added and the sections were incubated at room

temperature for 50 min in the dark. Prior to DAPI nuclear counterstaining, the slides were washed three times with PBS (pH 7.4) on a decolorizing shaker (5 min per wash); DAPI staining solution was then added, and incubation was carried out at room temperature for 10 min in the dark. Subsequent to DAPI staining, the slides were washed three times with PBS (pH 7.4) on a decolorizing shaker (5 min per wash) to prepare for quenching of tissue autofluorescence, which was achieved by treating the sections with Solution B for 5 min followed by rinsing under running water for 10 min. Finally, the sections were mounted using an anti-fluorescence quenching mounting medium to preserve signal stability, and fluorescence images were acquired using the following excitation (Ex) and emission (Em) wavelengths: DAPI (Ex: 330–380 nm, Em: 420 nm), 488 nm fluorophore (Ex: 465–495 nm, Em: 515–555 nm), CY3 (Ex: 510–560 nm, Em: 590 nm), and CY5 (Ex: 608–648 nm, Em: 672–712 nm).

Pharmacokinetic studies: Ethics approval statement: Guangzhou National Laboratory Animal Center approved the mouse experiments in this study (Approval Number: GZLAB-AUCP-2025-06-A03). 8-weeks male mice were selected to be injected with **VLR** (2.5 μmol kg^{-1} body weight) via intratracheal injection. Before injection, the tail of the mice was cut and blood was drawn with heparin capillary. After injection of **VLR**, blood was taken at 1, 4, 9, 16, 30, 60, 120, 240, 720 and 1440 min respectively. The collected blood samples were stored in an ice box to prevent coagulation, and then centrifuged at 4500 r. p. m. for 15 minutes. **VLR** in the blood were quantified using HPLC and plotted as a function of time to calculate elimination half-life value ($t_{1/2\beta}$).

Renal clearance studies: Male mice were i.t. injected with **VLR** (2.5 μmol kg^{-1} body weight) and **CyECD** (2.5 μmol kg^{-1} body weight) and placed in metabolic cages. Feces were collected for 7 d post-injection time and urine was collected at 24 h post-injection time, diluted in PBS and centrifuged at 4500 r. p. m. for 10 min and filtered by 0.22 μm syringe filter. **VLR** and **CyECD** in the urine were quantified using HPLC.

Biocompatibility studies: Major organs including heart, liver, spleen, lung and kidneys were collected from mice after 24 h injection of PBS, **VLR** and **CyECD** (2.5 μmol kg^{-1} body weight), then placed into 4% paraformaldehyde for histological examination. Serum ALT, AST, BUN and sCr were measured using commercially assay kits.

Establishment of an artificial NA-positive mice model and real time in vivo imaging: Mice were i.t. injected with commercially available recombinant NA (0.01 mg or 0.05 mg per mouse). The control mice were treated with PBS. Real-time fluorescence and chemiluminescence imaging were conducted at 1 min and then every 10 min for 40 min after i.t. injection of VLR (2.5 μmoL kg-1 body weight). Fluorescence images were acquired using the IVIS spectrum imaging system with the excitation at 680 ± 10 nm, the emission at 720 ± 10 nm and the acquisition time of 1 s. Chemiluminescence images were acquired under bioluminescence mode with open filter and the acquisition time of 60 s. Fluorescence and chemiluminescence intensities of lung and kidney in living mice were analyzed by the ROI analysis using the Living Image 4.3 Software. Mice were euthanized after imaging at 40 min post-treatment of VLR. Major organs were collected and placed into 4% paraformaldehyde for histological examination.

Establishment of H1N1 virus infected mice model and real time in vivo imaging: For the establishment of lung IAV infection, mice were injected via nasal instillation with saline or 10 plaque-forming units (PFU) of influenza virus (A/Puerto Rico/8/1934, H1N1) in 30 μL PBS at different infection time points (1, 3, 5 or 7 days). The control mice were treated with PBS. Real-time fluorescence and chemiluminescence imaging were conducted at 1 min and then every 10 min for 40 min after i.t. injection of VLR (2.5 μmoL kg-1 body weight) at 1, 3, 5 or 7 days. Fluorescence images were acquired using the IVIS spectrum imaging system with the excitation at 680 ± 10 nm, the emission at 720 ± 10 nm and the acquisition time of 1 s. Chemiluminescence images were acquired under bioluminescence mode with open filter and the acquisition time of 60 s. Fluorescence and chemiluminescence intensities of lung and kidney in living mice were analyzed by the ROI analysis using the Living Image 4.3 Software. Mice were euthanized after imaging at 40 min post-treatment of VLR. Major organs were collected and placed into 4% paraformaldehyde for histological examination.

Real-time in vivo imaging of the efficacy of antiviral therapies in living mice: Real-time chemiluminescence and fluorescence imaging was conducted at 1 d, 3 d, 5 d and 7 d viral infection after i.t. injection of **VLR** (2.5 μmol kg^{-1} body weight). H1N1 virus-infected mice received respective daily administration of antiviral medications including peramivir (PRM), zanamivir (ZNM), or PBS as

the control group. Following various therapies, VLR (2.5 μmol kg-1 body weight) was then i.t. injected to mice at 1, 3, 5, and 7-days post-infection time. Chemiluminescence images were acquired under bioluminescence mode with open filter and the acquisition time of 60 s. Fluorescence images were acquired using the IVIS spectrum imaging system with the excitation at 670 ± 10 nm, the emission at 720 ± 10 nm and the acquisition time of 0.1 s. Fluorescence and chemiluminescence intensities of lung and kidney in living mice were analyzed by the ROI analysis using the Living Image 4.3 Software and Image J. Mice were euthanized after imaging at 60 min post-injection of probes. Major organs were collected and placed into 4% paraformaldehyde for histological examination.

Urinalysis: Collected urine was measured in PBS (10 mM, pH 7.4) by UV-Vis and fluorescence spectrophotometry, imaged with the IVIS spectral imaging system, and analyzed by HPLC. In artificial NA-positive mice model, the urine of NA (0.01 mg or 0.05 mg per mouse) group and PBS-group were collected at 1, 3, 6 and 12 h, respectively. In the H1N1 virus infected mice model, the urine of different post-infection time (1, 3, 5 or 7 days) was collected after 40 min post-treatment of VLR. In the efficacy of antiviral therapies mice model, the urine of ZNM-group, PRM-group and PBS-group was collected after 40 min post-treatment of VLR at 3 d post-infection of virus.

Statistics analysis: The in vivo and ex vivo chemiluminescence and fluorescence intensities were quantified with ROI analysis using Living Image 4.3 Software. Data are mean ± standard deviation (S. D.) unless stated otherwise. Investigators were blinded to group allocation during experiments. Statistical differences between two groups were tested with a two-tailed Student's t-test and more than three groups were determined by one-way analysis of variance followed by Tukey's post hoc test. For all tests, P values less than 0.05 were considered statistically significant. *$P < 0.05$, **$P < 0.01$, ***$P < 0.001$ and ****$P < 0.0001$. All statistical calculations were performed using Graph Pad Prism 6.0, including assumptions of tests used.

1. Supplementary figures and Tables

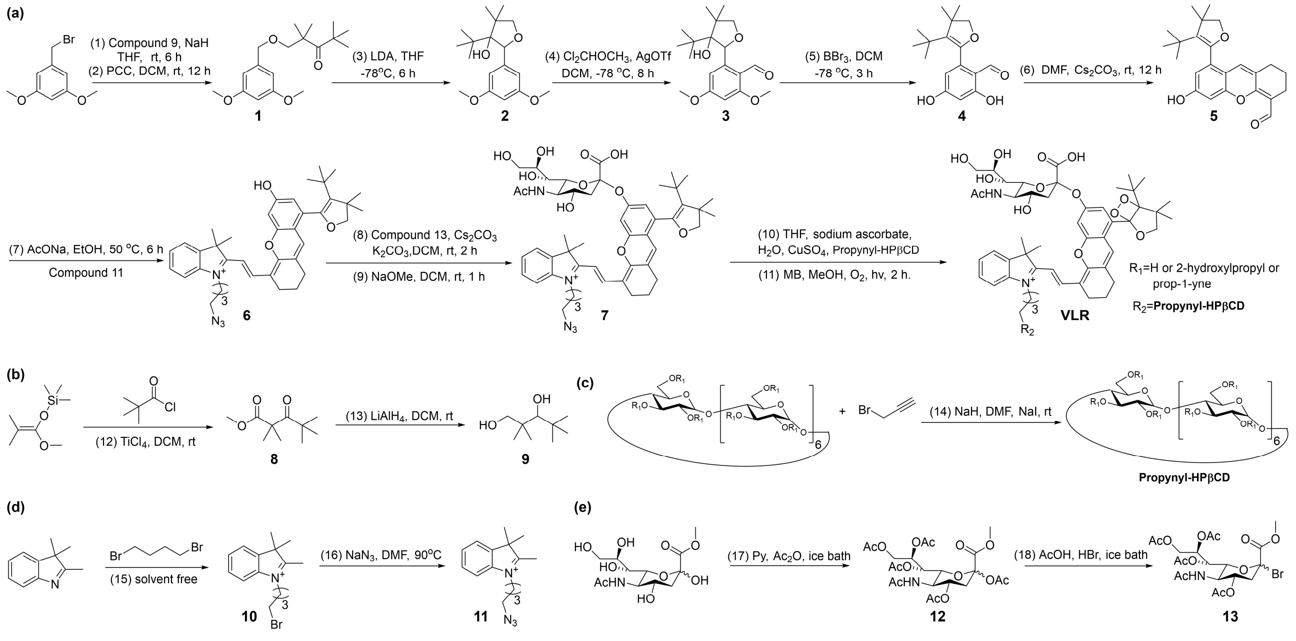

Supplementary Fig. 1 a Synthetic routes of VLR. Reagent and conditions: (1) Compound **9**, sodium hydride, THF, rt, 6 h. (2) Pyridinium chlorochromate, DCM, rt, 12 h. (3) Lithium diisopropylamide, THF, -78 °C, 6 h. (4) Silver trifluoromethanesulfonate, dichloromethyl methyl ether, DCM, -78 °C, 8 h. (5) Boron tribromide, DCM, -78 °C, 3 h. (6) DMF, Cs_2CO_3, rt, 12 h. (7) Compound **11**, AcONa, EtOH, 50 °C, 6 h. (8) Compound **13**, Cs_2CO_3, K_2CO_3, DCM, rt, 2 h. (9) NaOMe, DCM, rt, 1 h. (10) Propynyl-HPβCD, sodium ascorbate, $CuSO_4$, THF, H_2O, rt, 2 h. (11) Methylene blue, MeOH, O_2, hv, 2 h. **b** (12) Titanium tetrachloride, DCM, rt. (13) Lithium Aluminum Hydride, THF, 0 °C. **c** (14) Sodium hydride, DMF, Sodium iodide, rt. **d** (15) Solvent free, 50 °C. (16) Sodium azide, DMF, 90 °C. **e** (17) Pyridine, acetic anhydride, ice bath. (18) Acetic acid, hydrobromic acid, ice bath.

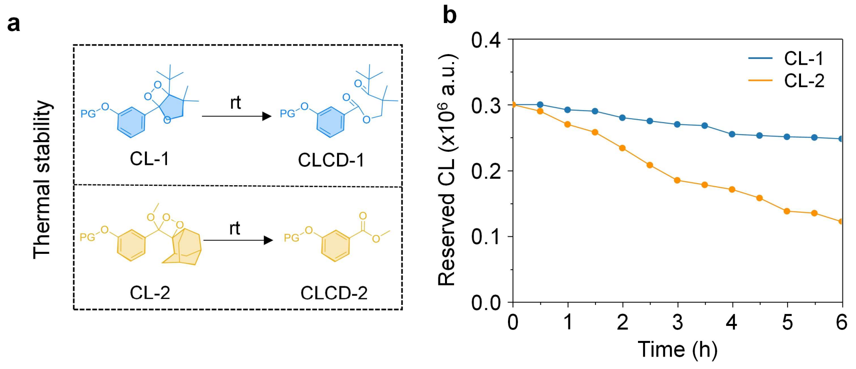

Supplementary Fig. 2. a Chemiexcitation pathway of adamantyl-dioxetanes and bicyclic dioxetanes. **b** Evaluation of the thermal stability of CL-1and CL-2. Comparison of the reserved CL intensities of CL-1 and CL-2 when stored at 25 °C for different duration time. (n = 3, mean ± s.d.).

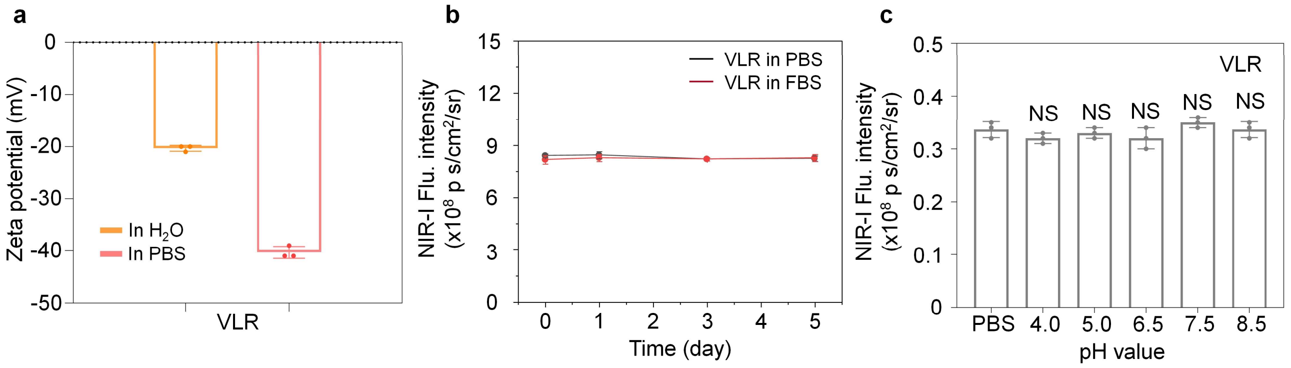

Supplementary Fig. 3. a Zeta potential of VLR in H_2O and PBS (10 mM, pH 7.4). **b** NIR fluorescence intensity of VLR as a function of time incubated with PBS and FBS, respectively. **c** Change in NIR fluorescence signals of VLR in different pH buffer solutions. NS: no statistically significant differences.

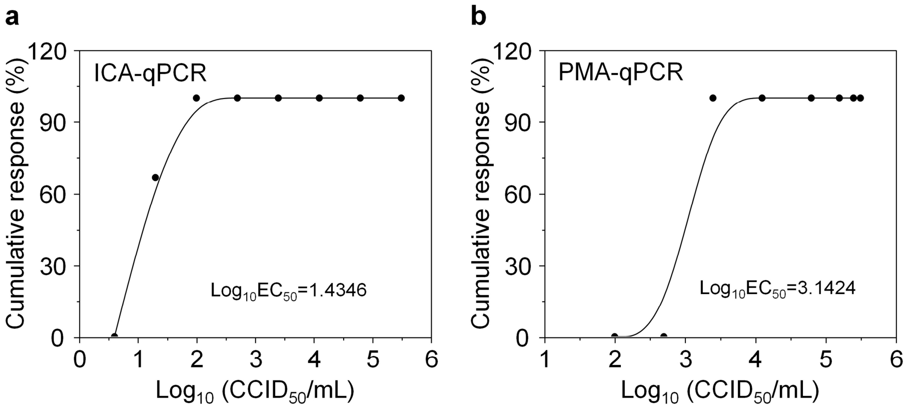

Supplementary Fig. 4. a, b Probit analysis of ICA-qPCR and PMA-qPCR methods for viable H1N1 virus detection.

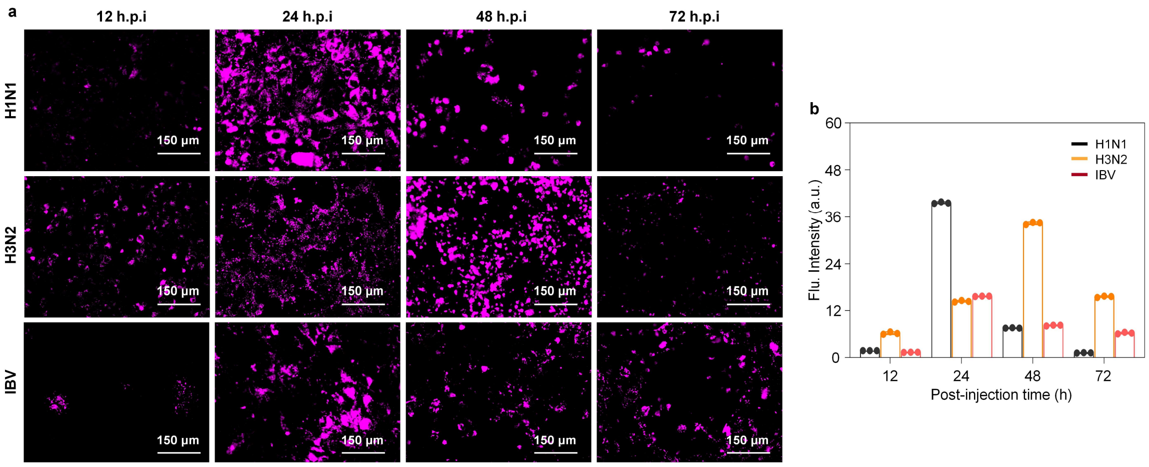

Supplementary Fig. 5. a Fluorescence images and **b** fluorescence intensity of H1N1-infected, H3N2-infected and IBV-infected MDCK cells incubated with VLR (50 μM) at 12 h, 24 h, 48 h and 72 h post-injection. (Scale bar: 150 μm).

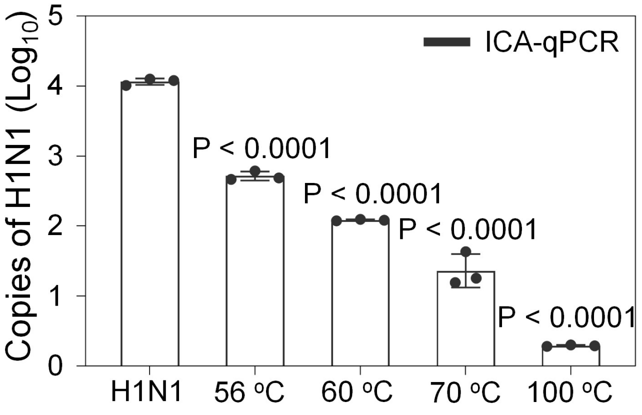

Supplementary Fig. 6. The copy numbers of H1N1 virus after thermal inactivation treatment. Viral copy numbers were measured using ICA-qPCR assay.

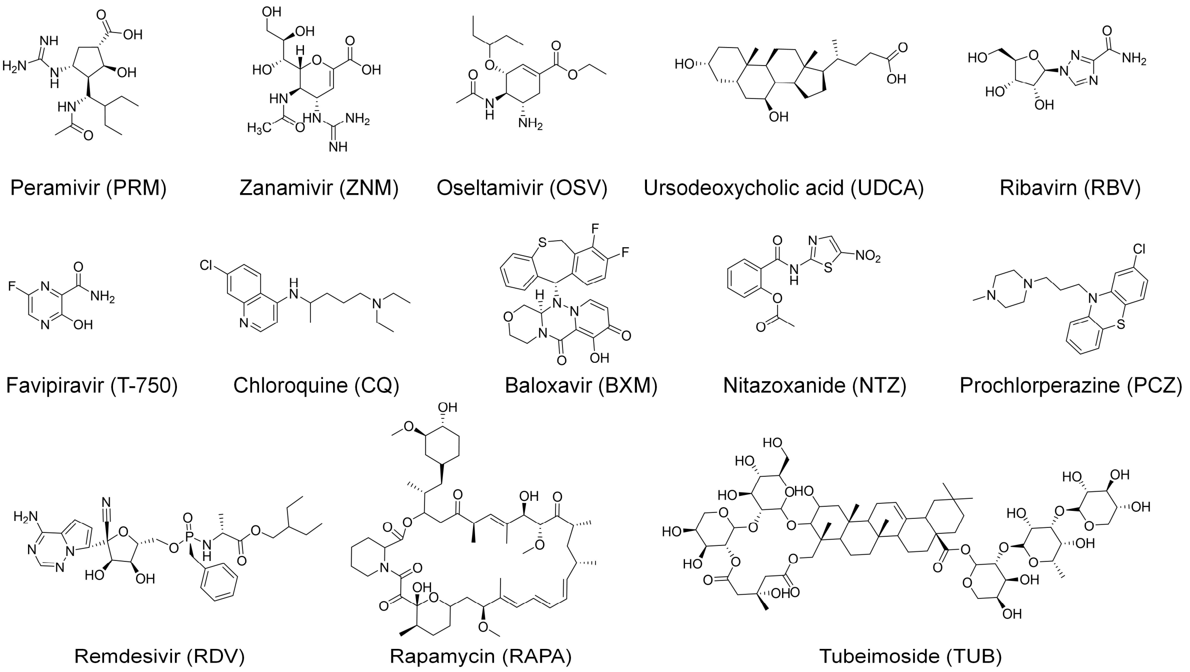

Supplementary Fig. 7. The chemical structures of various antiviral medications used in this study including peramivir (PRM), zanamivir (ZNM), oseltamivir (OSV), ursodeoxycholic acid (UDCA), ribavirn (RBV), favipiravir (T-750), chloroquine (CQ), baloxavir (BXM), nitazoxanide (NTZ), prochlorperazine (PCZ), remdesivir (RDV), rapamycin (RAPA) and tubeimoside (TUB).

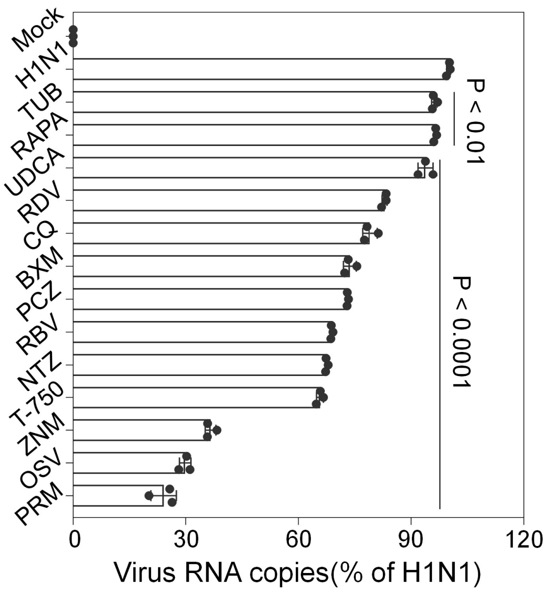

Supplementary Fig. 8. Virus RNA copies (% of H1N1) after various antiviral medications treatment.

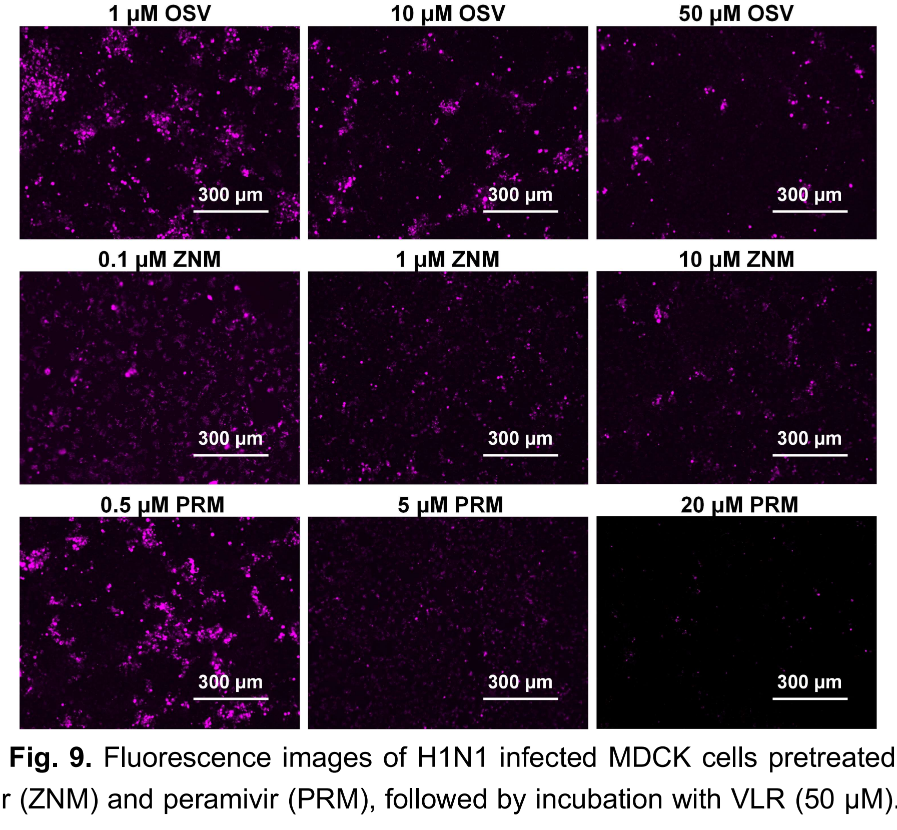

Supplementary Fig. 9. Fluorescence images of H1N1 infected MDCK cells pretreated with oseltamivir (OSV), zanamivir (ZNM) and peramivir (PRM), followed by incubation with VLR (50 μM). (Scale bar: 300 μm).

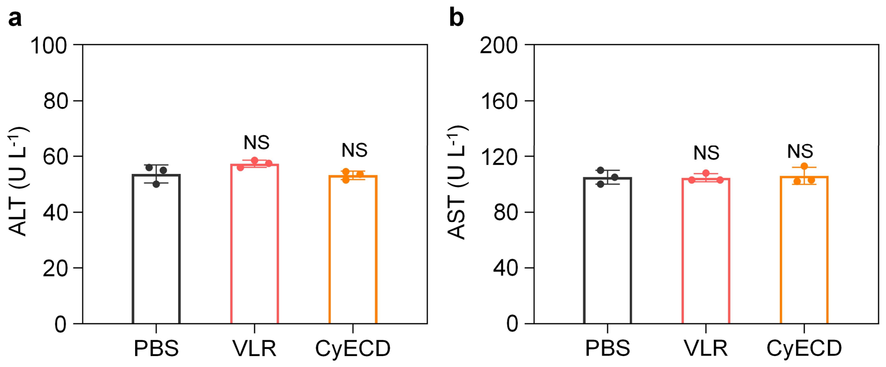

Supplementary Fig. 10. a, b Measurements of ALT and AST from living mice after i.t. injection of PBS, VLR and CyECD. Data in (a-b) are the mean ± SD. n=3 independent experiments. Two-tailed Student's t-test. PBS group versus experimental group. NS: no statistically significant differences.

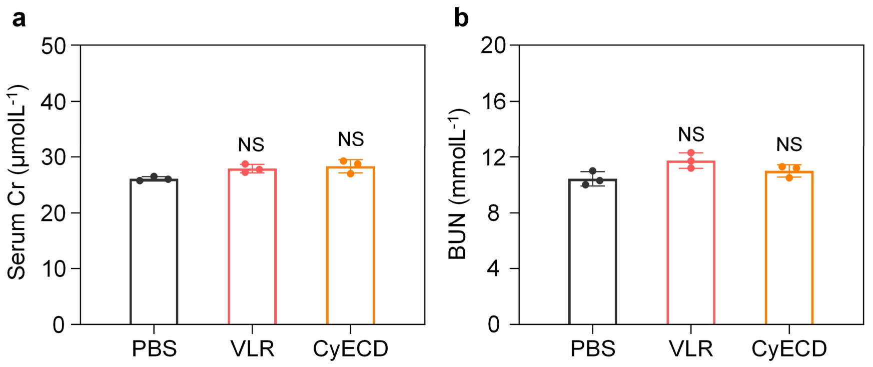

Supplementary Fig. 11. a, b Measurements of sCr and BUN from living mice after i.t. injection of PBS, VLR and CyECD. Data in (a-d) are the mean ± SD. n=3 independent experiments. Two-tailed Student's t-test. PBS group versus experimental group. NS: no statistically significant differences.

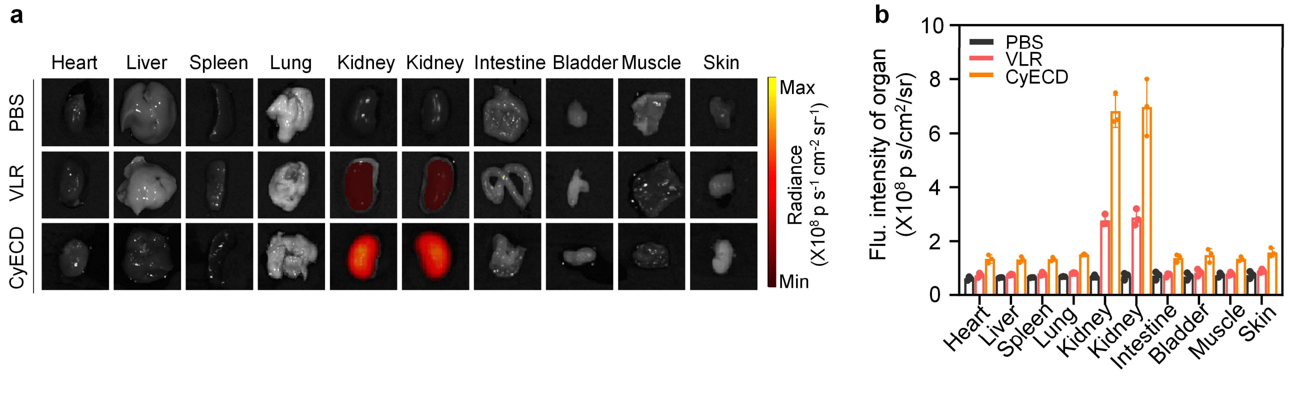

Supplementary Fig. 12. a Representative NIR fluorescence images of resected organs from mice after 1 h post-injection of PBS, VLR and CyECD (10 μmol kg^{-1} body weight). **b** Ex vivo NIR fluorescence

quantification of major organ in the panel a. Dates are the mean ± SD. n=3 independent mice.

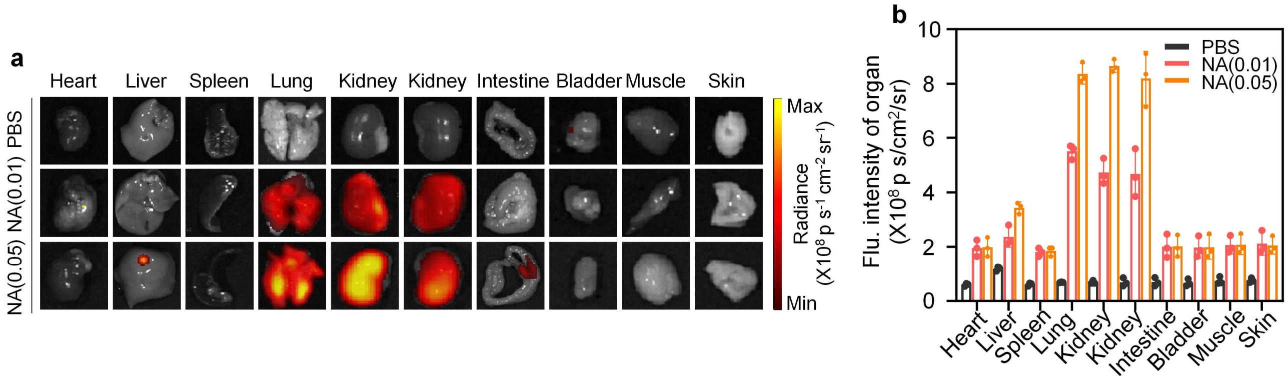

Supplementary Fig. 13. a Representative fluorescence images of resected organs from mice after treatment of PBS, NA (0.01 mg), or NA (0.05 mg), followed by injection of VLR (10 μmol kg^{-1} body weight). **b** Ex vivo fluorescence quantification of major organ of mice after treatment of PBS, NA (0.01 mg), or NA (0.05 mg). Dates are the mean ± SD. n=3 independent mice.

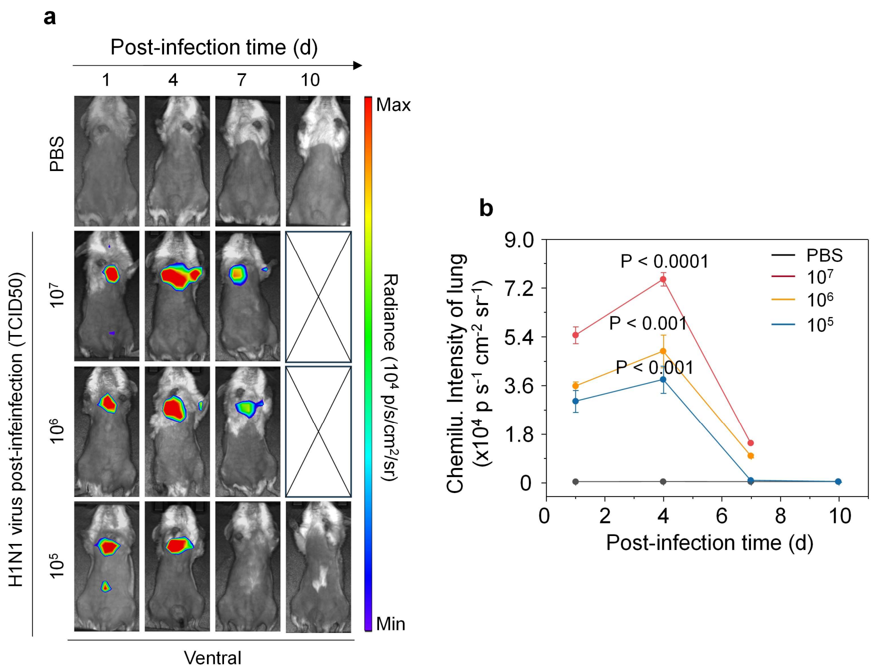

Supplementary Fig. 14. a Representative chemiluminescence images of living mice after infection of different titers virus, followed by injection of VLR. Chemiluminescence images acquired under bioluminescence mode of IVIS spectrum imaging system with the acquisition time of 60 s. **b** The dynamic chemiluminescence intensities of lungs in living mice at different post-infection time. Dates are the mean ± SD. n=3 independent mice.

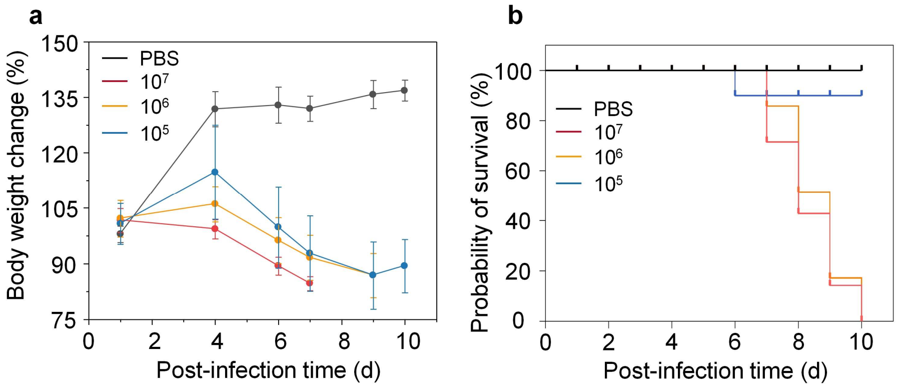

Supplementary Fig. 15. a Change in body weight of living mice throughout the experimental period at different post-infection time. **b** The probability of survival of mice for different groups at different post-infection time. Dates are the mean ± SD. n=3 independent mice.

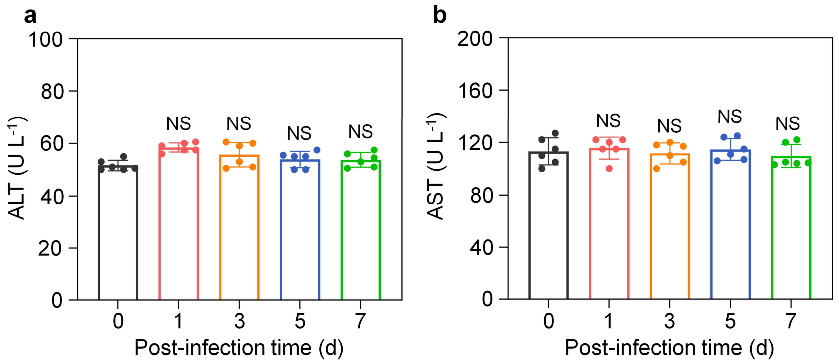

Supplementary Fig. 16. a, b Measurements of ALT and AST from living mice at different post-infection time. Data in (a-b) are the mean ± SD. n=6 independent experiments. Two-tailed Student's t-test. Control group versus experimental group. NS: no statistically significant differences.

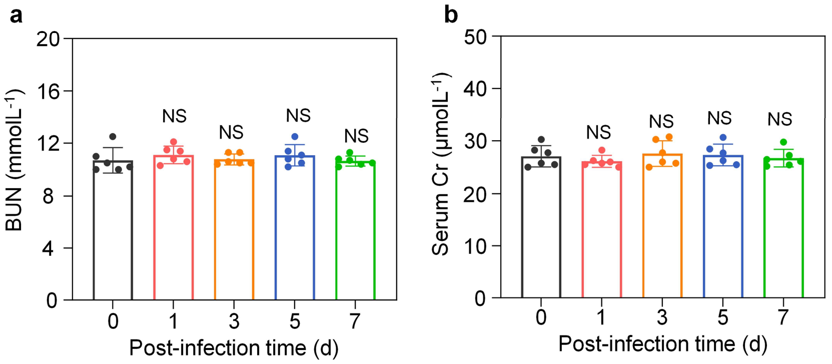

Supplementary Fig. 17. a, b Measurements of BUN and sCr from living mice at different post-infection time. Data in (a-b) are the mean ± SD. n=6 independent experiments. Two-tailed Student's t-test. Control group versus experimental group. NS: no statistically significant differences.

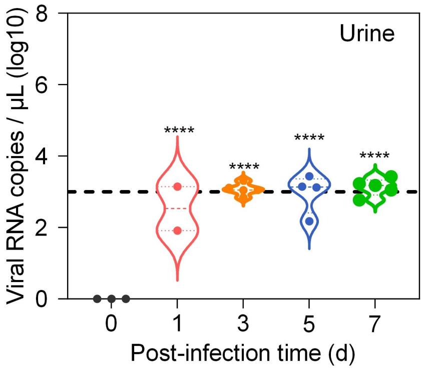

Supplementary Fig. 18. The copy number of virus in the urine at different post-infection time. Dates are the mean ± SD. n=5 independent mice.

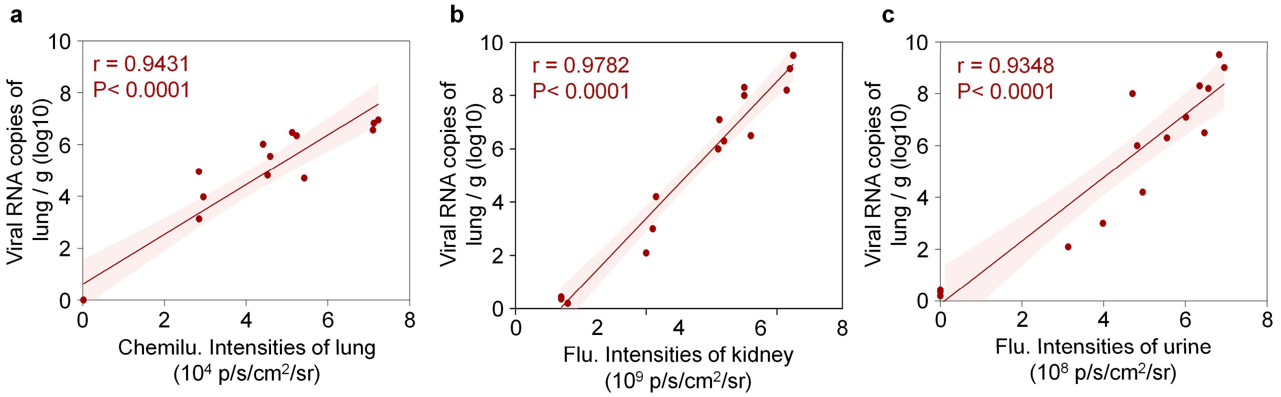

Supplementary Fig. 19. a Correlation between the copy number of virus in the lungs and the in vivo NIRC signals via a simple linear regression model. The 95% confidence intervals were obtained by two-tailed Student's t-test analysis. **b** Correlation between the copy number of virus in the lung and the kidney NIRF signals via a simple linear regression model. The 95% confidence intervals were obtained by two-tailed Student's t-test analysis. **c** Correlation between the copy number of virus in the lung and the urinary NIRF signals via a simple linear regression model. The 95% confidence intervals were obtained by two-tailed Student's t-test analysis.

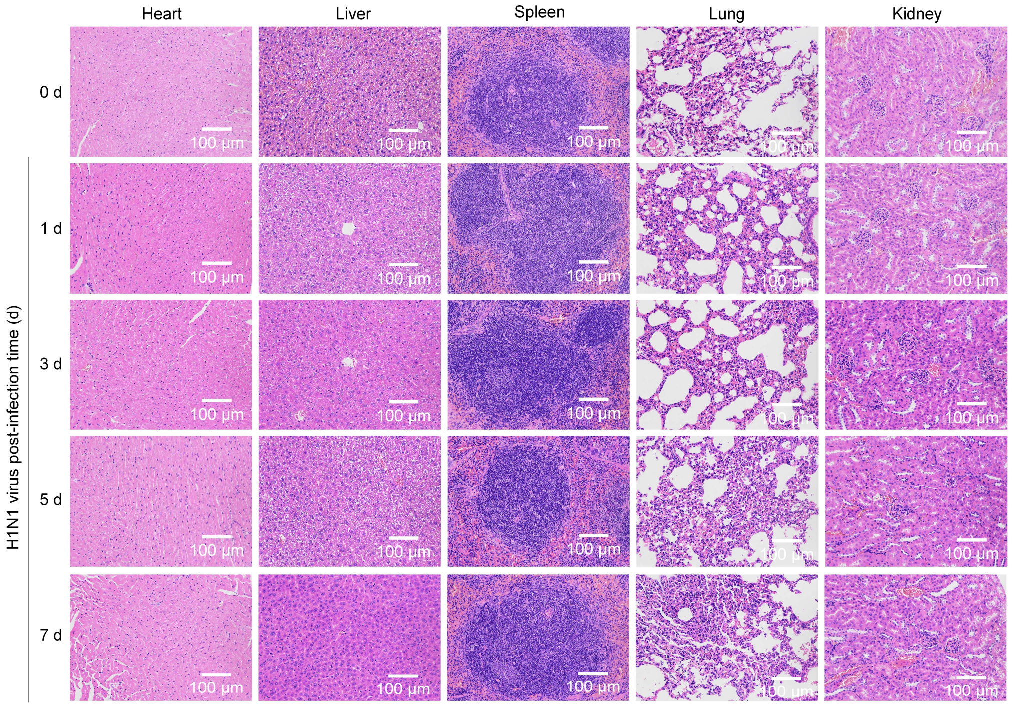

Supplementary Fig. 20. H&E staining of major organs including heart, liver, spleen, lung, and kidney from mice at different post-infection time. (Scale bar: 100 μm).

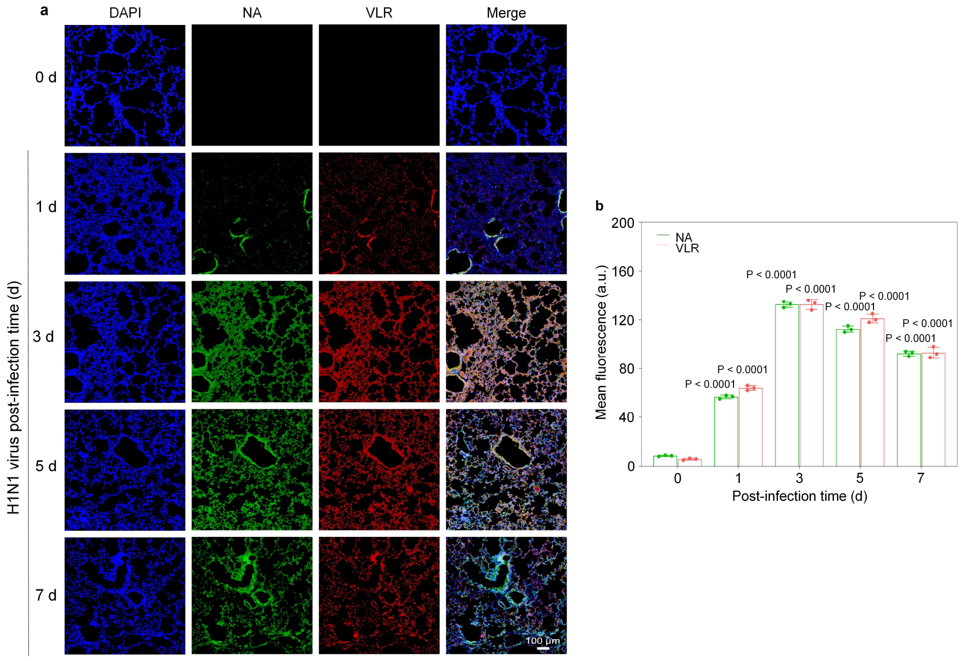

Supplementary Fig. 21. a Confocal fluorescence microscopy images of lung slices from mice at different post-infection time. The blue, green and red signals come from DAPI, NA antibody staining and activated VLR, respectively (scale bar=100 μm). **b** Mean fluorescence intensity of NA antibody staining and activated VLR in the panel **a**. Data are the mean ± SD. n=3 independent experiments. Two-tailed Student's t-test. PBS group versus experimental groups (**p < 0.01, ****p < 0.0001).

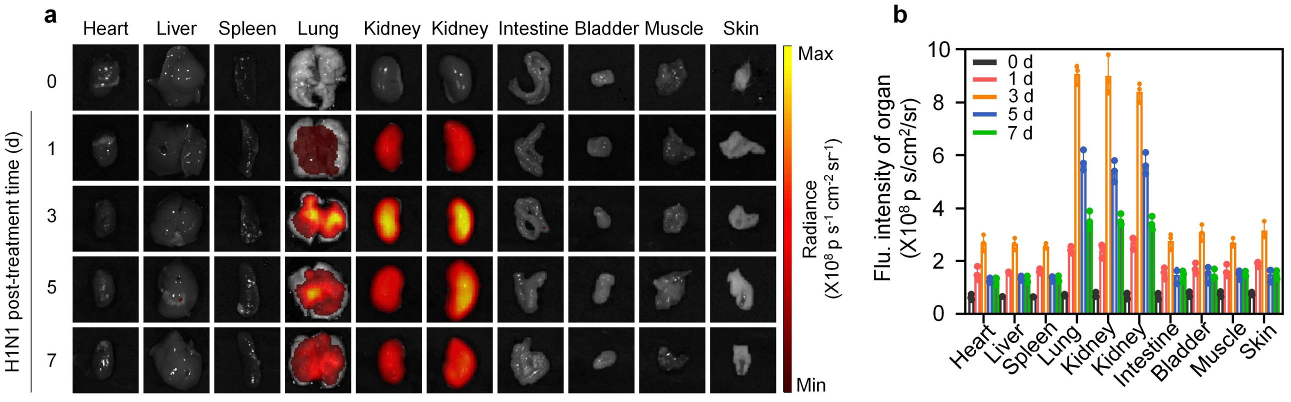

Supplementary Fig. 22. a Representative fluorescence images of resected organs from mice at different post-infection time. **b** Ex vivo fluorescence quantification of major organ in the panel a. Dates are the mean ± SD. n=3 independent mice.

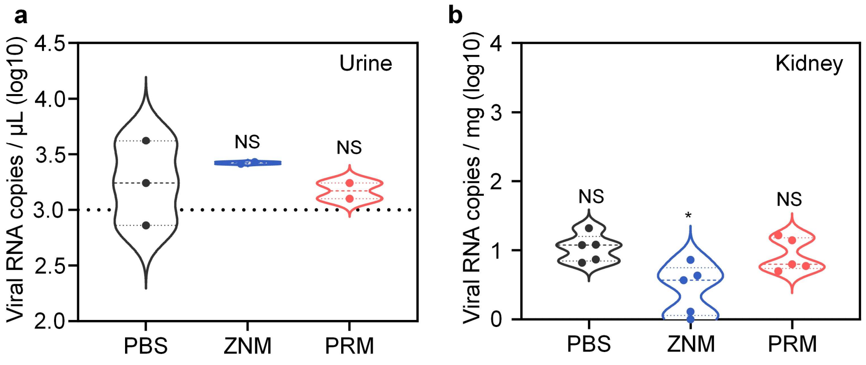

Supplementary Fig. 23. a, b The copy numbers of virus in the urine and kidney with different antiviral drugs treatment. Dates are the mean ± SD. n=5 independent mice.

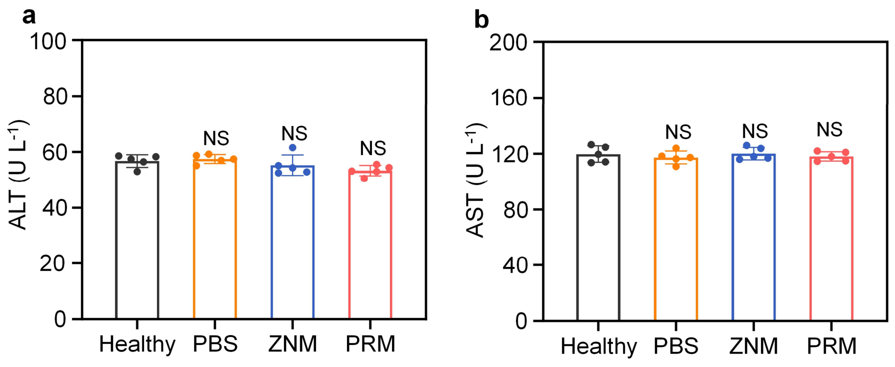

Supplementary Fig. 24. a, b Measurements of ALT and AST from living mice with different antiviral drugs treatment. Data in (a-b) are the mean ± SD. n=5 independent experiments. Two-tailed Student's t-test. Control group versus drug treatment group. NS: no statistically significant differences.

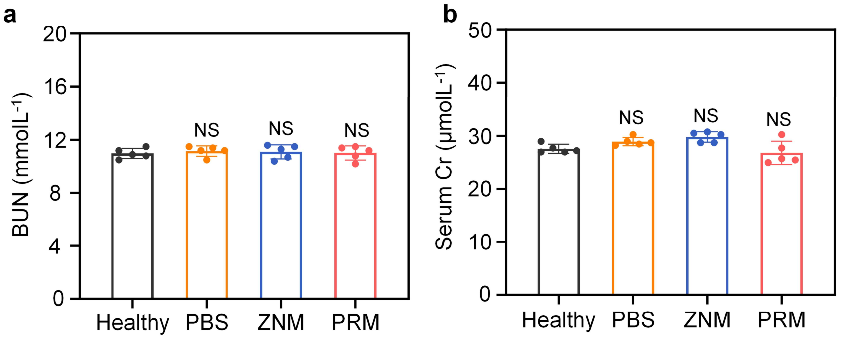

Supplementary Fig. 25. a, b Measurements of BUN and sCr from living mice with different antiviral drugs treatment. Data in (a-b) are the mean ± SD. n=5 independent experiments. Two-tailed Student's t-test. Control group versus drug treatmentgroup. NS: no statistically significant differences.

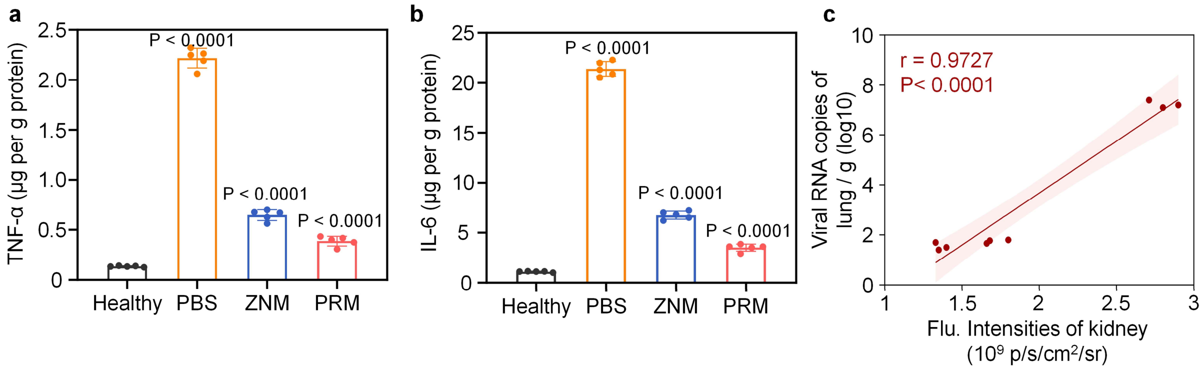

Supplementary Fig. 26. a, b Measurements of IL-6 and TNF-α from living mice with different antiviral drugs treatment. Data in (a-b) are the mean ± SD. n=5 independent experiments. Two-tailed Student's t-test. Control group versus drug treatment groups. NS: no statistically significant differences. **c** Correlation between the copy number of virus in the lungs and NIRF signals of kidney via a simple linear regression model. The 95% confidence intervals were obtained by two-tailed Student's t-test analysis.

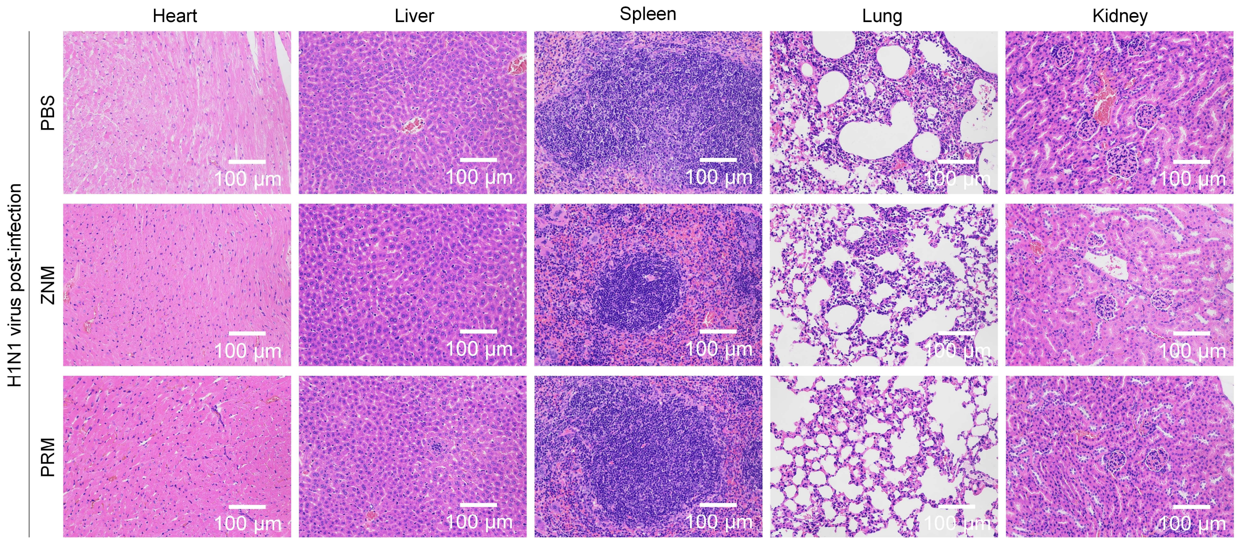

Supplementary Fig. 27. HE staining of major organs including heart, liver, spleen, lung, and kidney from mice with different antiviral drugs treatment. (Scale bar: 100 μm).

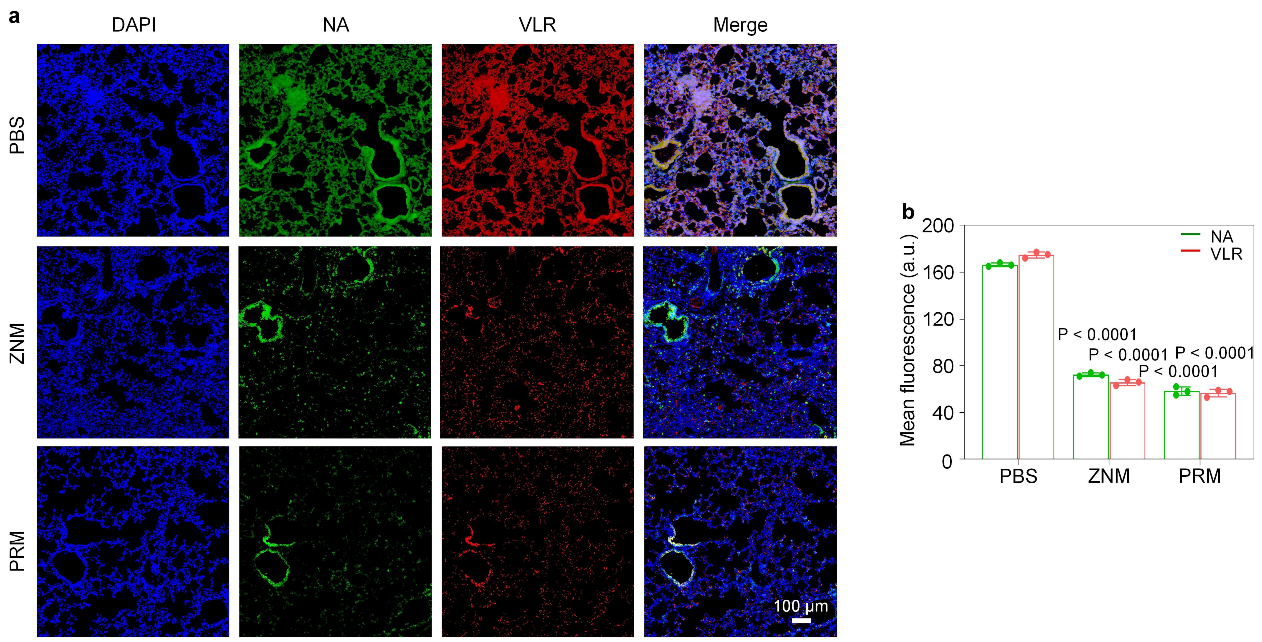

Supplementary Fig. 28. a Confocal fluorescence microscopy images of lung slices from mice with different antiviral drugs treatment. The blue, green and red signals come from DAPI, NA antibody staining and activated VLR, respectively (scale bar=100 μm). **b** Mean fluorescence intensity of NA antibody staining and activated VLR in the panel of **a**. Datas are the mean ± SD. n=3 independent experiments. Two-tailed Student's t-test. PBS group versus experimental groups (**p < 0.01, ****p < 0.0001).

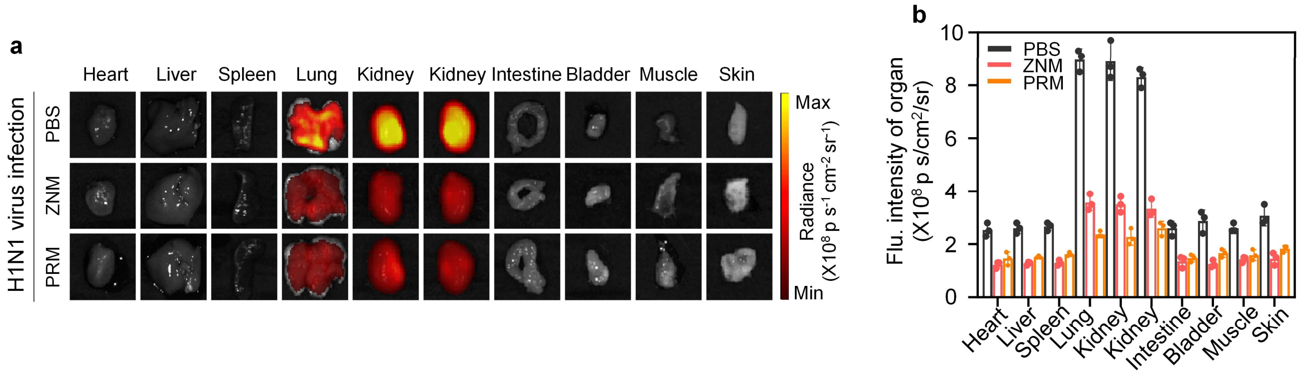

Supplementary Fig. 29. a Representative fluorescence images of resected organs from mice with different antiviral drugs treatment. **b** Ex vivo fluorescence quantification of major organ in the panel a. Dates are the mean ± SD. n=3 independent mice.

NMR and MS spectroscopy

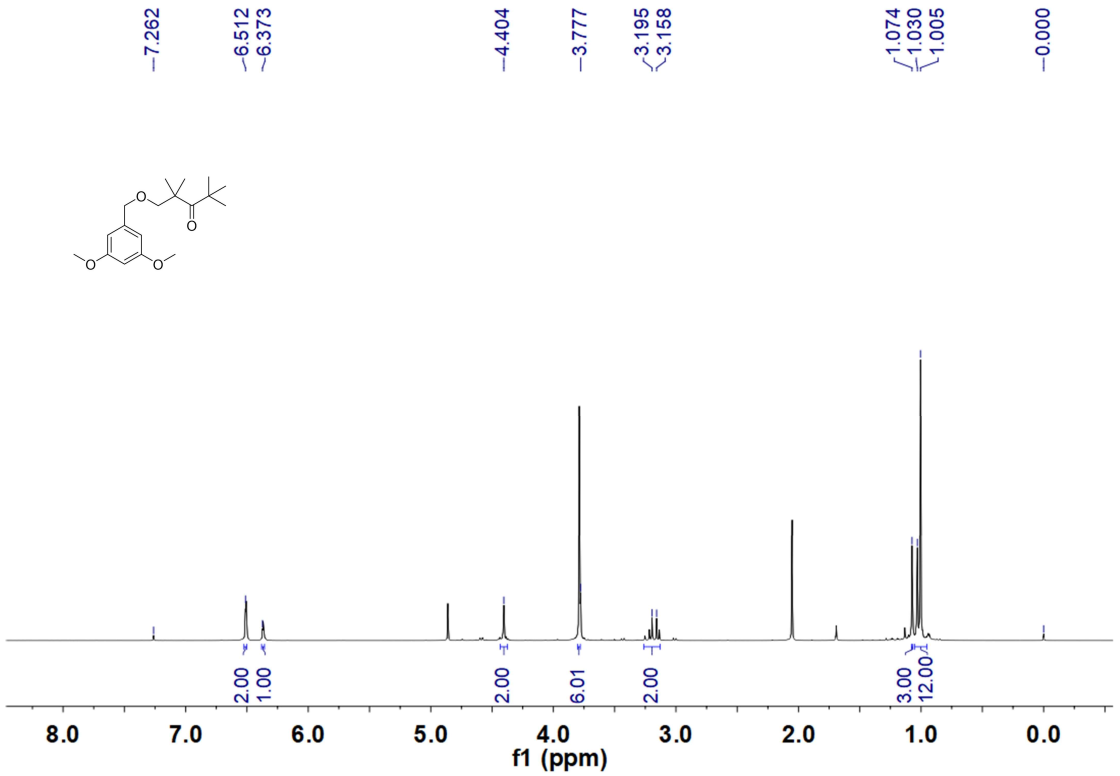

Supplementary Fig. 30. ^{1}H-NMR spectra of compound **1.**

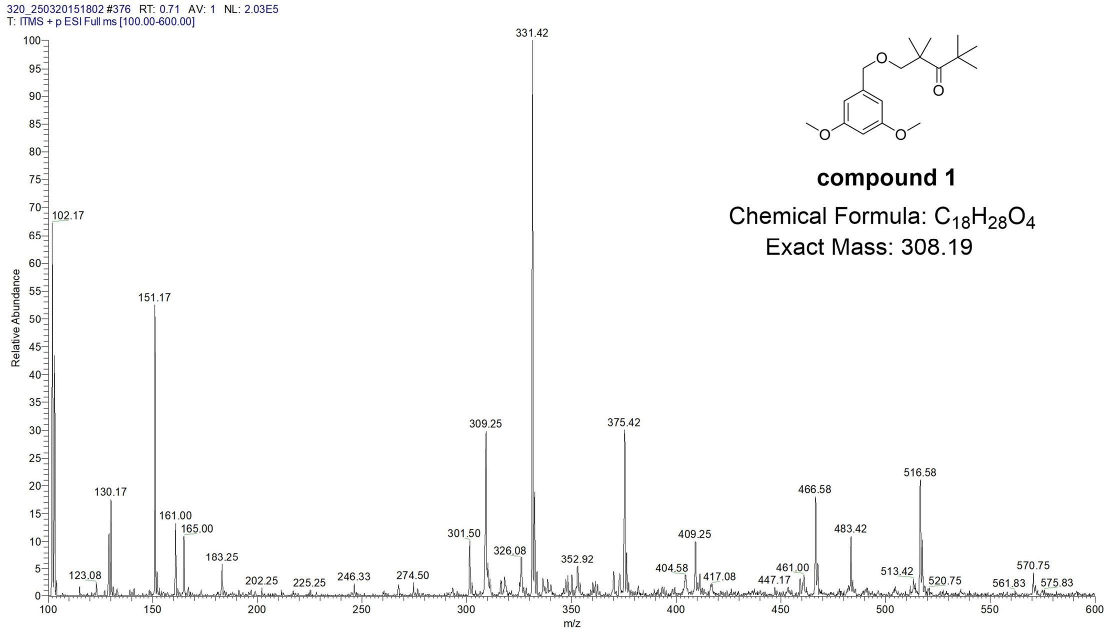

Supplementary Fig. 31. ESI-MS spectra of compound **1.**

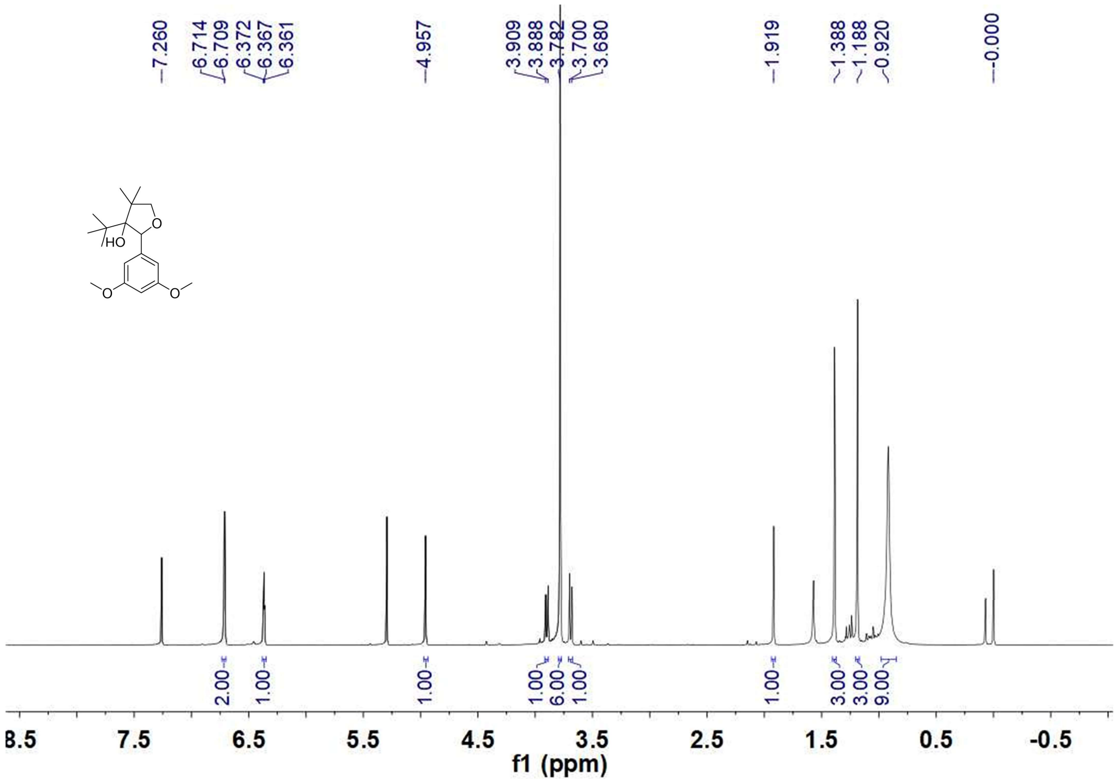

Supplementary Fig. 32. ^{1}H-NMR spectra of compound **2.**

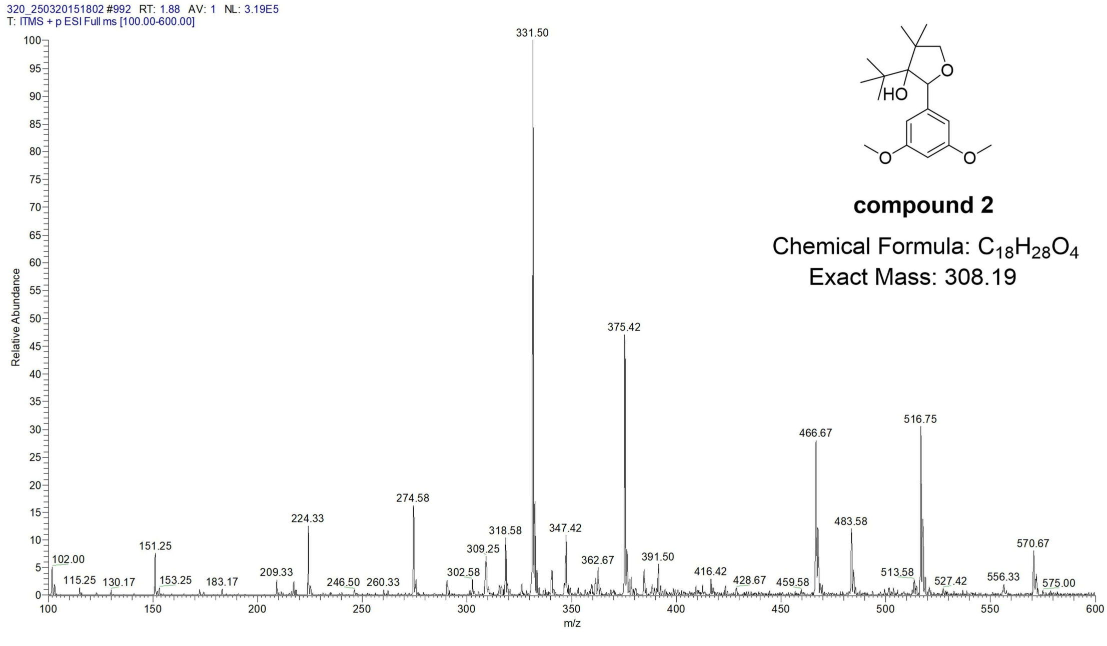

Supplementary Fig. 33. ESI-MS spectra of compound **2.**

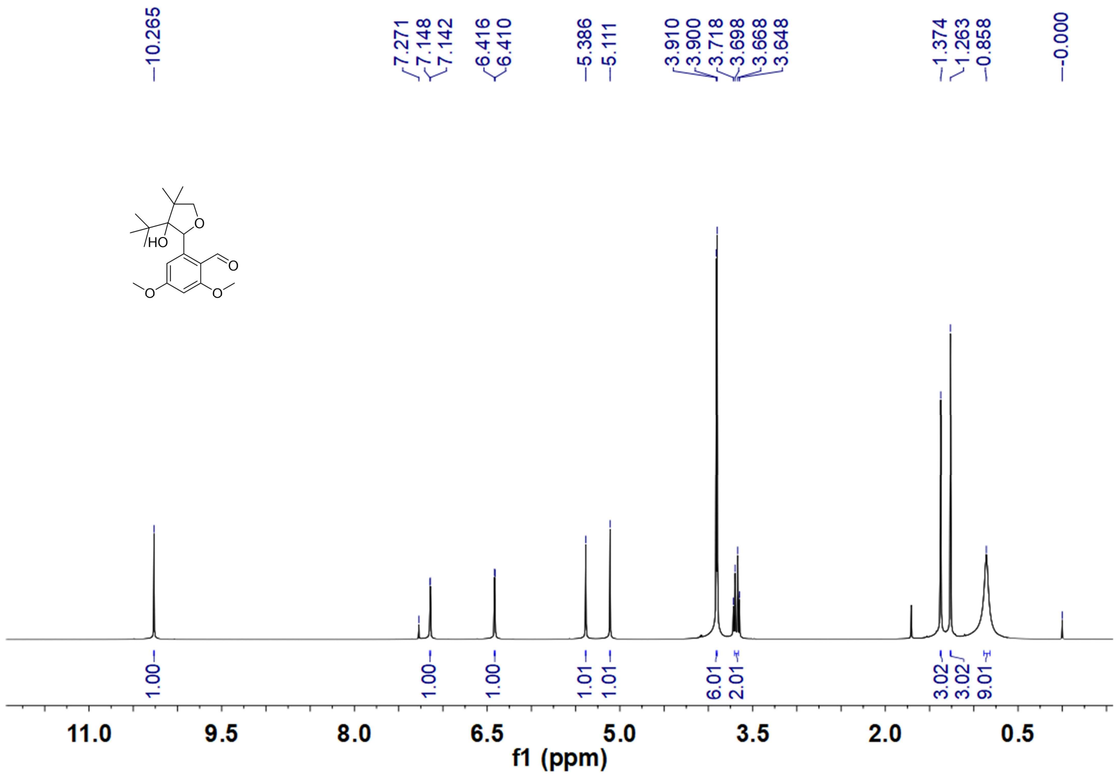

Supplementary Fig. 34. ^{1}H-NMR spectra of compound **3.**

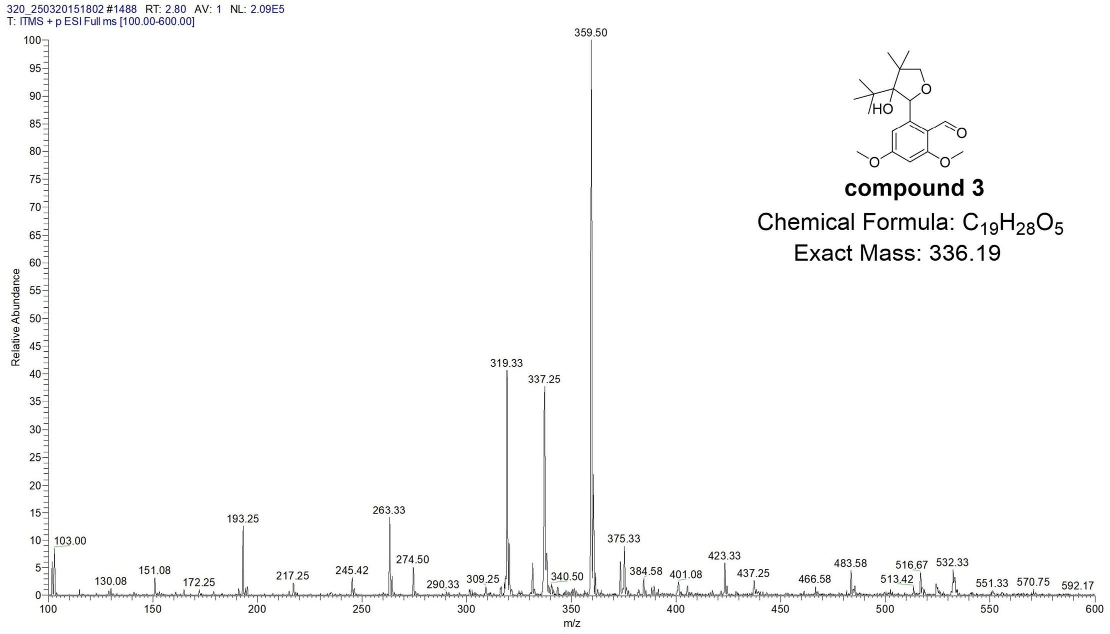

Supplementary Fig. 35. ESI-MS spectra of compound **3.**

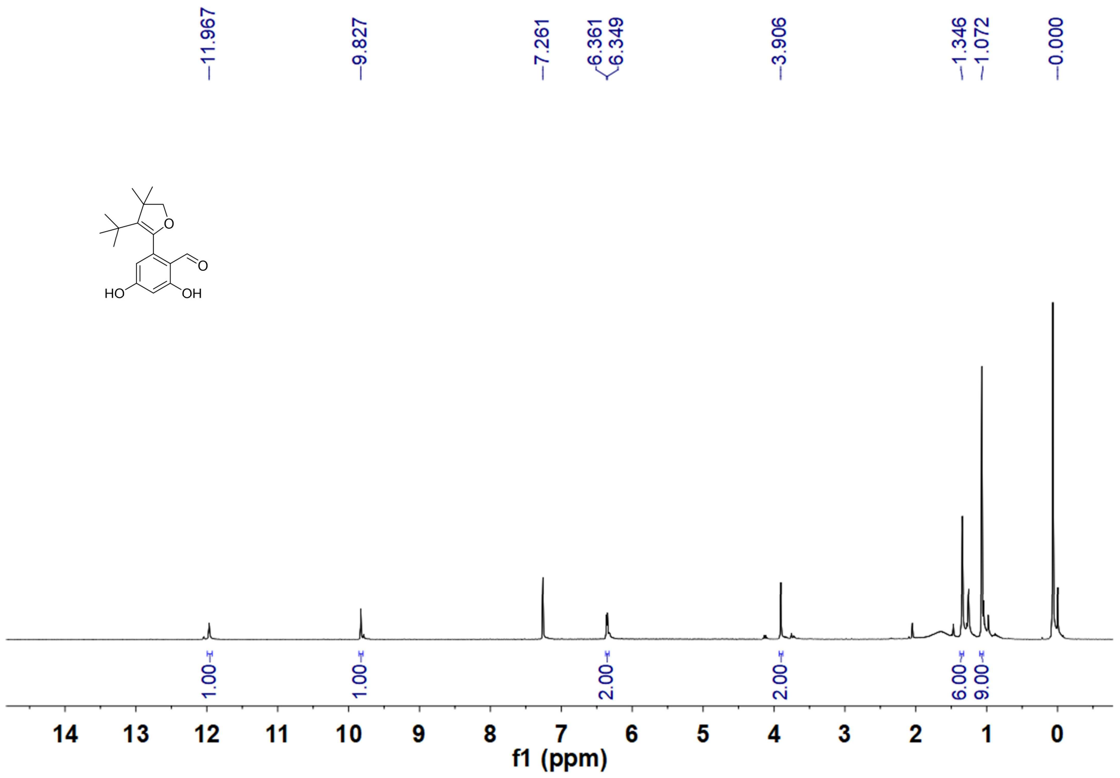

Supplementary Fig. 36. ^{1}H-NMR spectra of compound **4.**

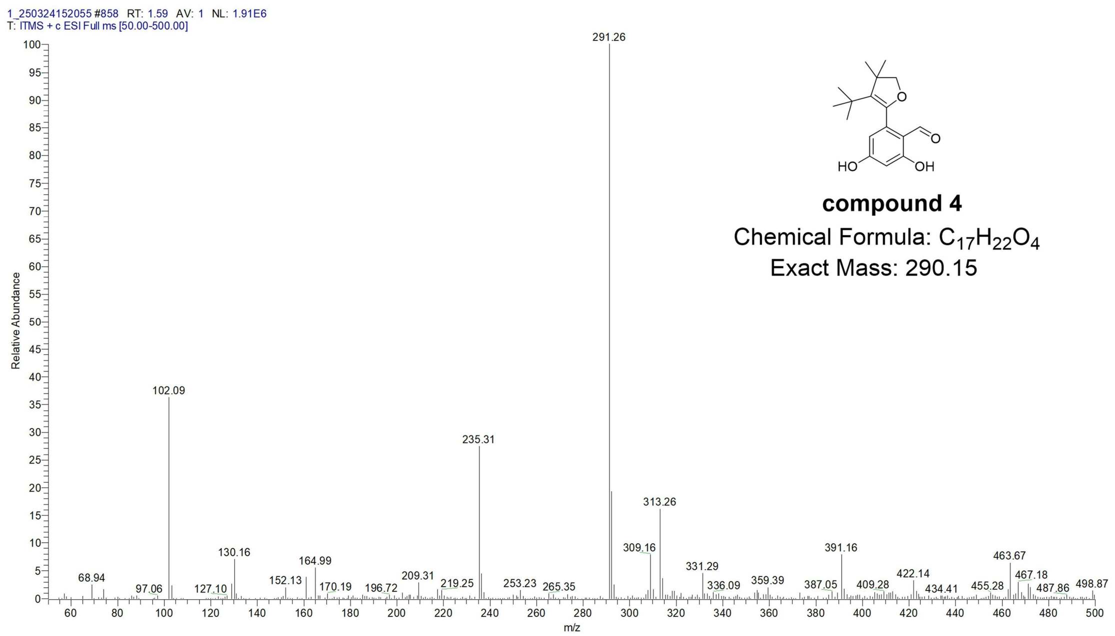

Supplementary Fig. 37. ESI-MS spectra of compound **4.**

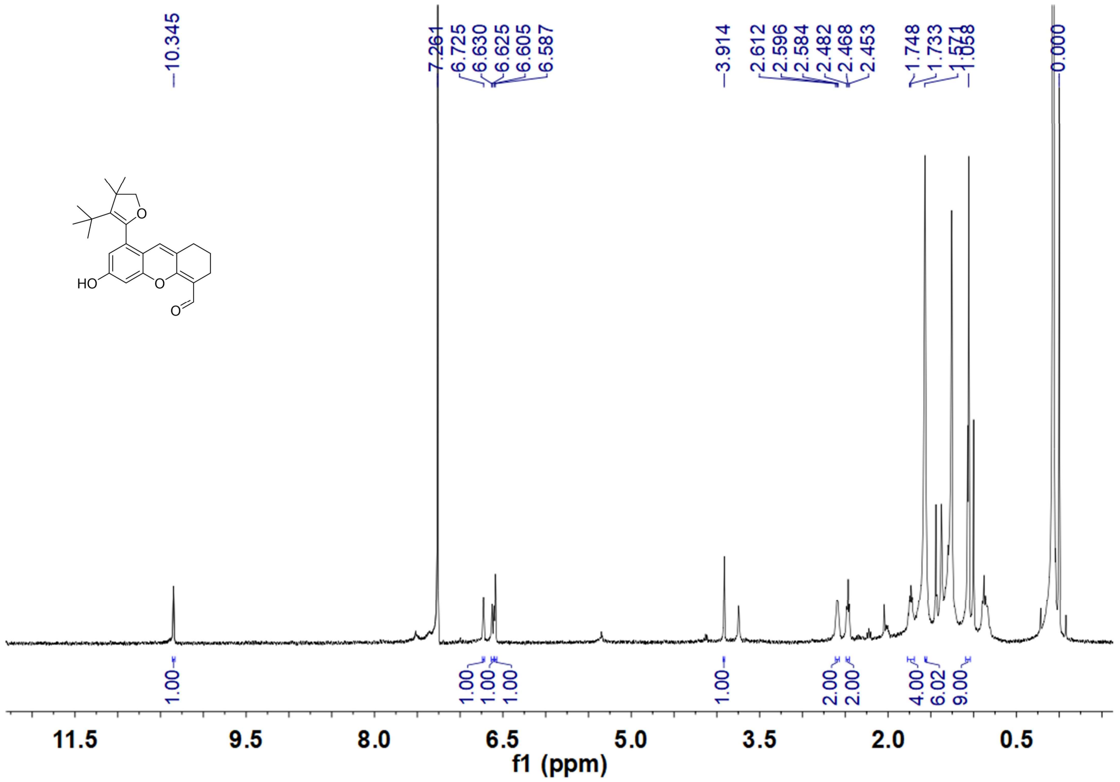

Supplementary Fig. 38. ^{1}H-NMR spectra of compound **5.**

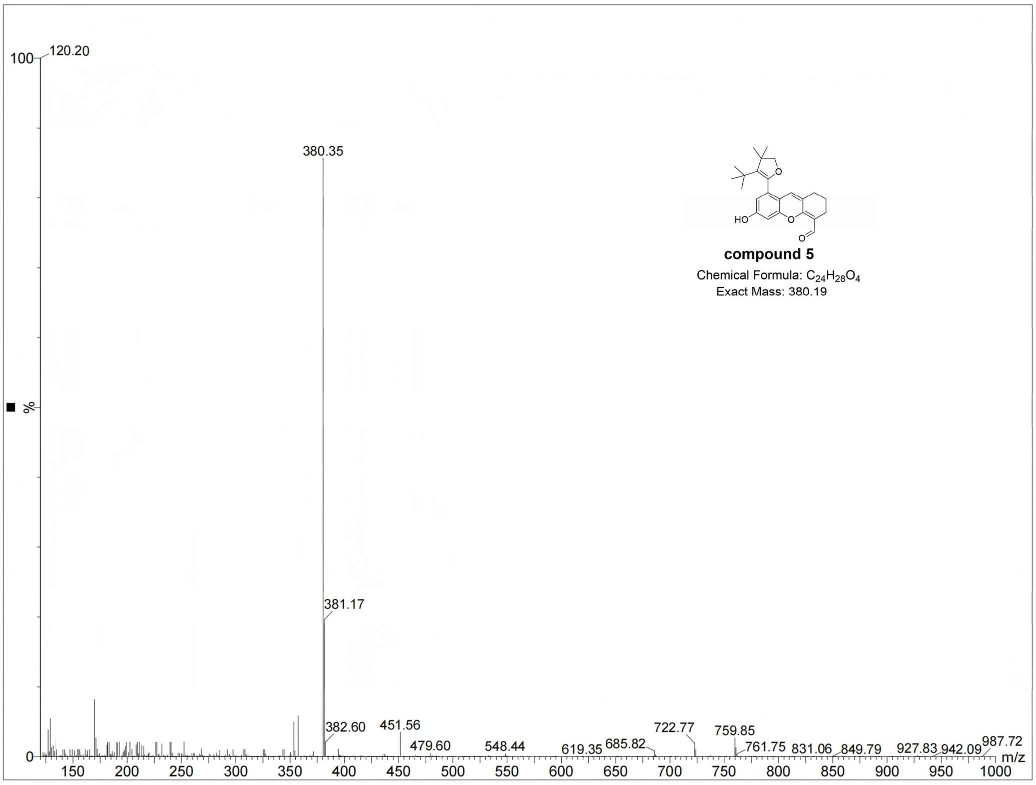

Supplementary Fig. 39. ESI-MS spectra of compound **5.**

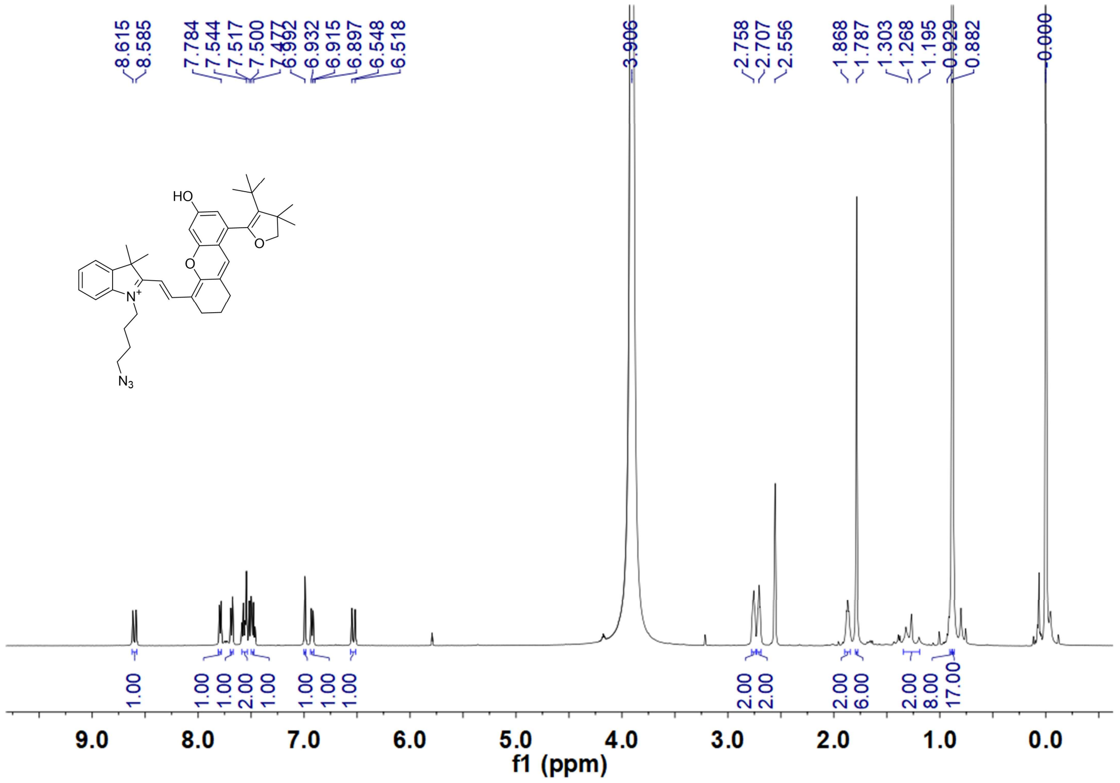

Supplementary Fig. 40. ^{1}H-NMR spectra of compound **6.**

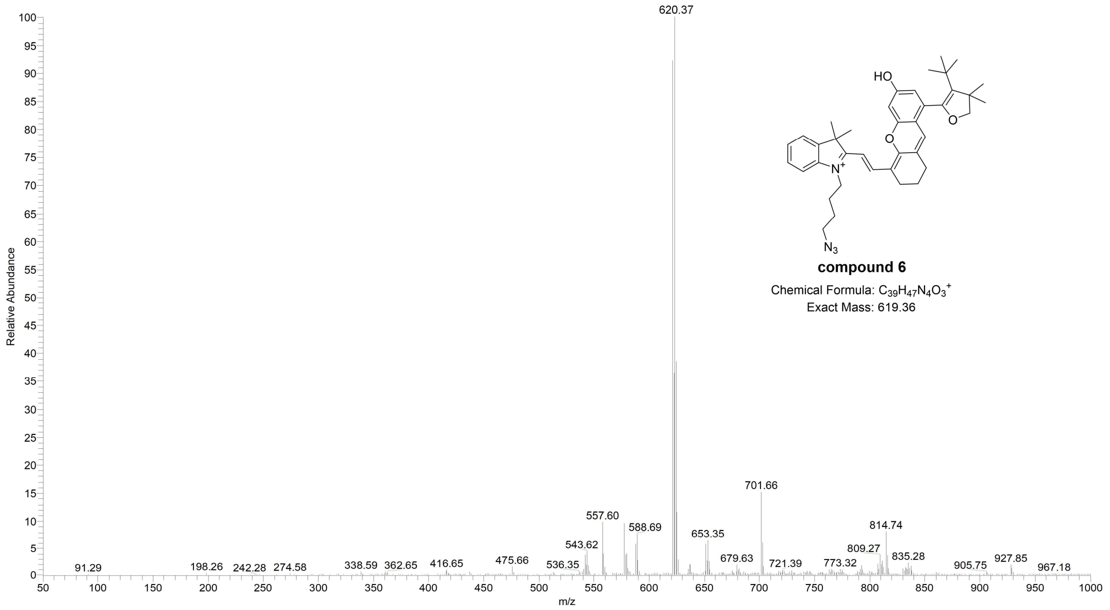

Supplementary Fig. 41. ESI-MS spectra of compound **6.**

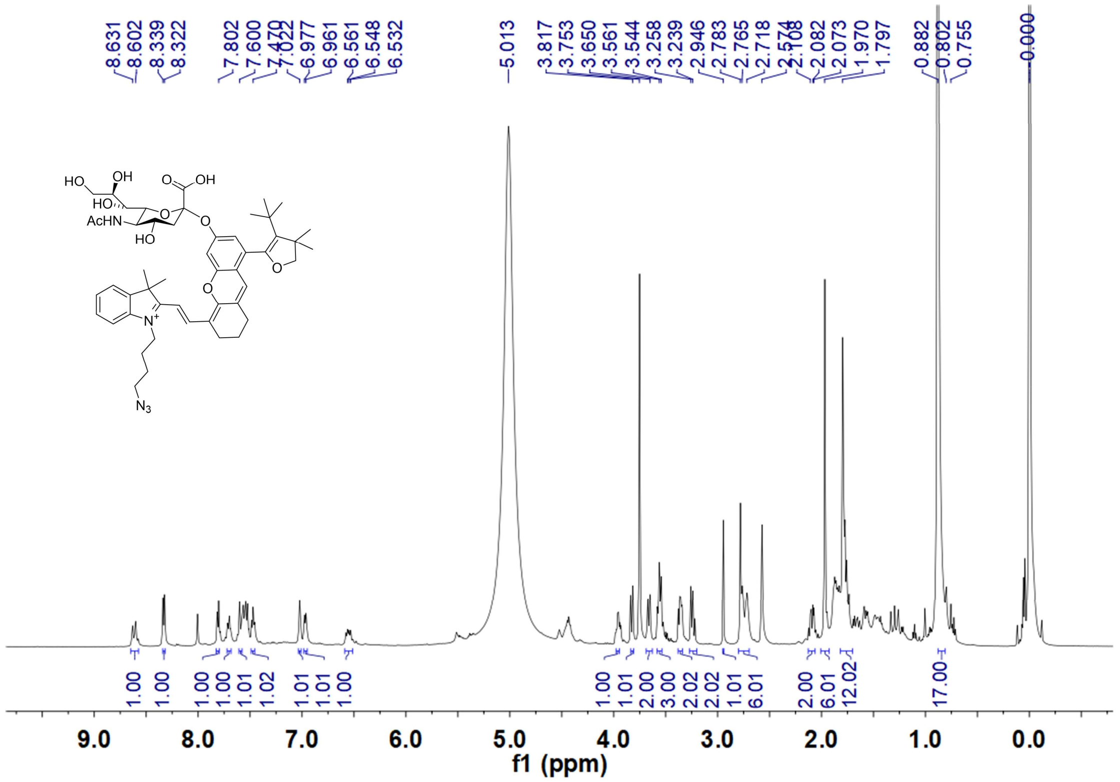

Supplementary Fig. 42. ^{1}H-NMR spectra of compound **7.**

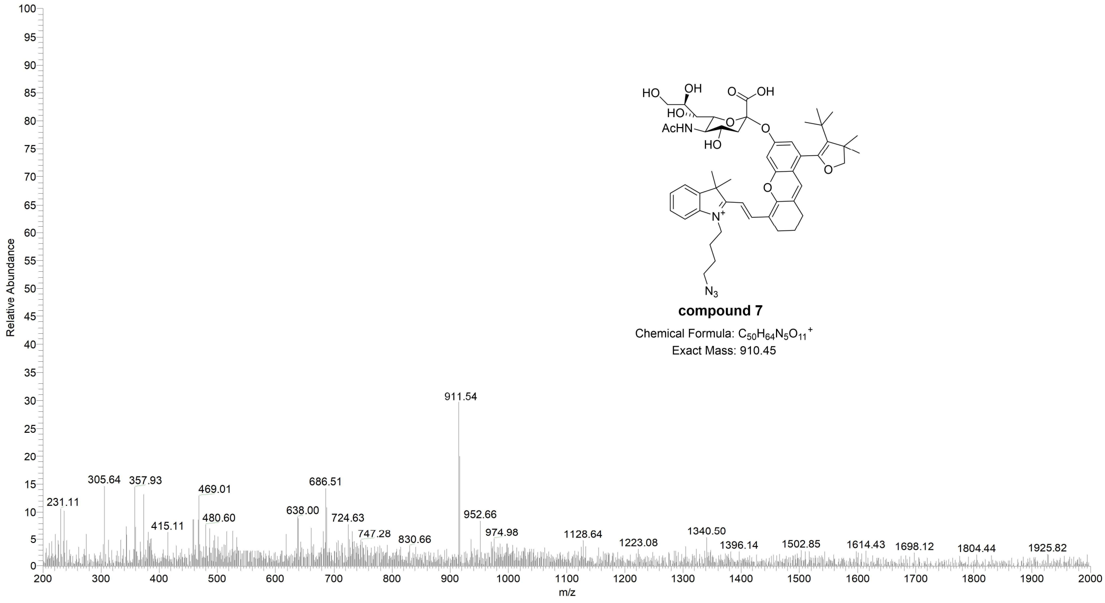

Supplementary Fig. 43. ESI-MS spectra of compound **7.**

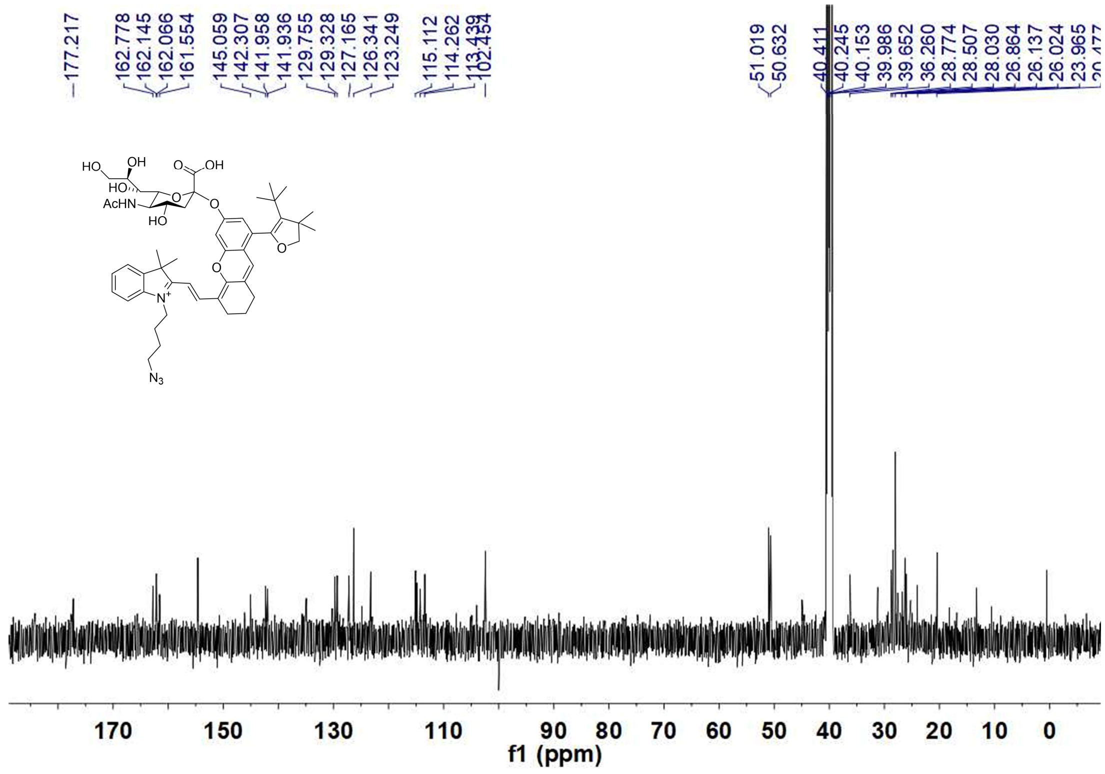

Supplementary Fig. 44. ^{13}C-NMR spectra of compound **7.**

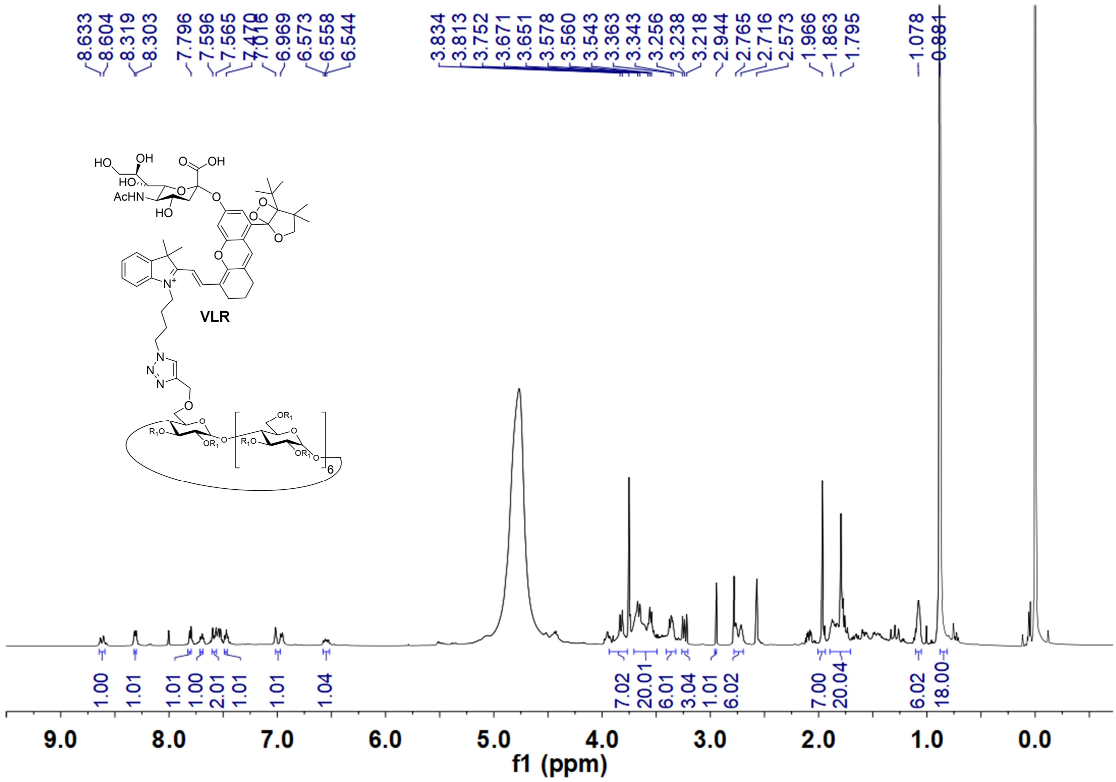

Supplementary Fig. 45. ^{1}H-NMR spectra of compound **VLR.**

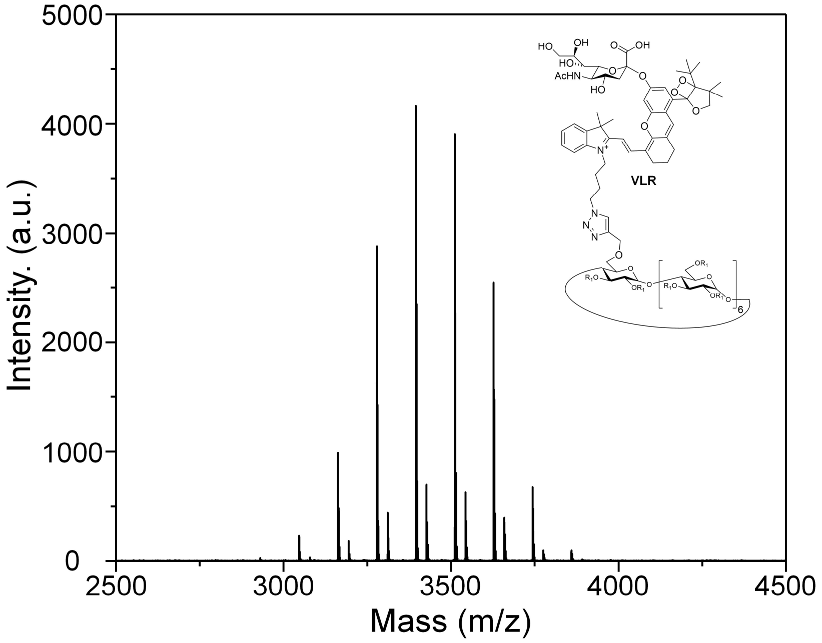

Supplementary Fig. 46. MALDI-TOF MS spectra of compound **VLR.**

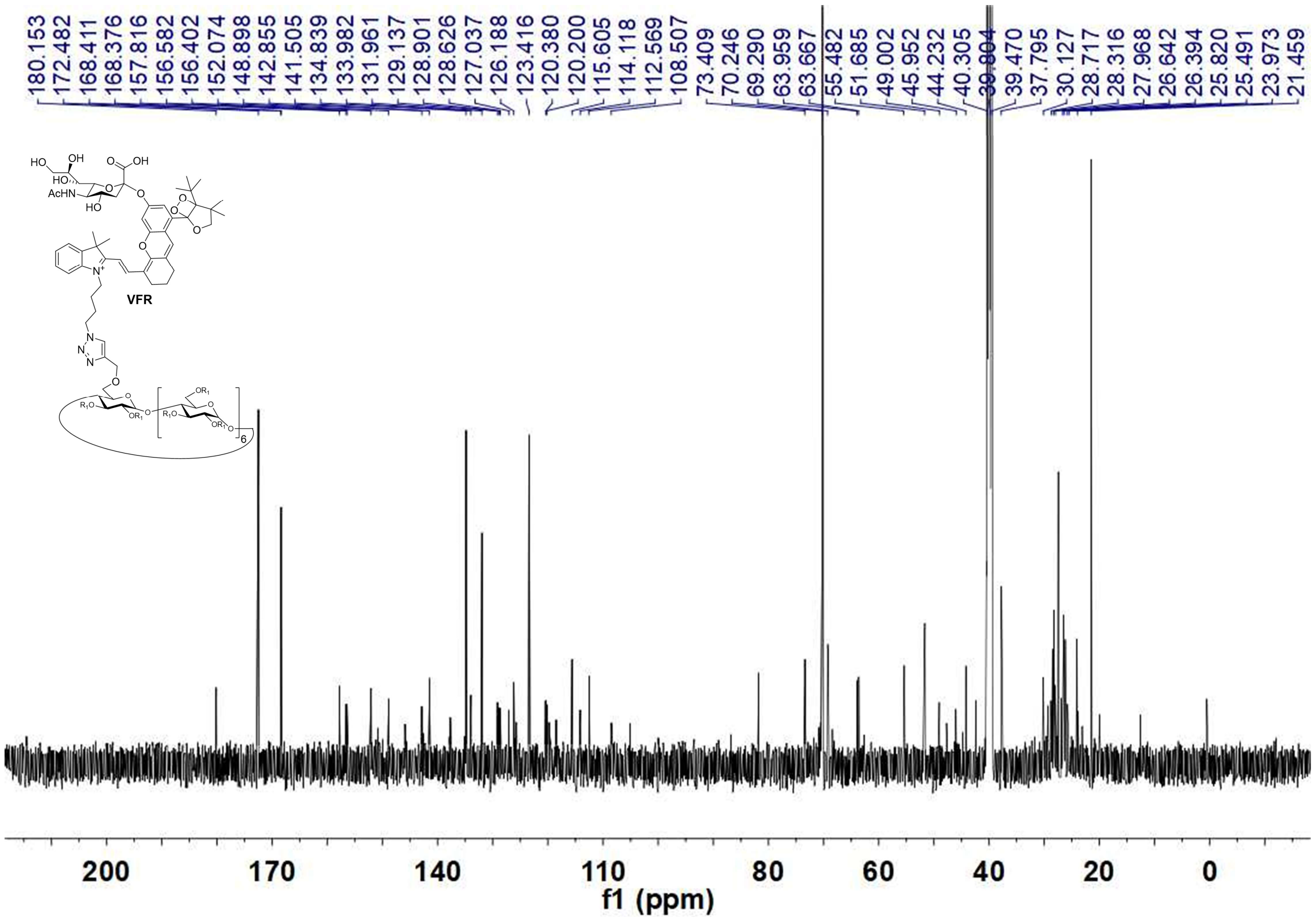

Supplementary Fig. 47. ^{13}C-NMR spectra of compound **VLR.**

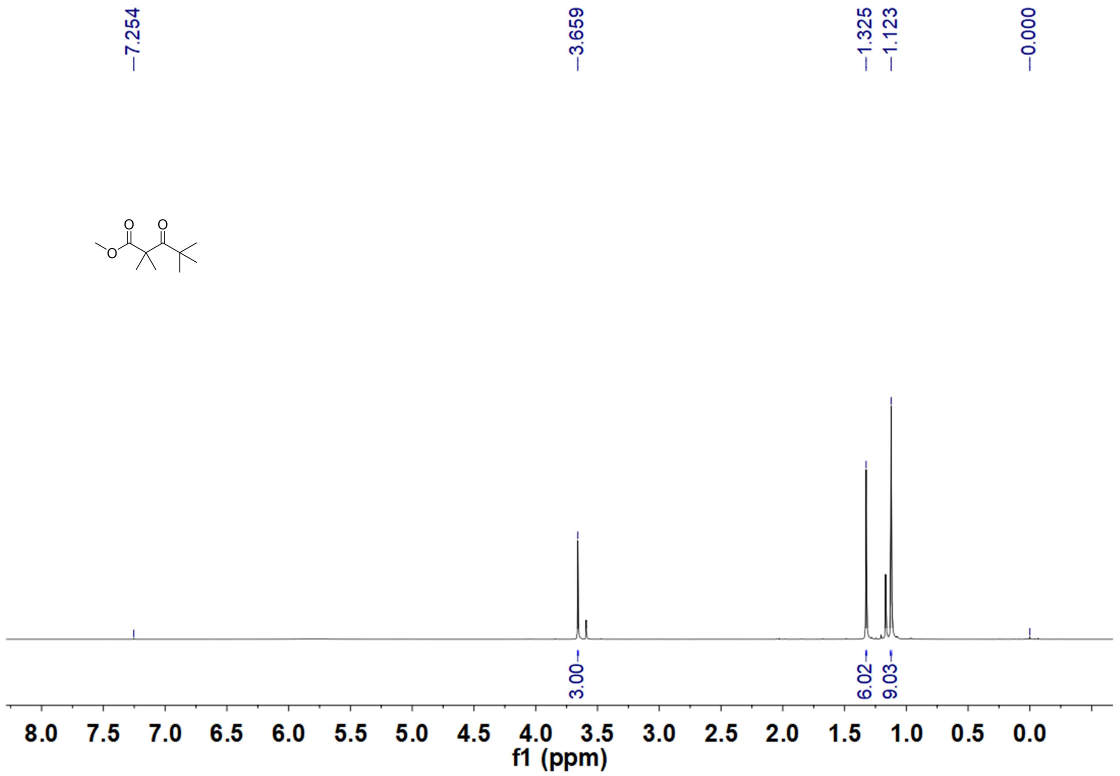

Supplementary Fig. 48. ^{1}H-NMR spectra of compound **8.**

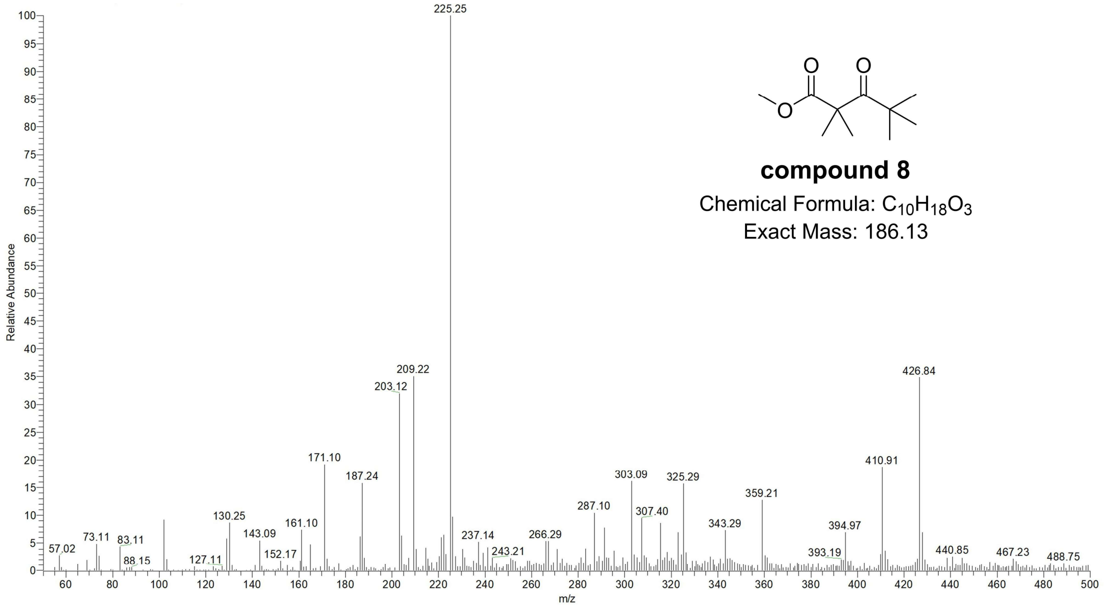

Supplementary Fig. 49. ESI-MS spectra of compound **8.**

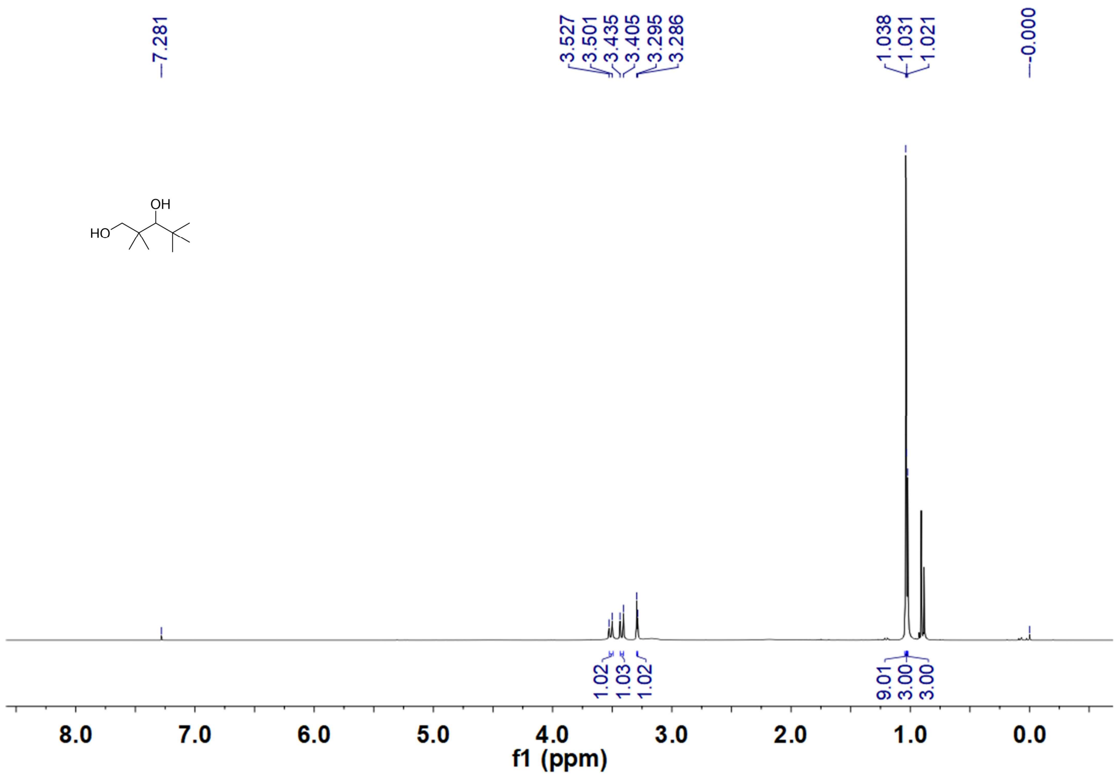

Supplementary Fig. 50. ^{1}H-NMR spectra of compound **9.**

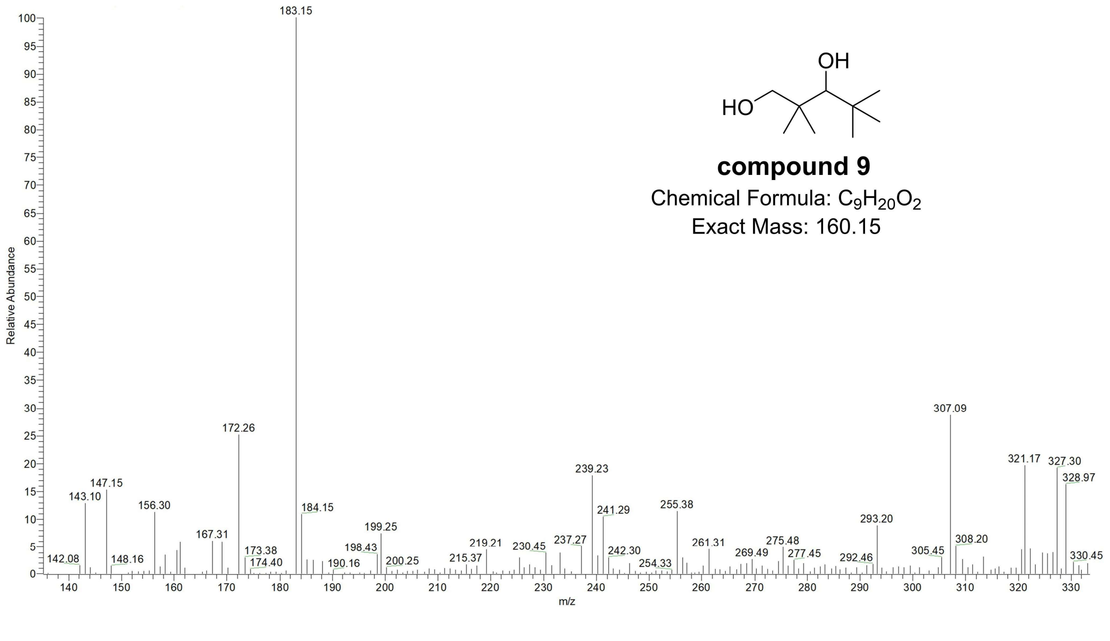

Supplementary Fig. 51. ESI-MS spectra of compound **9.**

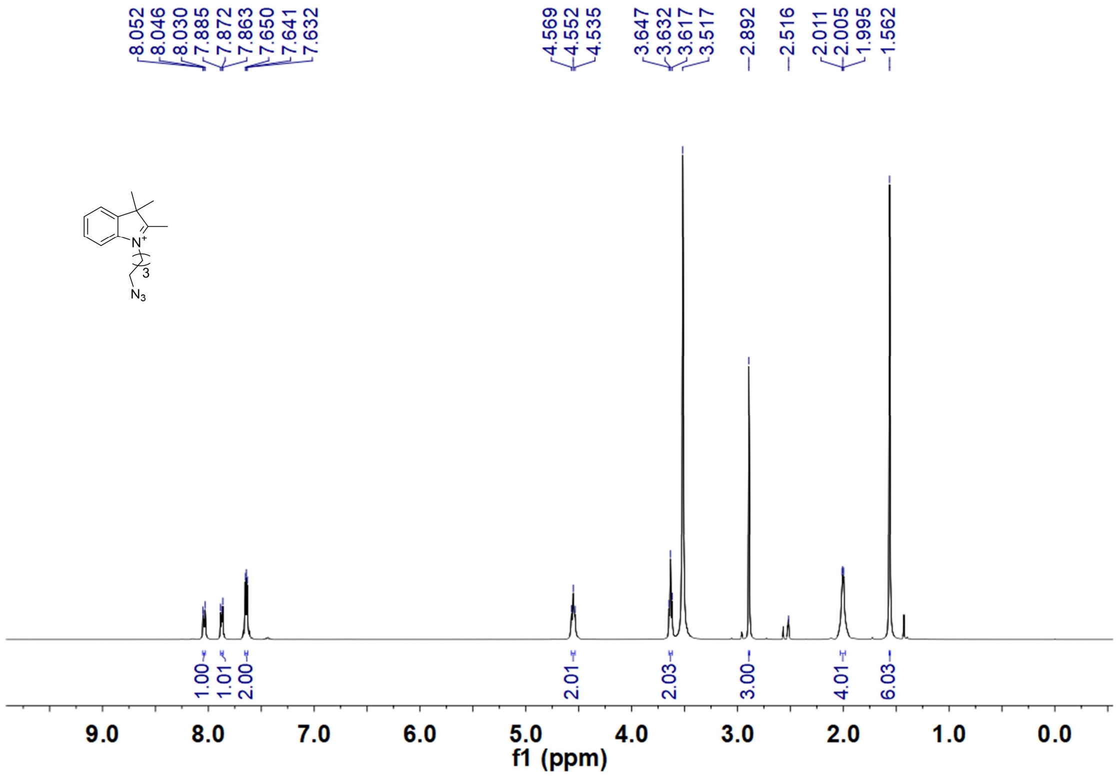

Supplementary Fig. 52. ^{1}H-NMR spectra of compound **11.**

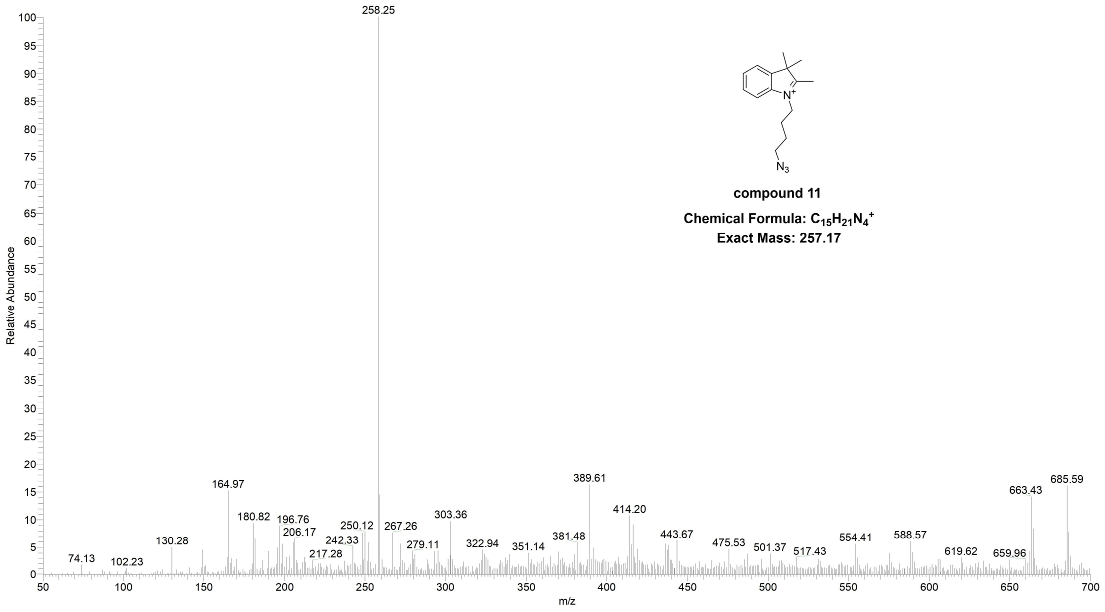

Supplementary Fig. 53. ESI-MS spectra of compound **11.**

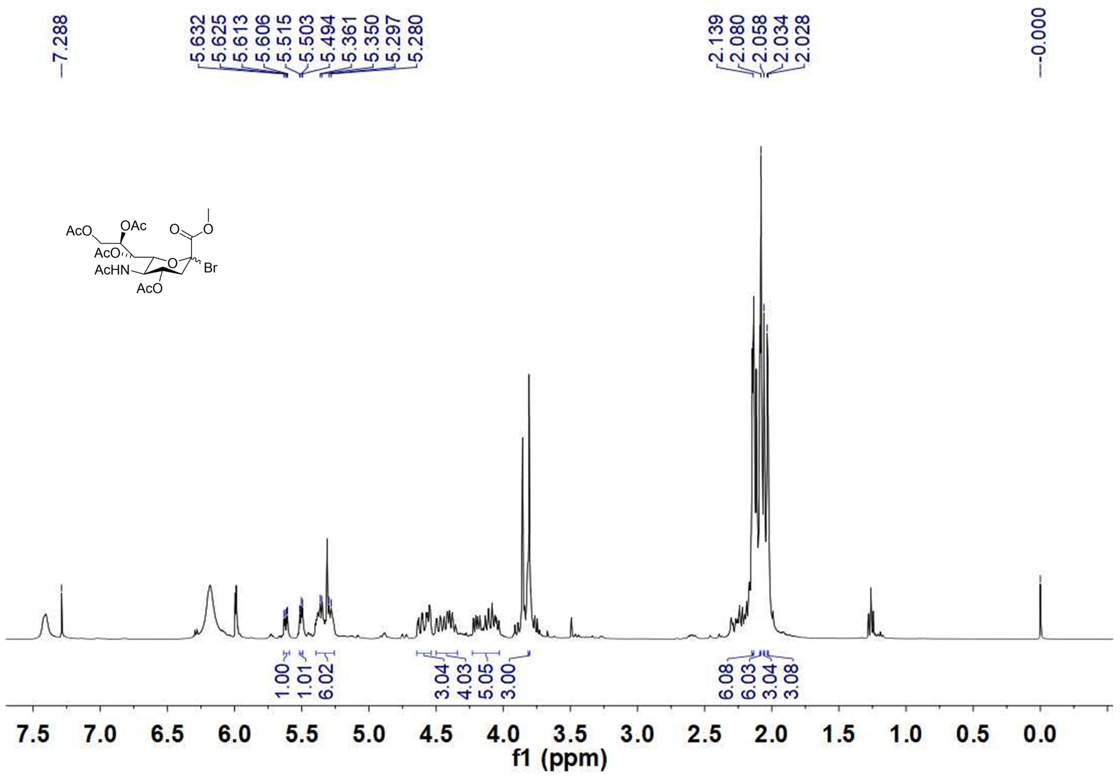

Supplementary Fig. 54. ^{1}H-NMR spectra of compound **13.**

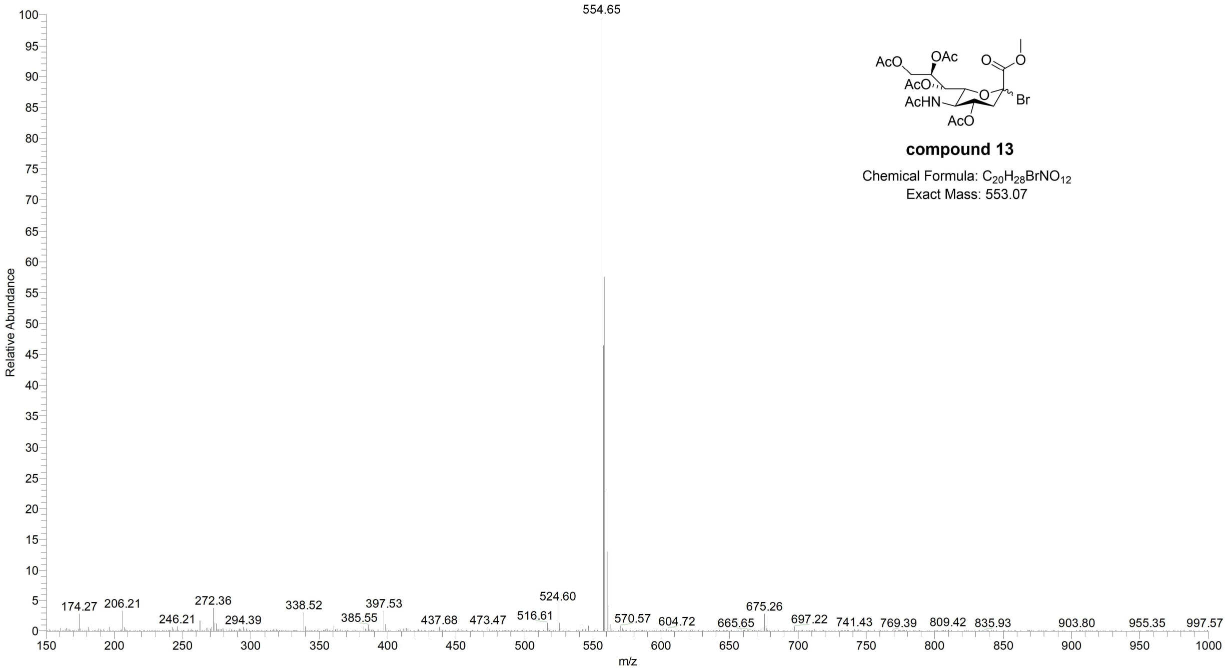

Supplementary Fig. 55. ESI-MS spectra of compound **13.**

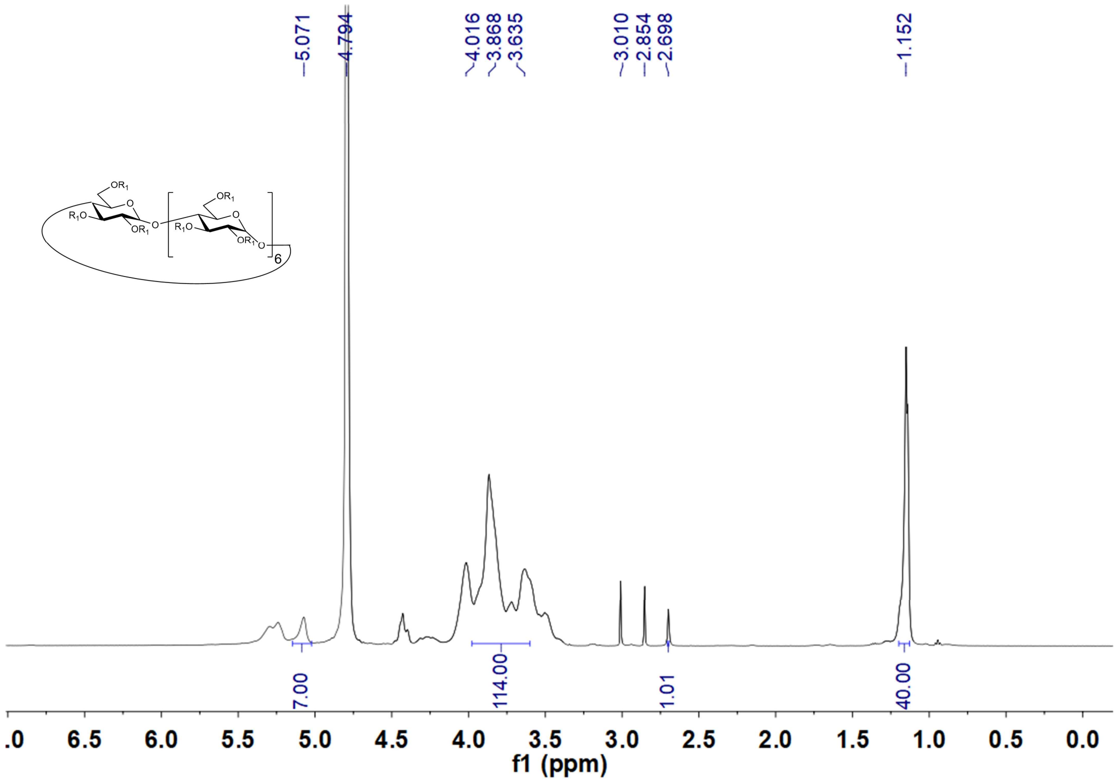

Supplementary Fig. 56. ^{1}H-NMR spectra of compound **HPβCD.**
